# Supplementary material for: A comprehensive neuroanatomical survey of the Drosophila Lobula Plate Tangential Neurons with predictions for their optic flow sensitivity
Source: bioRxiv. 2023 Oct 17:2023.10.16.562634. Preprint. [Version 1] doi: 10.1101/2023.10.16.562634 (PMC10614863; doi:10.1101/2023.10.16.562634)
Supplement: Supplement 1 — Supplementary File 1: Table of reconstructed LPTs, with R-L matches, and correspondences to matched neurons in FlyWire and Hemibrain. [file media-1.pdf]

	Right hemisphere							Left hemisphere					
group	name	CATMAID skid	Hemibrain bodyid	FlyWire ID	FlyWire soma xyz	transmitter		name	CATMAID skid	Hemibrain bodyid	FlyWire ID	FlyWire soma xyz	transmitter
HS	LPT01_R_HSN	830793	2211443902	720575940628031249	92419, 60544, 6514	acetylcholine		LPT01_L_HSN	1088632		720575940615933919	168936, 65983, 5843	acetylcholine
HS	LPT02_R_HSE	827034	1807537598	720575940642723981	98622, 55662, 5601	acetylcholine		LPT02_L_HSE	1088754		720575940629148007	165324, 58221, 5440	acetylcholine
HS	LPT03_R_HSS	4058824	2179731270	720575940622312965	94415, 61273, 6326	acetylcholine		LPT03_L_HSS	3796480		720575940620960347	173701, 59833, 5826	acetylcholine
HST	LPT04_R_HST	985774	5813042688	720575940612296154	90246, 60095, 6567	acetylcholine		LPT04_L_HST	9158488		720575940633182291	172785, 57390, 5750	acetylcholine
VS	LPT05_R_VS1	982897	5813024262	720575940626477498	86605, 61925, 6776	acetylcholine		LPT05_L_VS1	1066332		720575940619878961	180732, 56951, 5935	acetylcholine
VS	LPT06_R_VS2	793032	1868619183	720575940615269794	82996, 59458, 6768	acetylcholine		LPT06_L_VS2	1027307		720575940625331860	175038, 66315, 5995	acetylcholine
VS	LPT07_R_VS3	815776	5813025091	720575940622831740	85204, 54954, 6650	acetylcholine		LPT07_L_VS3	1031609		720575940641812699	176523, 67292, 5996	acetylcholine
VS	LPT08_R_VS4	17686499	1557885051	720575940633017939	82041, 55308, 6788	acetylcholine		LPT08_L_VS4	1038415		720575940659799937	178209, 66353, 6020	acetylcholine
VS	LPT09_R_VS5	807401	5813033533	720575940626457406	85255, 59585, 6818	acetylcholine		LPT09_L_VS5	1037034		720575940639151694	177141, 63573, 6015	acetylcholine
VS	LPT10_R_VS6	804539	1992161820	720575940605688492	84161, 56888, 6776	acetylcholine		LPT10_L_VS6	5053485		720575940626928521	176791, 65544, 5982	acetylcholine
VS	LPT11_R_VS7	851432	1868255513	720575940624931564	89772, 60887, 6632	acetylcholine		LPT11_L_VS7	1040470		720575940618681709	178365, 61493, 6006	acetylcholine
VS	LPT12_R_VS8	804092	1558230909	720575940633923298	80405, 58223, 6579	acetylcholine		LPT12_L_VS8	1034264		720575940636972400	175995, 64465, 5995	acetylcholine
VS	LPT13_R_VSm-1	852286	1836516710	720575940620463307	87866, 58912, 6715	acetylcholine		LPT13_L_VSm-1	1038271		720575940631615545	178690, 65008, 6029	acetylcholine
VS	LPT13_R_VSm-2	815241	1805481901	720575940630825527	80973, 56494, 6842	acetylcholine		LPT13_L_VSm-2	1030967		720575940624500412	175032, 64338, 5995	acetylcholine
VST	LPT15_R_VST1-1	1124867		720575940626947971	89787, 63442, 6664	acetylcholine		LPT15_L_VST1-1	4646358		720575940630700348	172987, 65432, 5938	acetylcholine
VST	LPT15_R_VST1-2	988674	5812993165	720575940645317412	90491, 63121, 6700	acetylcholine		LPT15_L_VST1-2	4909997		720575940620885158	176705, 64882, 6083	acetylcholine
VST	LPT17_R_VST2-1	1123389	1837198715	720575940607420290	88409, 61439, 6677	acetylcholine		LPT17_L_VST2-1	4930213		720575940626690078	175782, 61463, 5953	acetylcholine
VST	LPT17_R_VST2-2	1112633	5813023313	720575940646114926	86093, 56825, 6691	acetylcholine		LPT17_L_VST2-2	1059146		720575940624981908	177331, 58779, 5908	acetylcholine
VST	LPT17_R_VST2-3	2852912	5813049185	720575940635615339	87772, 60990, 6701	acetylcholine		LPT17_L_VST2-3	3526291		720575940622939101	176251, 59718, 5901	acetylcholine
VST	LPT17_R_VST2-4	5031615	5813023581	720575940628452520	84643, 62966, 6845	acetylcholine		LPT17_L_VST2-4	1058778		720575940624695275	174516, 66583, 5944	acetylcholine
Ipsilateral	LPT21_R	1110765	1850310331	720575940610781560	91734, 46381, 5476	acetylcholine		LPT21_L	7764572		720575940627341736	167934, 53014, 4893	acetylcholine
Ipsilateral	LPT22_R	3510999	1501708149	720575940610061763	97775, 46462, 5376	gaba		LPT22_L	17077102		720575940628685099	165247, 52598, 4767	gaba
Ipsilateral	LPT23_R-1	4135042	1842090544	720575940617304949	94555, 50477, 5512	acetylcholine		LPT23_L-1	17053936		720575940621790017	167897, 56581, 4842	acetylcholine
Ipsilateral	LPT23_R-2	4504548	1751364895	720575940632545080	95706, 51204, 5502	acetylcholine		LPT23_L-2	17092277		720575940645636887	168672, 53867, 4835	acetylcholine
Ipsilateral	LPT23_R-3	4504557	1751028010	720575940619638395	93785, 48704, 5586	acetylcholine		LPT23_L-3	17076395		720575940630171642	168507, 55106, 4878	acetylcholine
Ipsilateral	LPT26_R	1107045	1747289181	720575940630691895	95022, 67847, 4005	acetylcholine		LPT26_L	4702640		720575940625992073	162875, 73838, 3644	acetylcholine
Ipsilateral	LPT27_R	3514698	1747608690	720575940630990300	94413, 68614, 3895	acetylcholine		LPT27_L	7937241		720575940634371996	164593, 72304, 3618	acetylcholine
Ipsilateral	LPT28_R	1071860	1407904797	720575940620898324	87242, 41135, 4875	acetylcholine		LPT28_L	4970951		720575940631468485	176196, 44172, 4262	acetylcholine
Ipsilateral	LPT29_R	7760435	5813087982	720575940632216899	91436, 65820, 4007	acetylcholine		LPT29_L	17096943		720575940633500185	163367, 69763, 5142	acetylcholine
Ipsilateral	LPT30_R	7674107	1777301335	720575940624047654	81985, 59904, 3607	acetylcholine		LPT30_L	7701590		720575940621204673	180205, 62796, 2749	acetylcholine
Posterial	LPT31_R-1	7690273	1622471963	720575940634166242	89099, 65661, 3909	acetylcholine		LPT31_L-1	7841427		720575940623263453	175392, 68820, 2642	acetylcholine
Posterial	LPT31_R-2	7311408	1434427171	720575940616260146	89798, 66530, 3874	acetylcholine		LPT31_L-2	7841256		720575940622087429	172799, 69268, 2782	acetylcholine
Posterial	LPT31_R-3	7311493	1434427905	720575940629577475	89987, 68909, 3709	acetylcholine		LPT31_L-3	8035397		720575940607401138	177419, 65843, 2633	acetylcholine
Posterial	LPT31_R-4	7694305	1344082025	720575940629567055	88553, 66092, 3832	acetylcholine		LPT31_L-4	8035389		720575940629012240	174034, 68456, 2659	acetylcholine
CH	LPT35_R_dCH	1077174	1466485353	720575940636933751	131387, 52610, 1235	gaba		LPT35_L_dCH	6243409	1545158404	720575940628391848	125941, 49686, 1261	gaba
CH	LPT36_R_vCH	1078535	5813024201	720575940627138562	133918, 52245, 838	gaba		LPT36_L_vCH	1078048		720575940634274017	129340, 51844, 1347	gaba
H1	LPT37_R_H1	1121730	676832896	720575940660765569	176884, 43788, 2168	glutamate		LPT37_L_H1	1121335	1167783603	720575940627348617	92329, 43685, 2675	glutamate
Noduli	LPT38_R_Nod1-1	1054753	5812996970	720575940609132043	101804, 63992, 5521	acetylcholine		LPT38_L_Nod1-1	6439204	1789306586	720575940623997949	160483, 67115, 5219	acetylcholine
Noduli	LPT38_R_Nod1-2	3546483	5812993603	720575940628438427	94671, 46204, 5314	acetylcholine		LPT38_L_Nod1-2	6437945	1758621675	720575940629456860	168023, 48186, 4930	acetylcholine
Noduli	LPT40_R_Nod2	902072	1315529069	720575940623235683	107626, 47015, 5121	gaba		LPT40_L_Nod2	16615842	1871778911	720575940625670949	157474, 51216, 4838	gaba
Noduli	LPT41_R_Nod3	1058996	1352706891	720575940623384781	100729, 52368, 5430	acetylcholine		LPT41_L_Nod3	15060018	1758617327	720575940611869849	164666, 53244, 4966	acetylcholine
Noduli	LPT42_R_Nod4	1106958	1566524544	720575940625992781	108161, 62214, 5644	acetylcholine		LPT42_L_Nod4	16615419	1725837767	720575940619612142	159693, 68616, 5133	acetylcholine
Noduli	LPT43_R_H2	1088678	1534124048	720575940627079938	104111, 49333, 5272	acetylcholine		LPT43_L_H2	5232902	5813078454	720575940632427603	164234, 55937, 5175	acetylcholine
Noduli	LPT44_R_Nod5	1121795	1899956171	720575940633685459	98853, 47380, 5337	acetylcholine		LPT44_L_Nod5	7231304	5812994338	720575940620431808	164930, 52607, 5201	acetylcholine
Calyx	LPT45_R_dCal1	7204844	943813788	720575940628350997	86606, 64556, 6802	gaba		LPT45_L_dCal1	3509763	5813057267	720575940629288271	166160, 69437, 5555	gaba
Calyx	LPT46_R_vCal1	7510076	1005174931	720575940641223888	92000, 41560, 5217	glutamate		LPT46_L_vCal1	8747266	943468720	720575940624943504	169463, 47531, 4854	glutamate
Calyx	LPT47_R_vCal2	3529071	1005174975	720575940618653524	89976, 42233, 5046	glutamate		LPT47_L_vCal2	7449616	943472755	720575940649181817	169732, 45848, 4848	glutamate
Calyx	LPT48_R_vCal3	1056097	974502819	720575940626919780	98267, 43905, 5284	acetylcholine		LPT48_L_vCal3	11230125	943472763	720575940626630693	164609, 51684, 4749	acetylcholine
Bilateral	LPT49_R	1110693	1654969939	720575940638155998	81754, 59034, 3645	acetylcholine		LPT49_L	17072553	1685896788	720575940629791643	180014, 63132, 2609	acetylcholine
Bilateral	LPT50_R	905761	1496497366	720575940655599777	102903, 50290, 5211	gaba		LPT50_L	3503997	5813049974	720575940611348834	164426, 56537, 5078	gaba
Ipsilateral	LPT51_R	4224711	1313496323	720575940608287701	96820, 68844, 4150	glutamate		LPT51_L	17083003		720575940621153670	165710, 62759, 4543	glutamate
Ipsilateral	LPT52_R	1107296	1469291436	720575940640716928	102698, 64957, 5710	acetylcholine		LPT52_L	17039407		720575940621164532	164302, 60961, 5020	acetylcholine
Ipsilateral	LPT53_R	1111992	1231610379	720575940653093110	103717, 51483, 5709	gaba		LPT53_L	17072462		720575940629704210	162429, 54039, 5238	gaba
Ipsilateral	LPT54_R	4235388	1129033939	720575940655602849	100006, 56145, 5476	acetylcholine		LPT54_L	17196151		720575940632066578	163177, 58561, 5143	acetylcholine
Bilateral	LPT55_R_MeLp2	1061368	1558226341	720575940617422731	182002, 56164, 2020	glutamate		LPT55_L_MeLo2	17652973		720575940622735649	79037, 46479, 3736	glutamate
Bilateral	LPT56_R_MeLp1	3509520	5813068976	720575940655602849	88337, 67218, 3555	acetylcholine		LPT56_L_MeLo1	4224594	5813063227	720575940632061738	171781, 75310, 2802	acetylcholine
Bilateral	LPT57_R	886797	5901198180	720575940632041746	132727, 42772, 4246	acetylcholine		LPT57_L	17059178	5813045086	720575940619709342	128014, 38440, 4550	acetylcholine
Bilateral	LPT58_R_V1	1059420		720575940621291873	136567, 34979, 4354	acetylcholine		LPT58_L_V1	9539098		720575940622275199	131179, 29675, 3714	acetylcholine
