# Supplementary material for: A comprehensive neuroanatomical survey of the Drosophila Lobula Plate Tangential Neurons with predictions for their optic flow sensitivity
Source: bioRxiv. 2023 Oct 17:2023.10.16.562634. Preprint. [Version 1] doi: 10.1101/2023.10.16.562634 (PMC10614863; doi:10.1101/2023.10.16.562634)
Supplement: Supplement 3 — Supplementary File 3: Gallery of all right-side LPTs reconstructed in FAFB and all matched left-side LPTs from FlyWire. [file media-3.pdf]

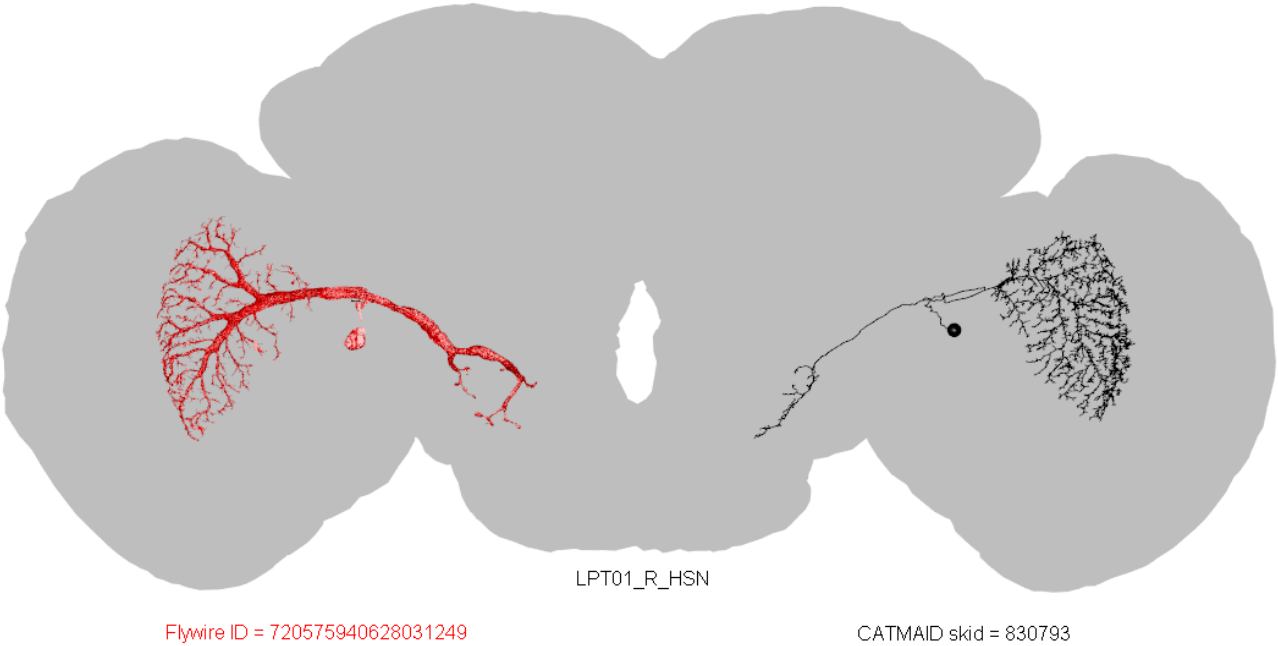
LPT01_R_HSN
Flywire ID = 720575940628031249
CATMAID skid = 830793

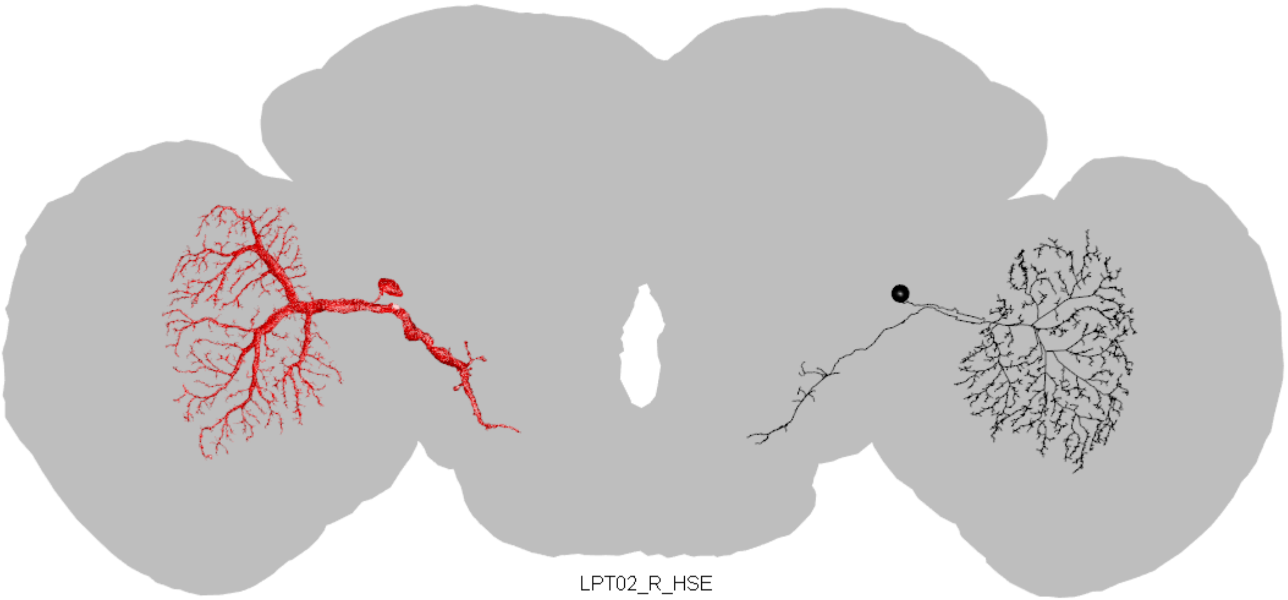

LPT02_R_HSE

Flywire ID = 720575940642723981

CATMAID skid = 827034

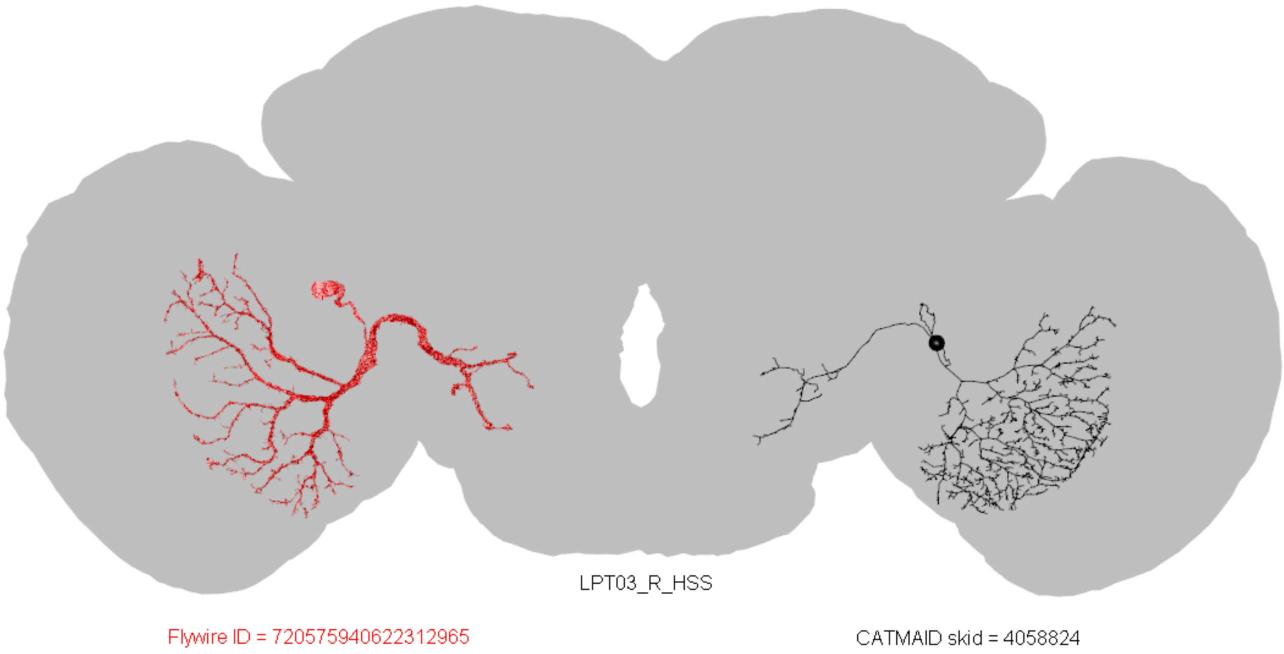

LPT03_R_HSS
Flywire ID = 720575940622312965
CATMAID skid = 4058824

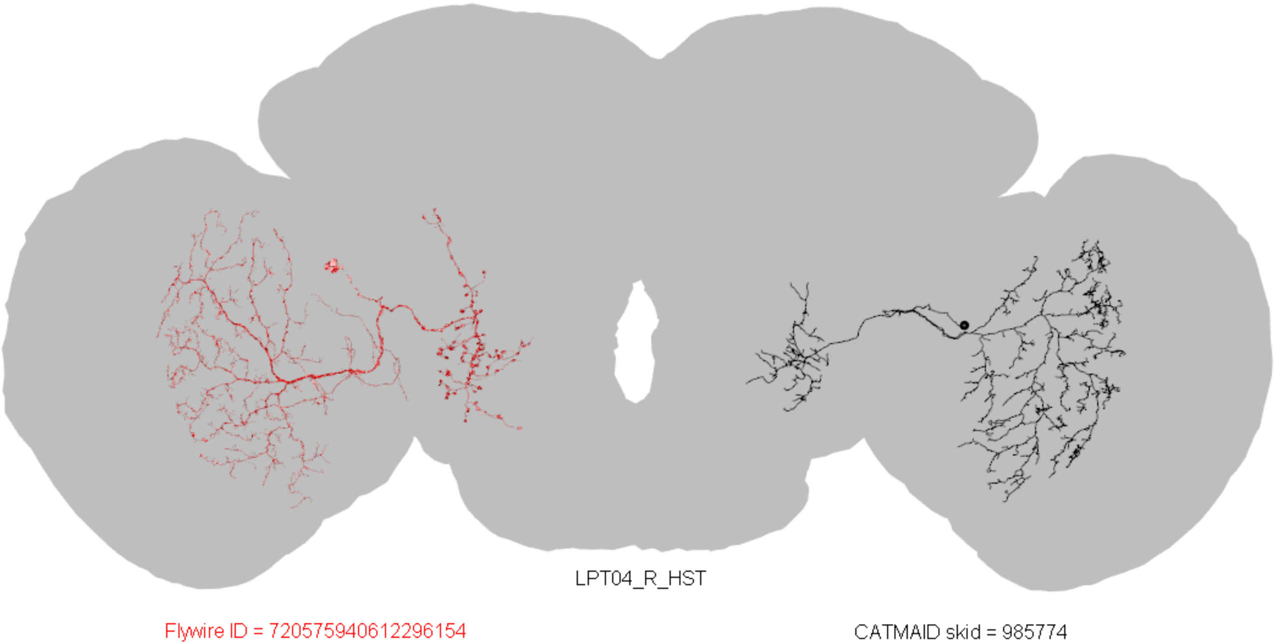
LPT04_R_HST
Flywire ID = 720575940612296154
CATMAID skid = 985774

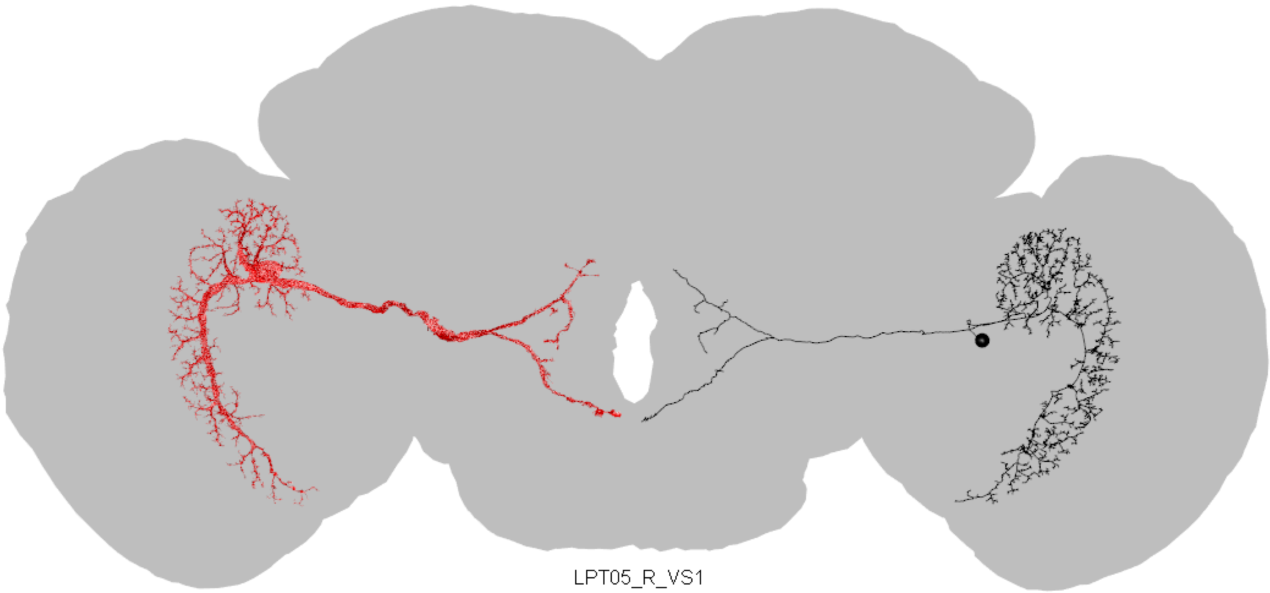

LPT05_R_VS1

Flywire ID = 720575940626477498

CATMAID skid = 982897

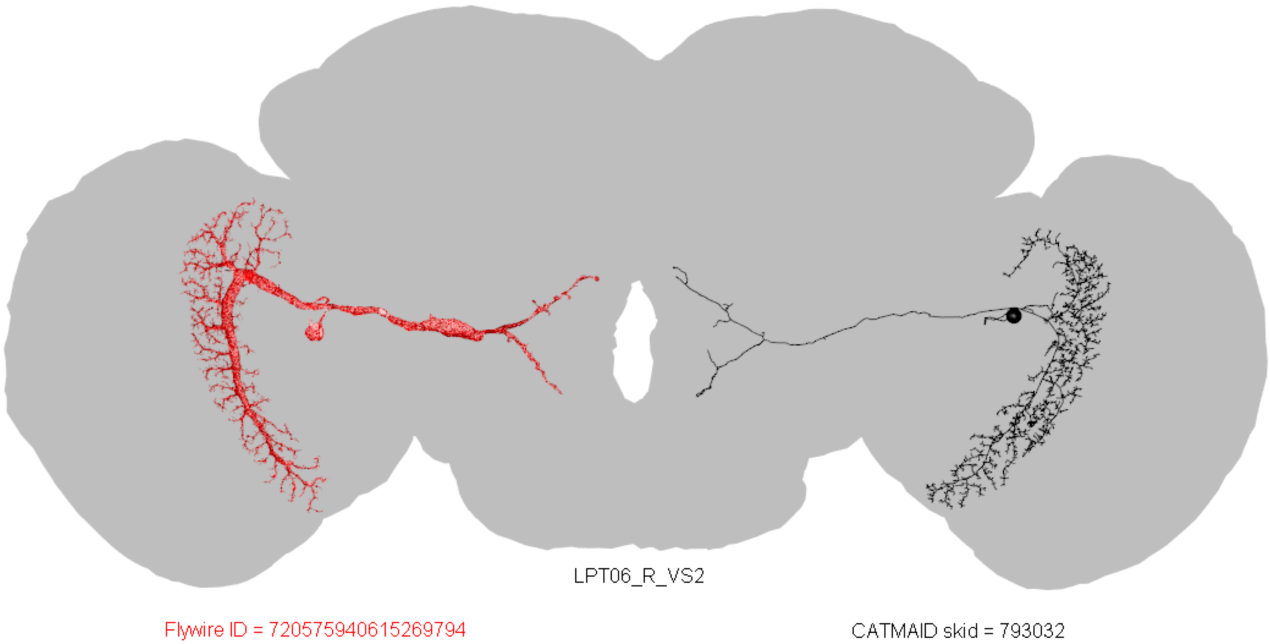

LPT06_R_VS2
Flywire ID = 720575940615269794
CATMAID skid = 793032

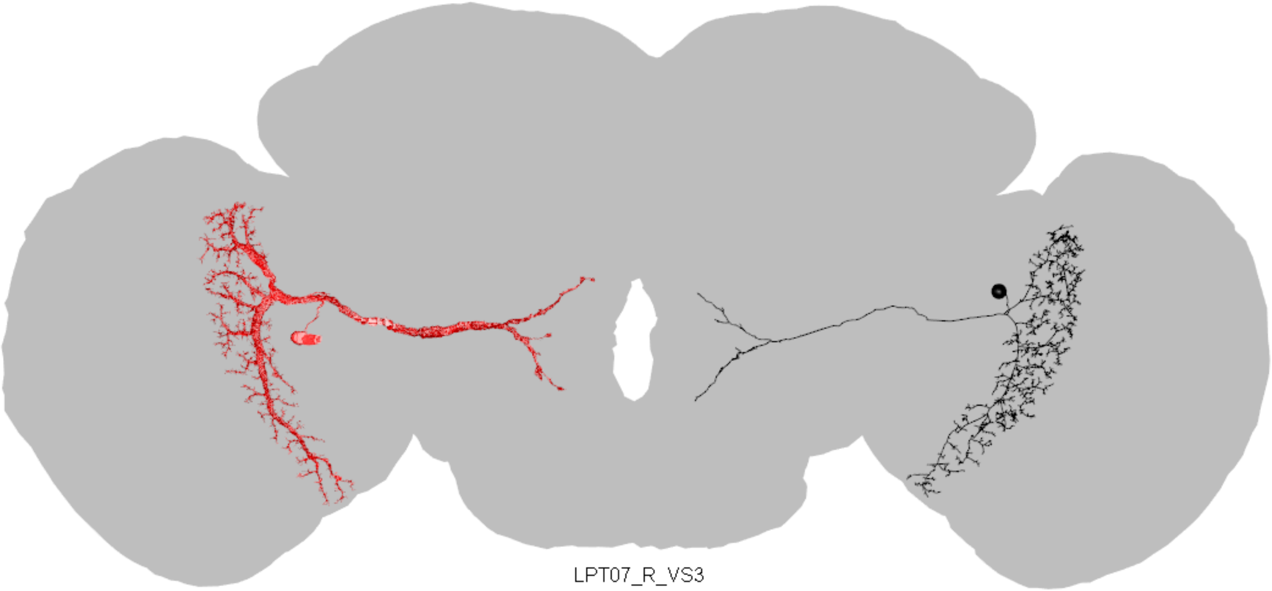

LPT07_R_VS3

Flywire ID = 720575940622831740

CATMAID skid = 815776

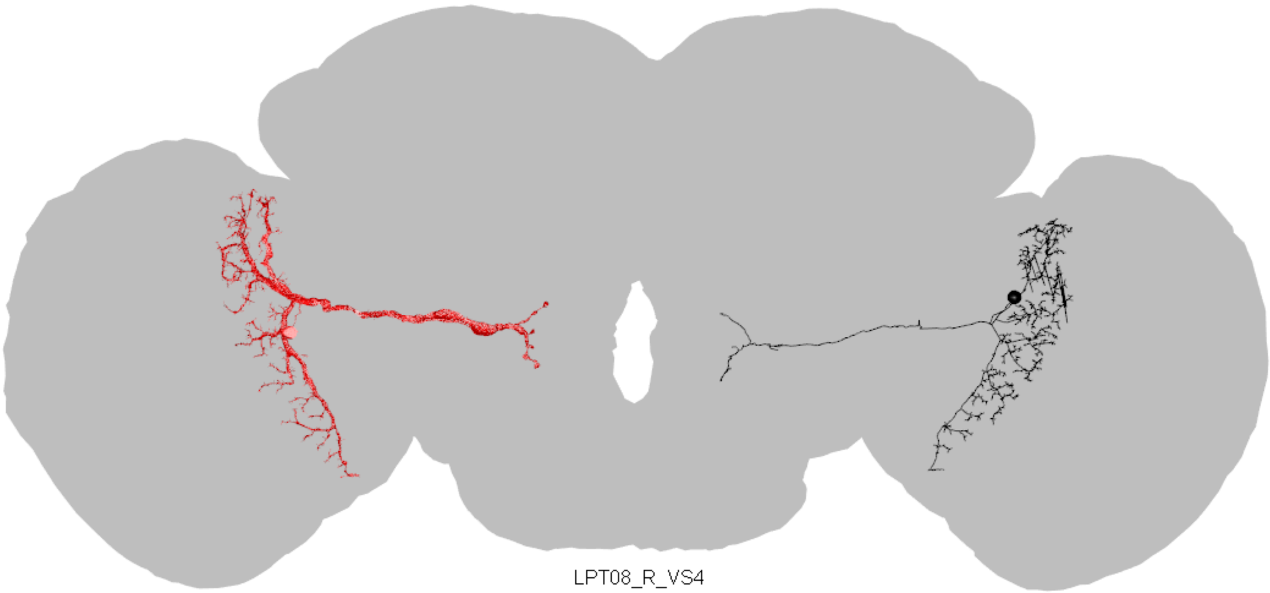

Flywire ID = 720575940633017939

CATMAID skid = 17686499

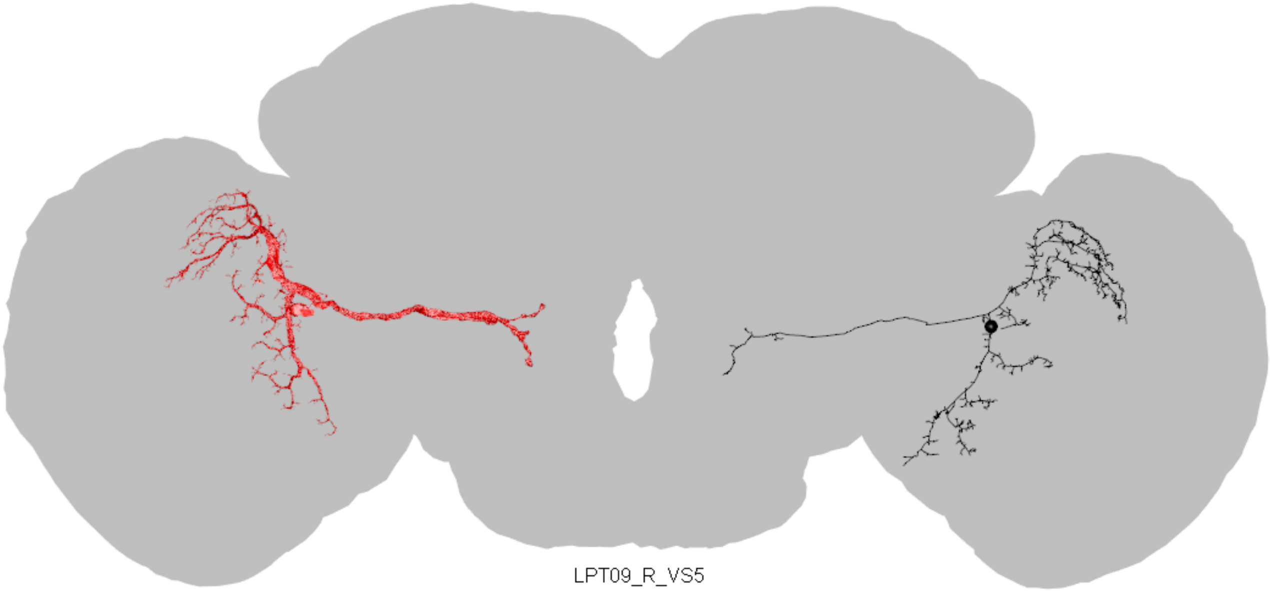

LPT09_R_VS5

Flywire ID = 720575940626457406

CATMAID skid = 807401

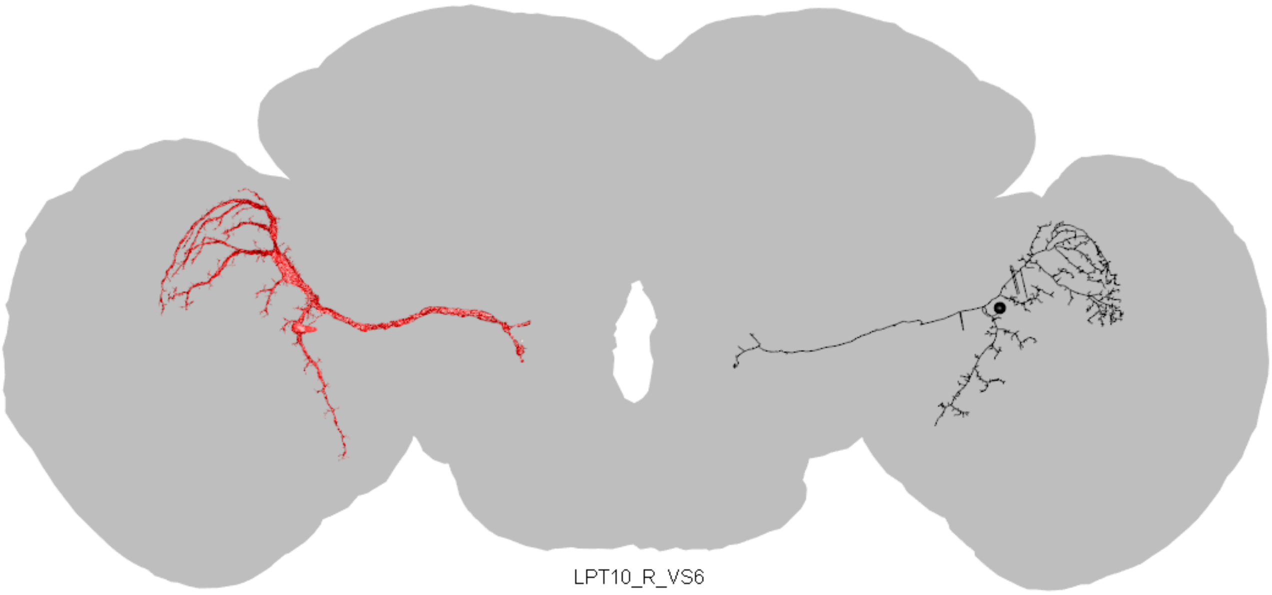

LPT10_R_VS6

Flywire ID = 720575940605688492

CATMAID skid = 804539

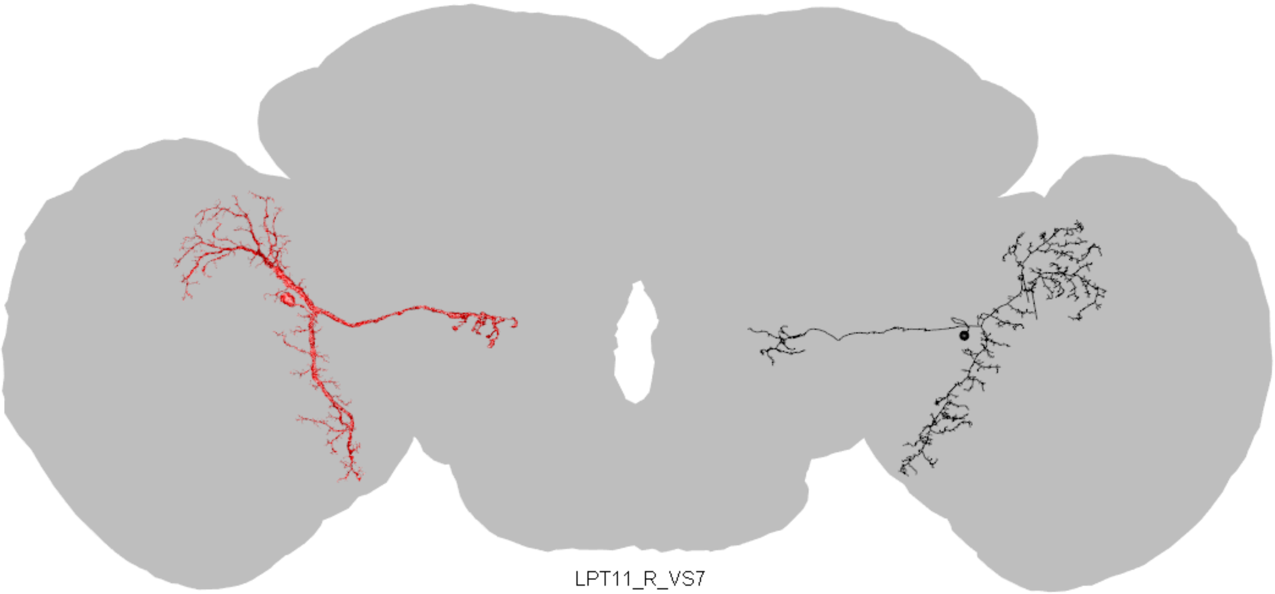

LPT11_R_VS7

Flywire ID = 720575940624931564

CATMAID skid = 851432

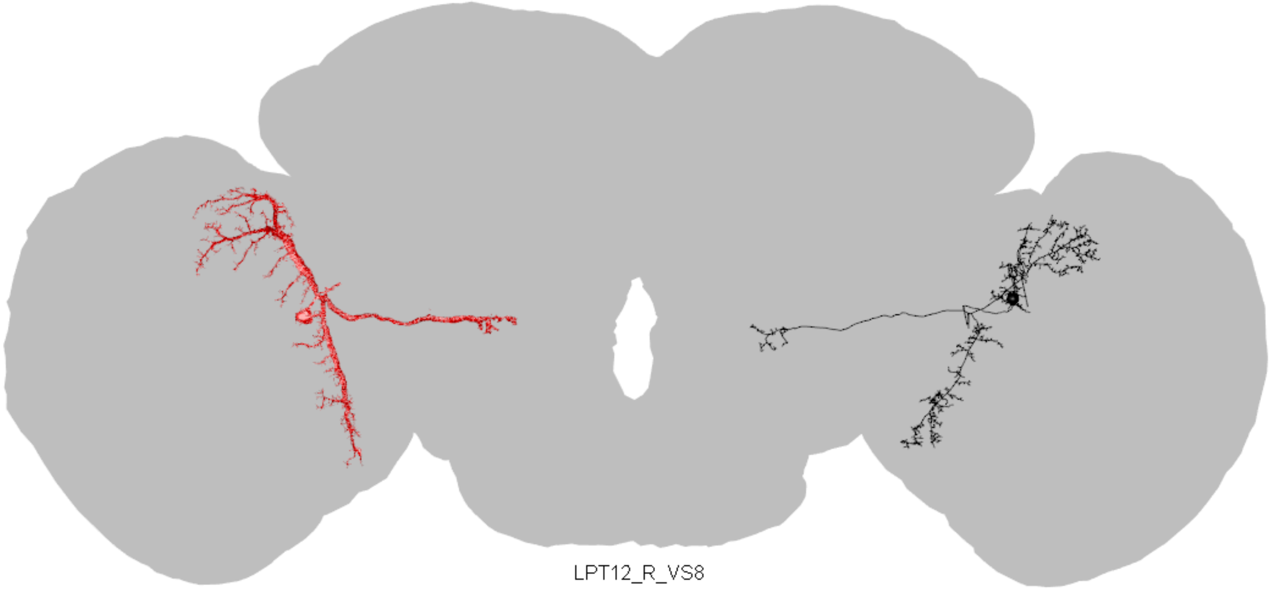

LPT12_R_VS8

Flywire ID = 720575940633923298

CATMAID skid = 804092

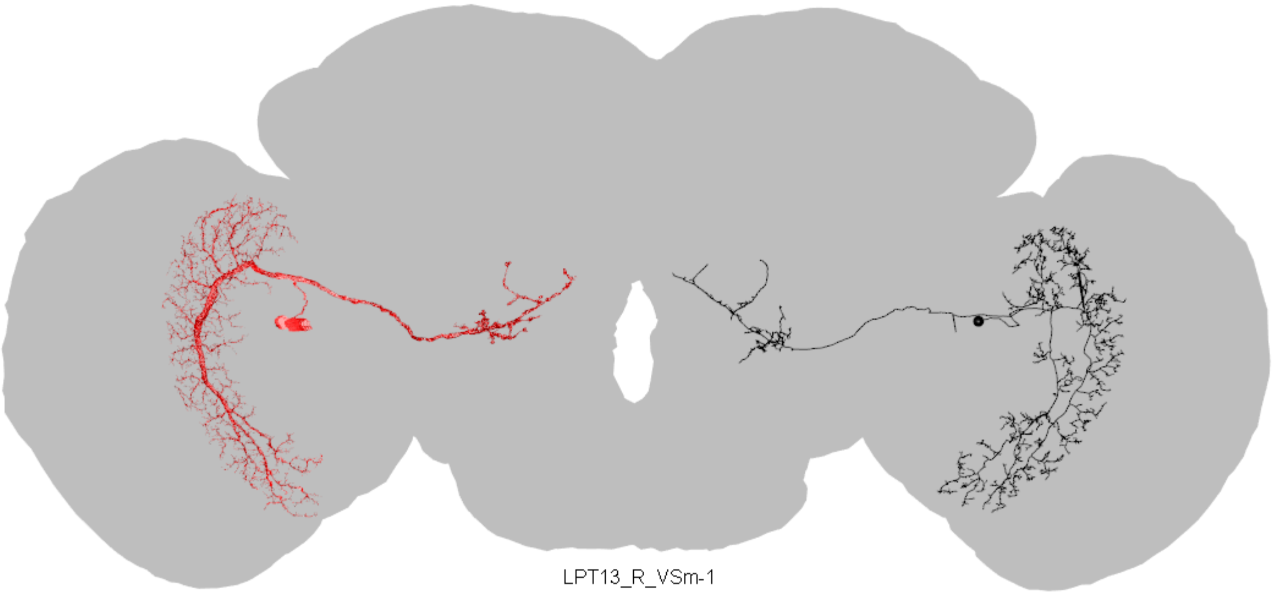

LPT13_R_VSm-1

Flywire ID = 720575940620463307

CATMAID skid = 852286

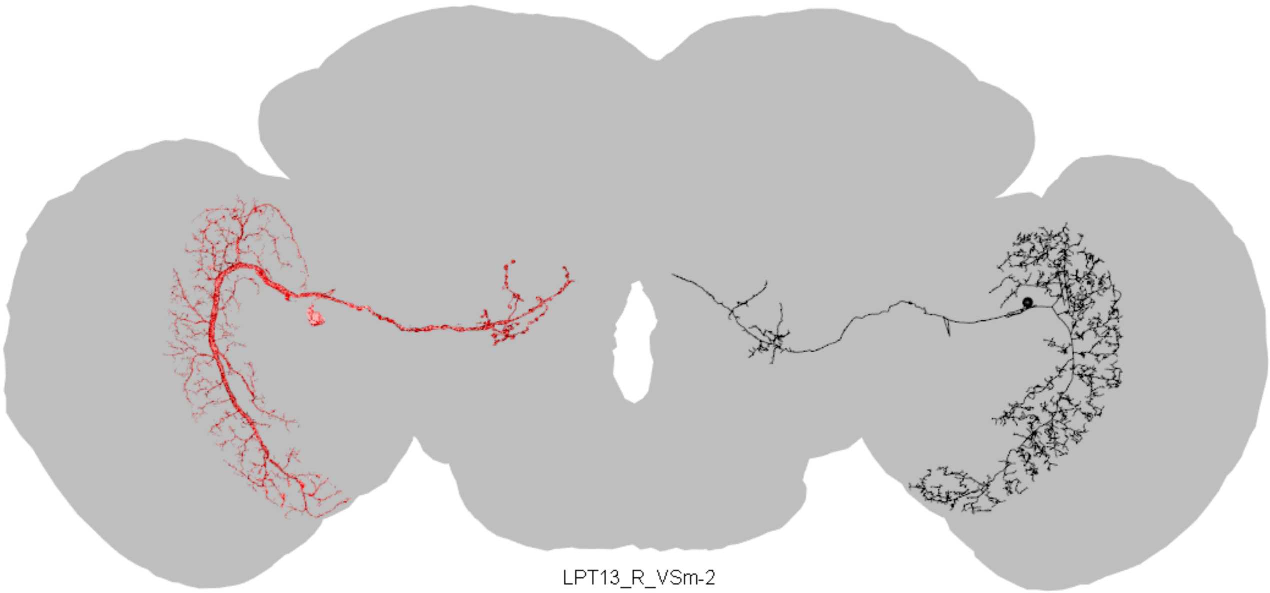

LPT13_R_VSm-2

Flywire ID = 720575940630825527

CATMAID skid = 815241

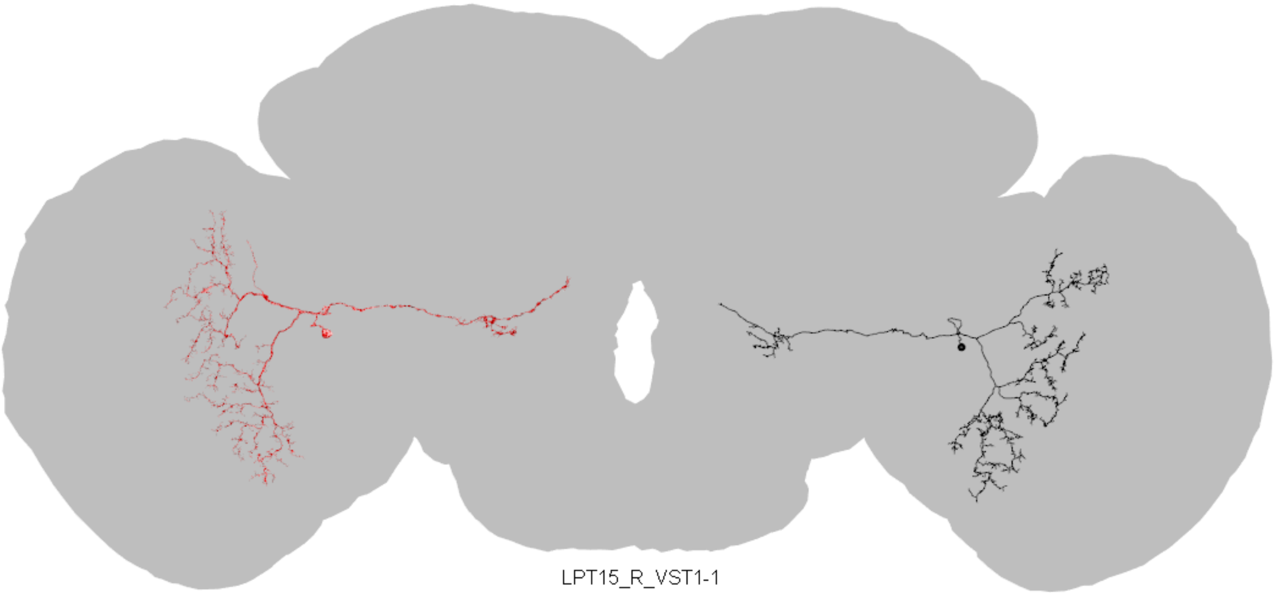

LPT15_R_VST1-1

Flywire ID = 720575940626947971

CATMAID skid = 1124867

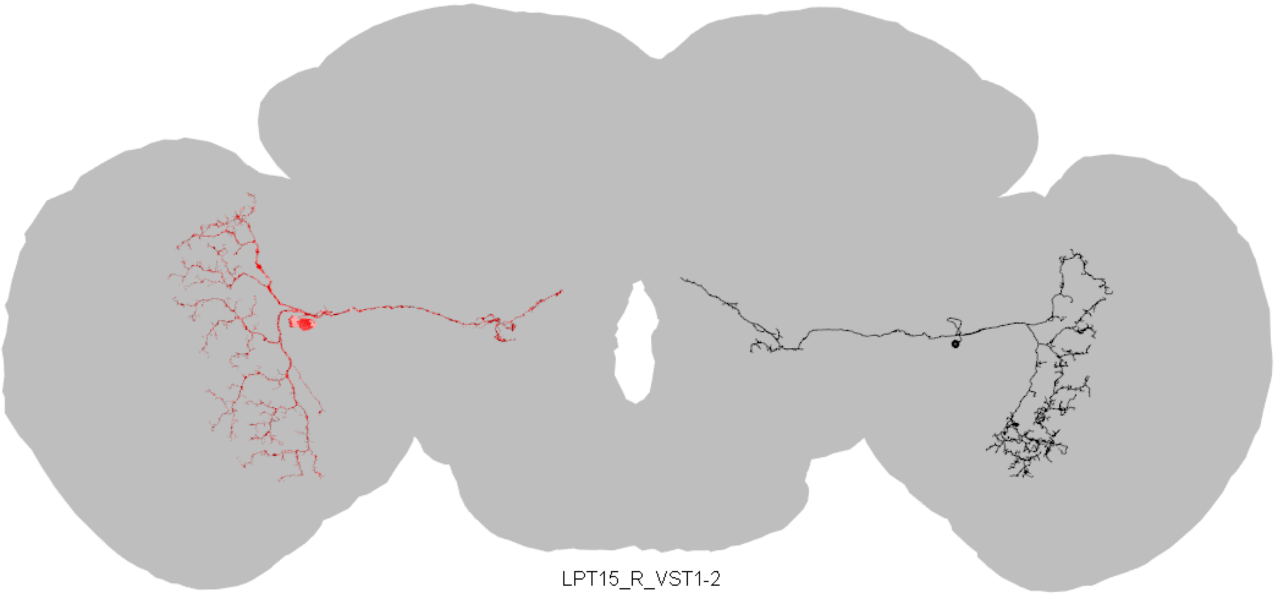

LPT15_R_VST1-2

Flywire ID = 720575940645317412

CATMAID skid = 988674

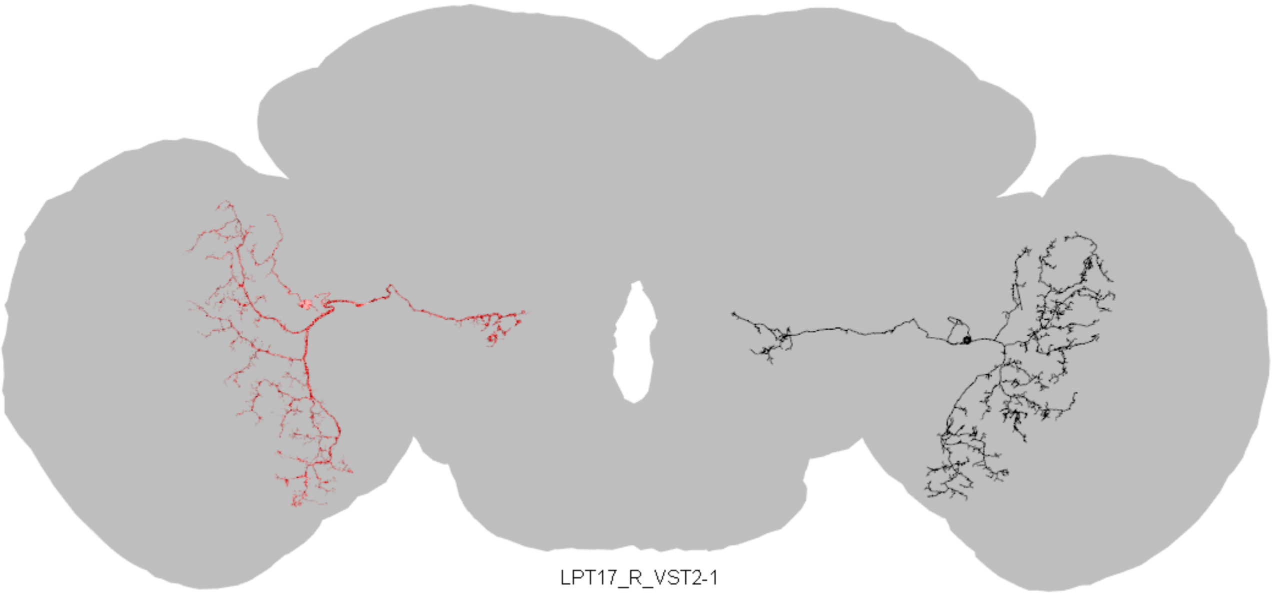

LPT17_R_VST2-1

Flywire ID = 720575940607420290

CATMAID skid = 1123389

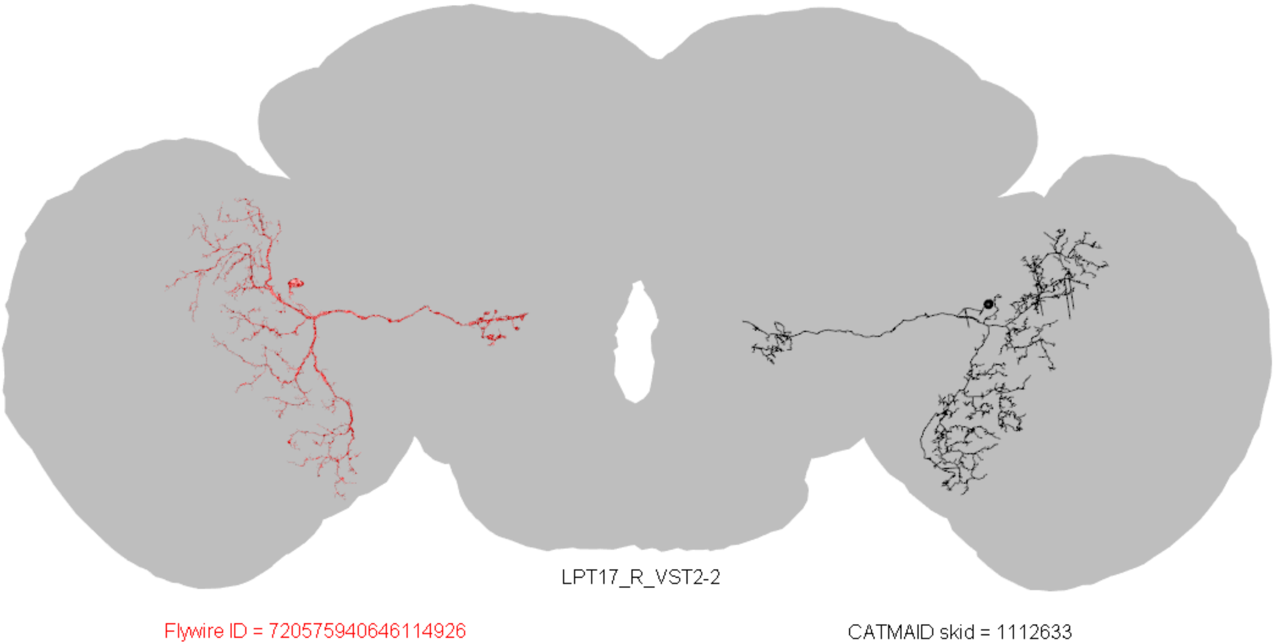
LPT17_R_VST2-2
Flywire ID = 720575940646114926
CATMAID skid = 1112633

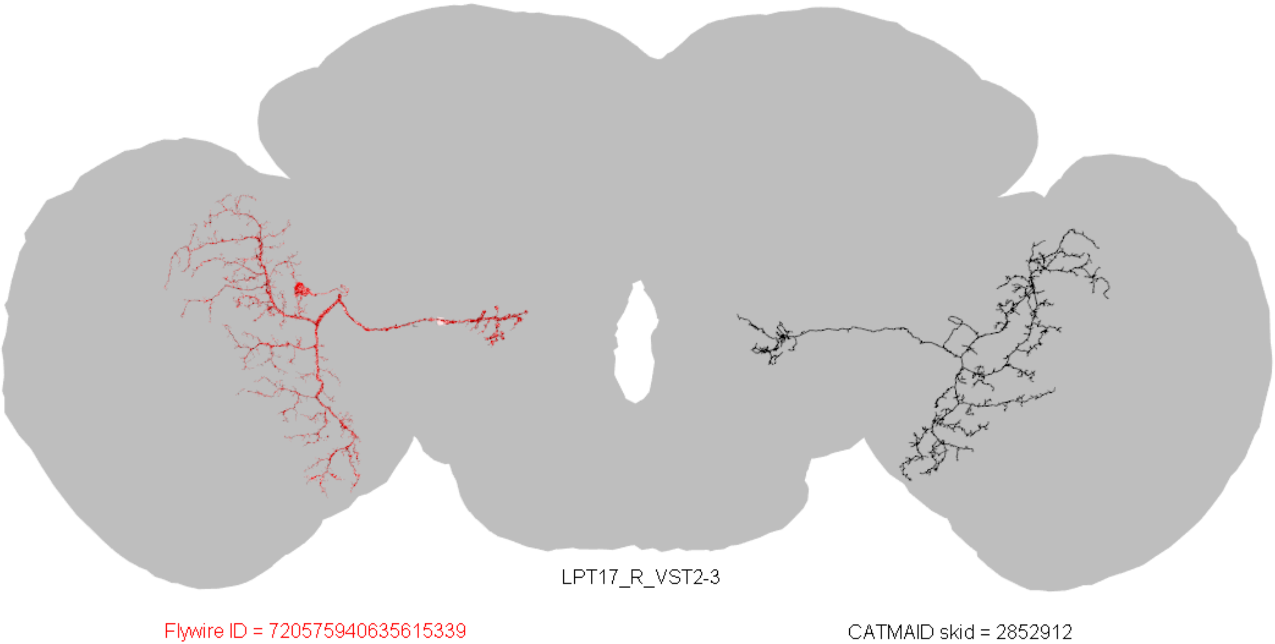
LPT17_R_VST2-3
Flywire ID = 720575940635615339
CATMAID skid = 2852912

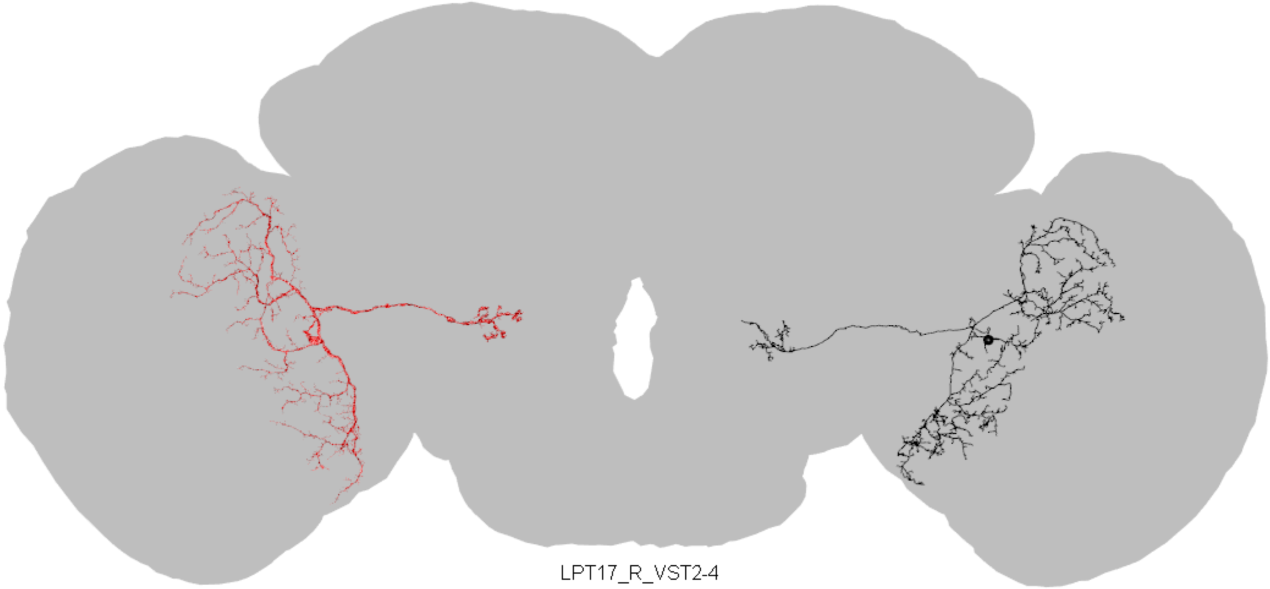

LPT17_R_VST2-4

Flywire ID = 720575940628452520

CATMAID skid = 5031615

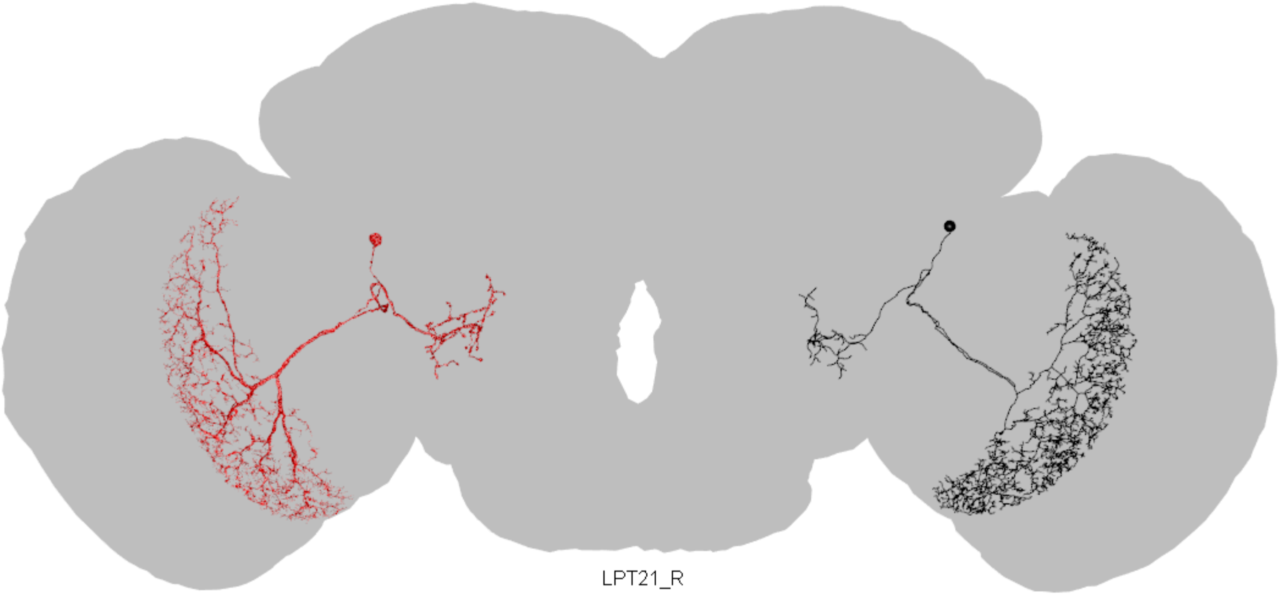

LPT21_R

Flywire ID = 720575940610781560

CATMAID skid = 1110765

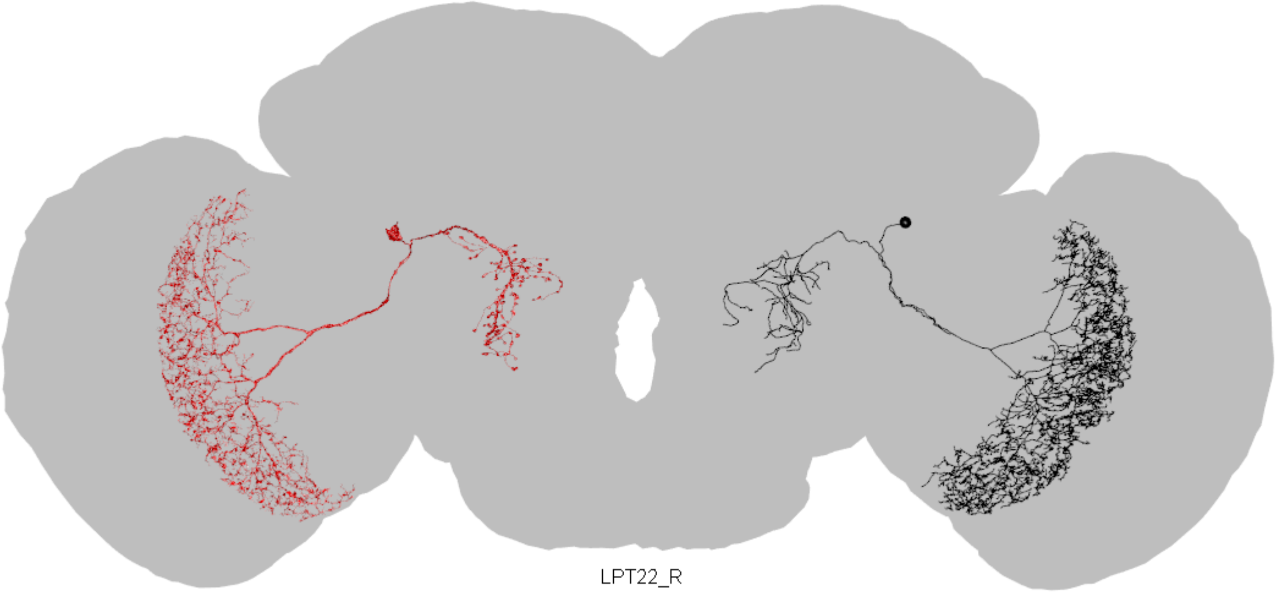
LPT22_R

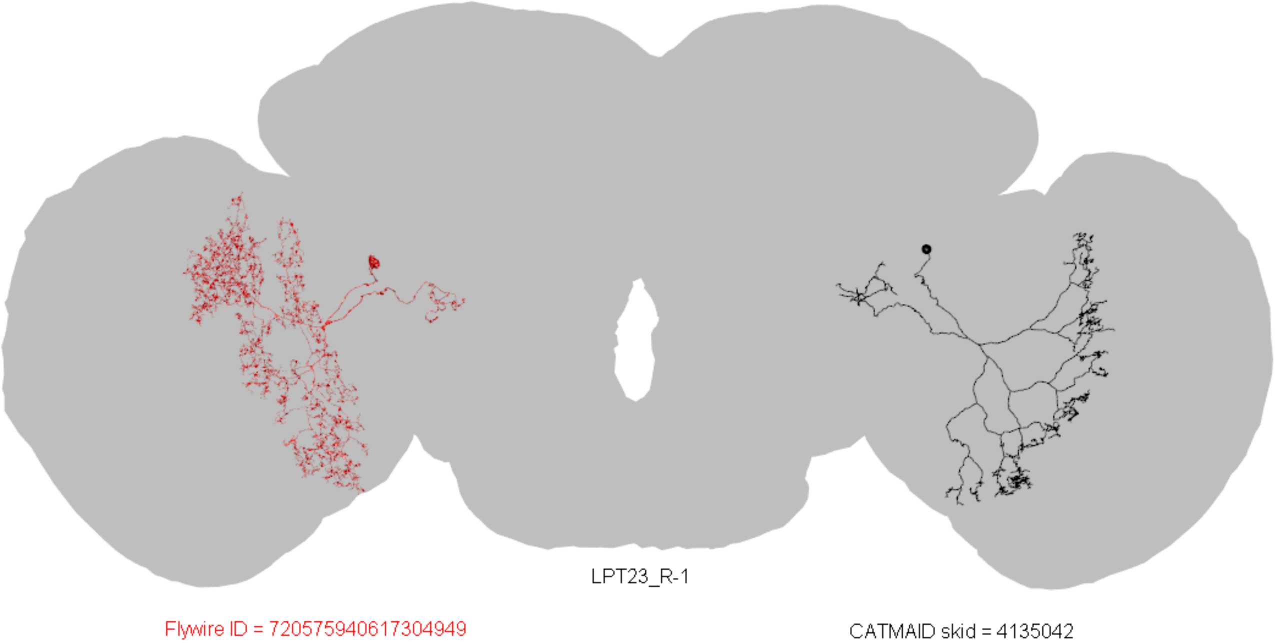
LPT23_R-1
Flywire ID = 720575940617304949
CATMAID skid = 4135042

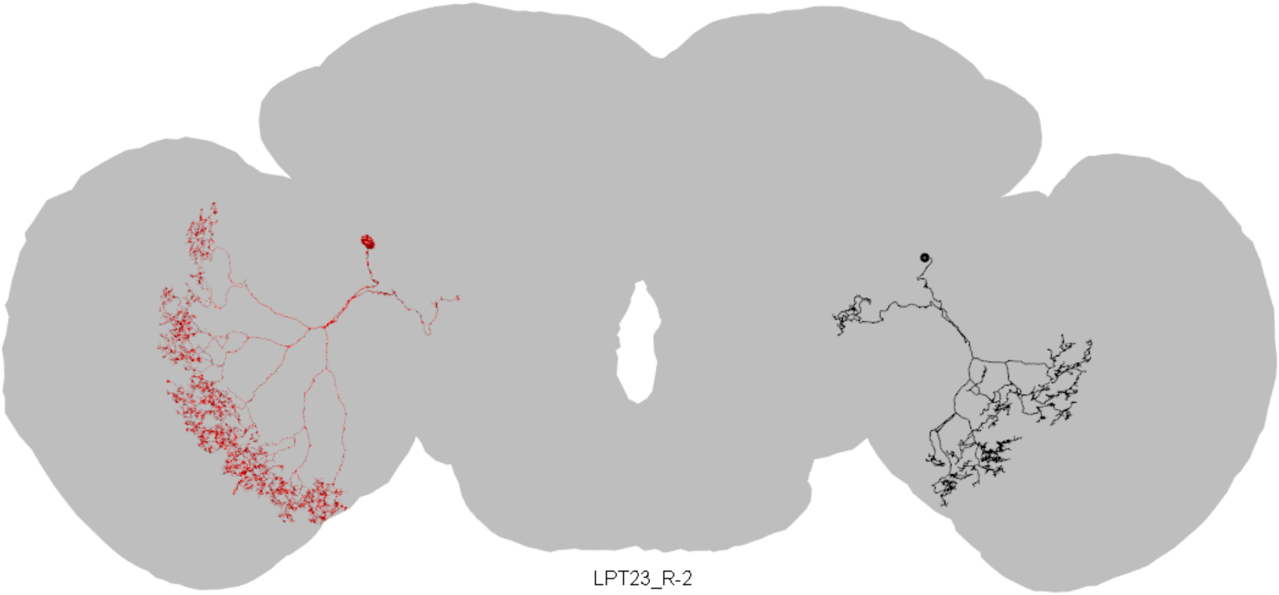

LPT23_R-2

Flywire ID = 720575940632545080

CATMAID skid = 4504548

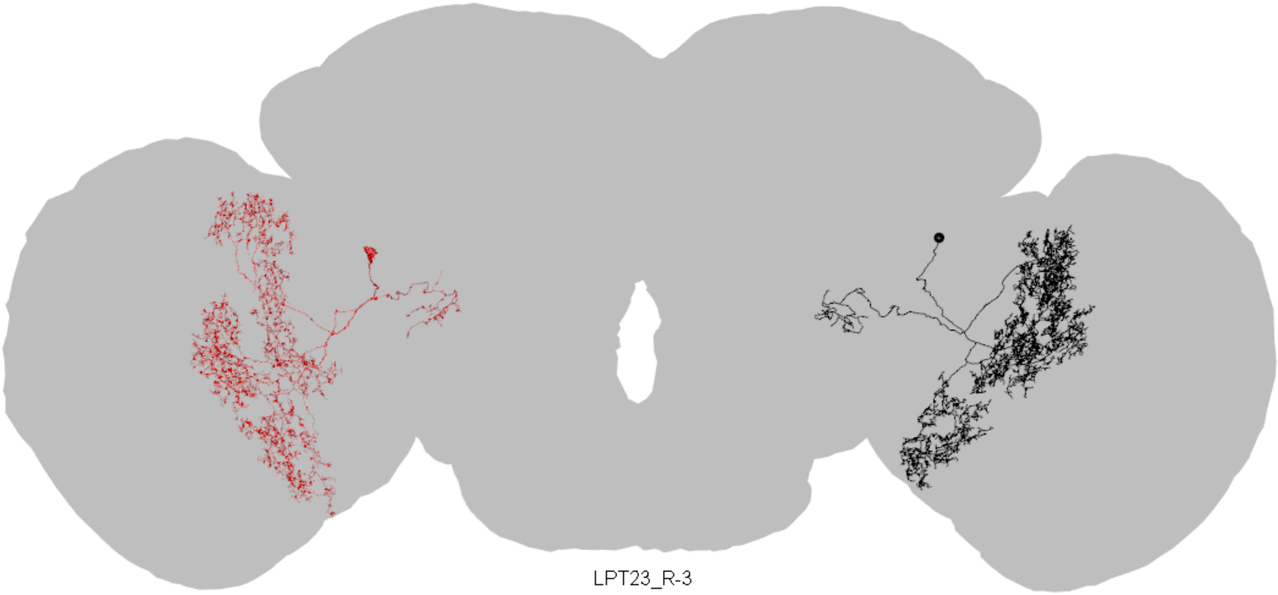

LPT23_R-3

Flywire ID = 720575940619638395

CATMAID skid = 4504557

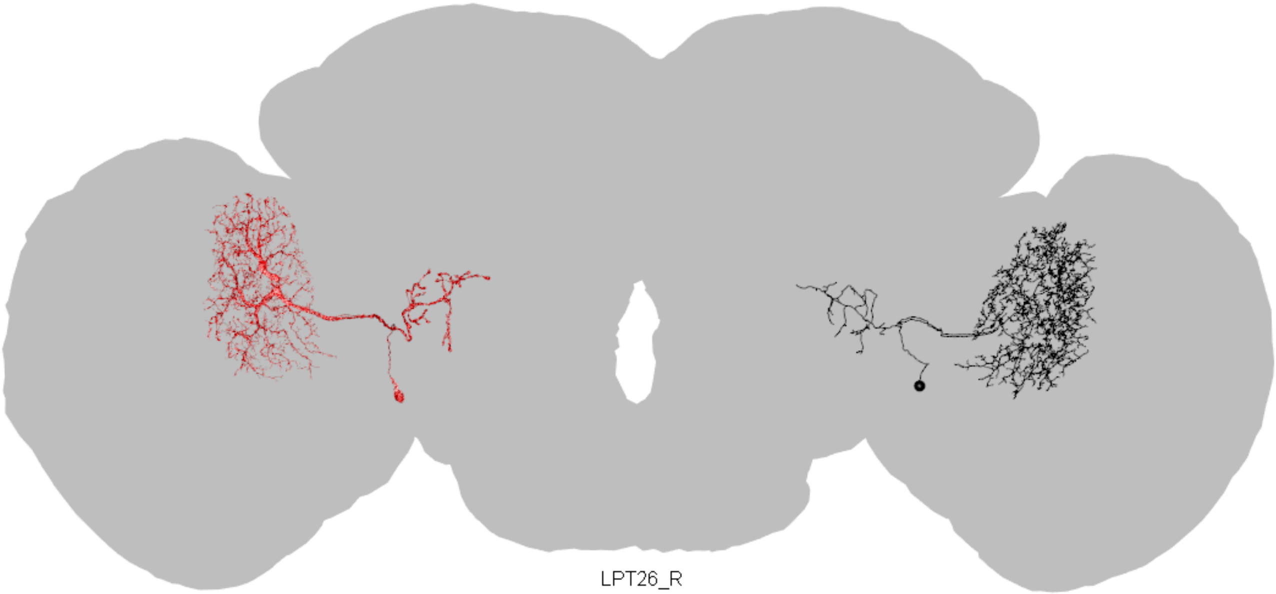

Flywire ID = 720575940630691895

CATMAID skid = 1107045

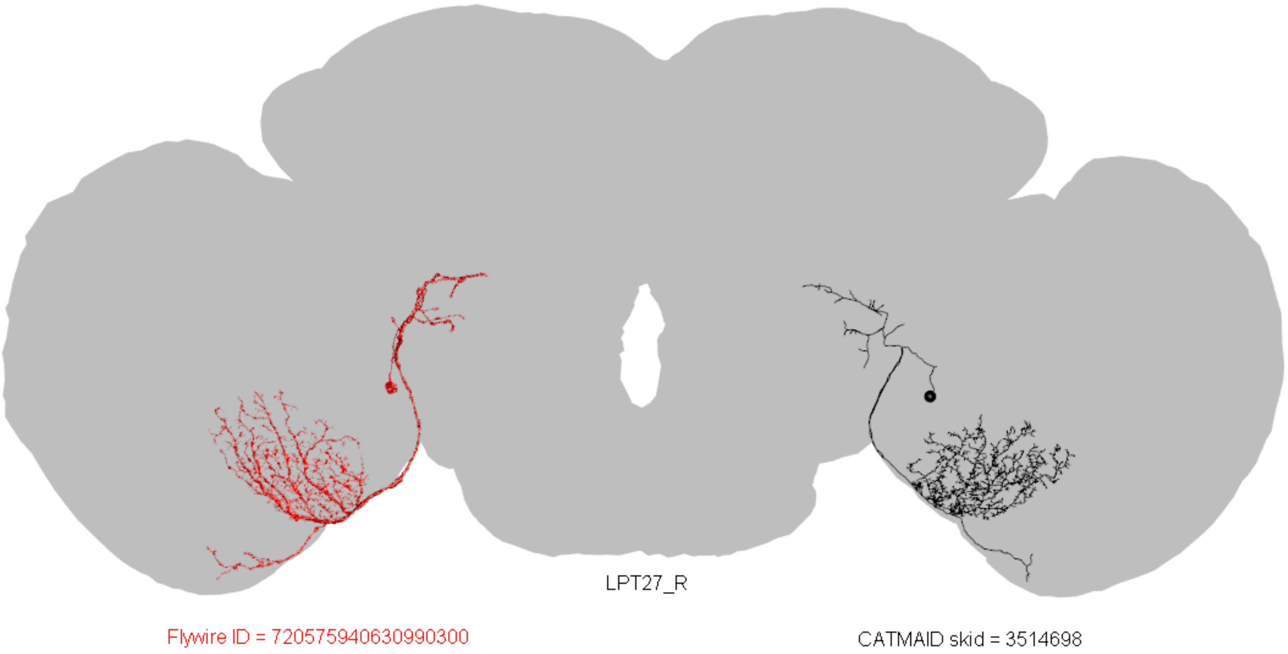
LPT27_R
Flywire ID = 720575940630990300
CATMAID skid = 3514698

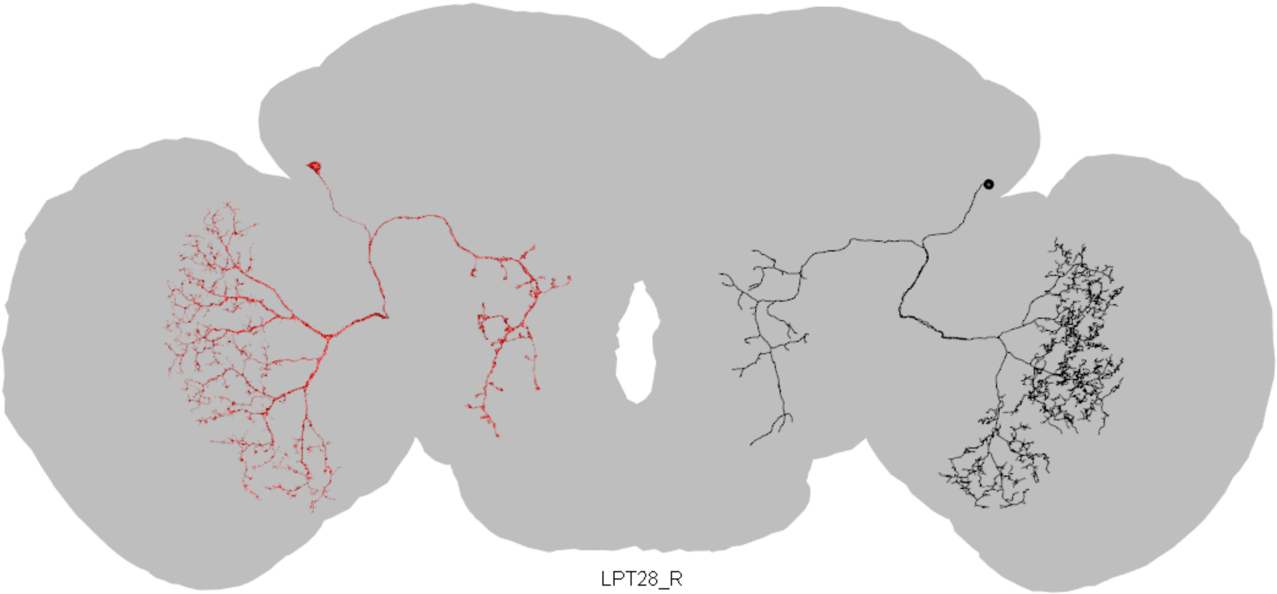

LPT28_R

Flywire ID = 720575940620898324

CATMAID skid = 1071860

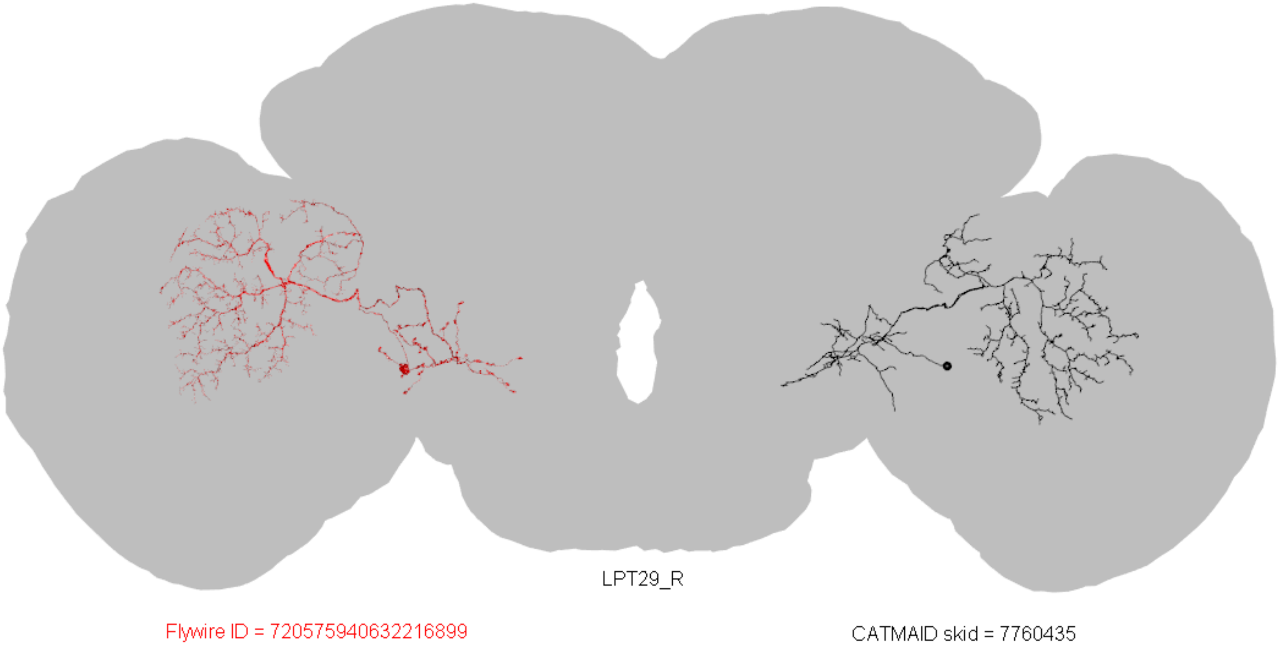

LPT29_R
Flywire ID = 720575940632216899
CATMAID skid = 7760435

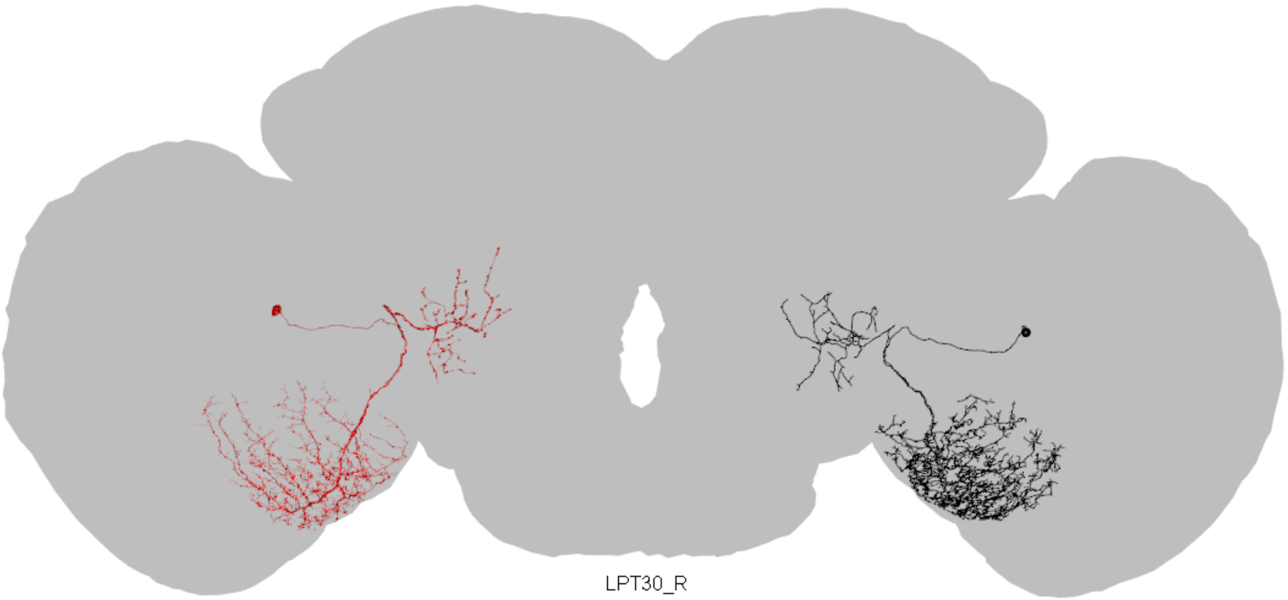

LPT30_R

Flywire ID = 720575940624047654

CATMAID skid = 7674107

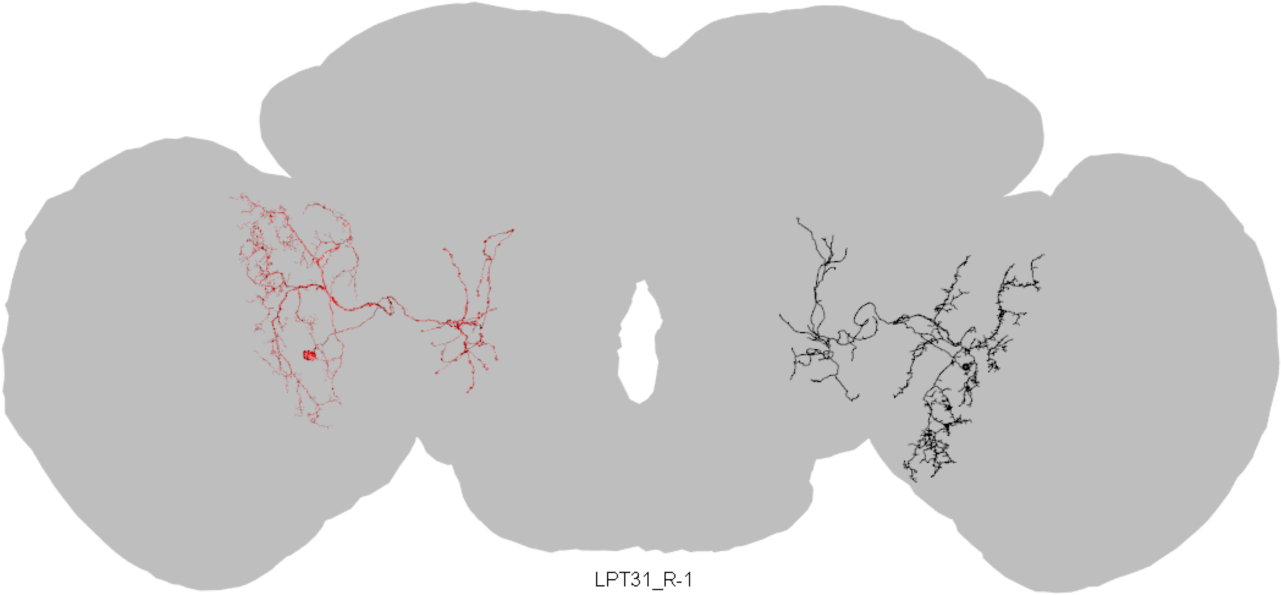

LPT31_R-1

Flywire ID = 720575940634166242

CATMAID skid = 7690273

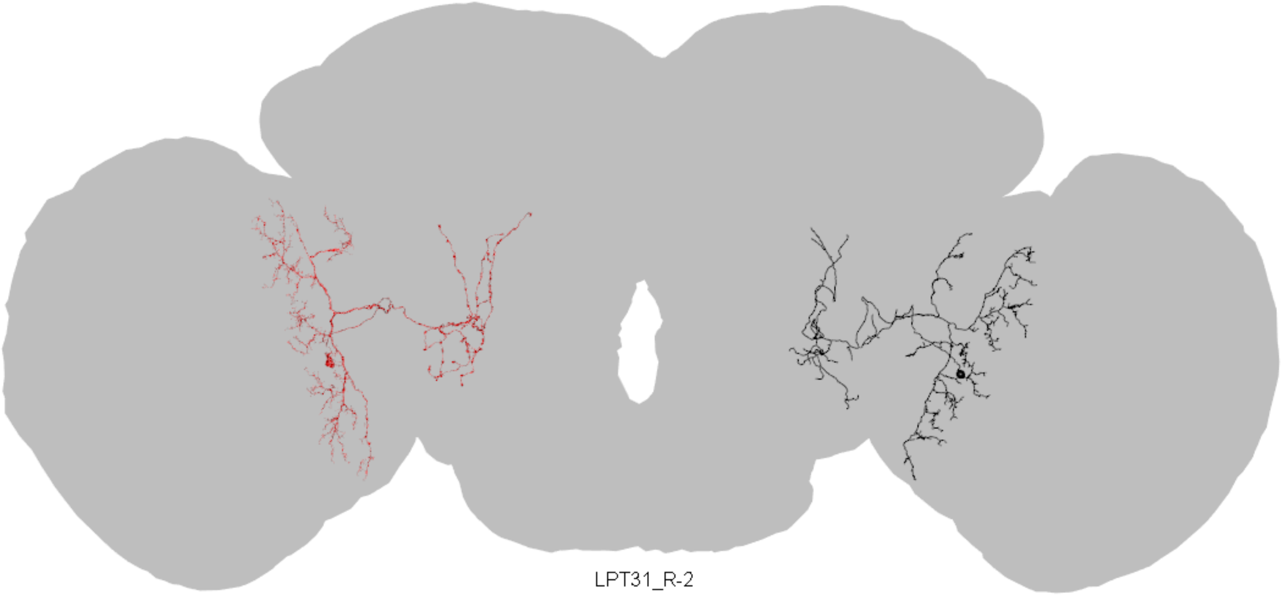

LPT31_R-2

Flywire ID = 720575940616260146

CATMAID skid = 7311408

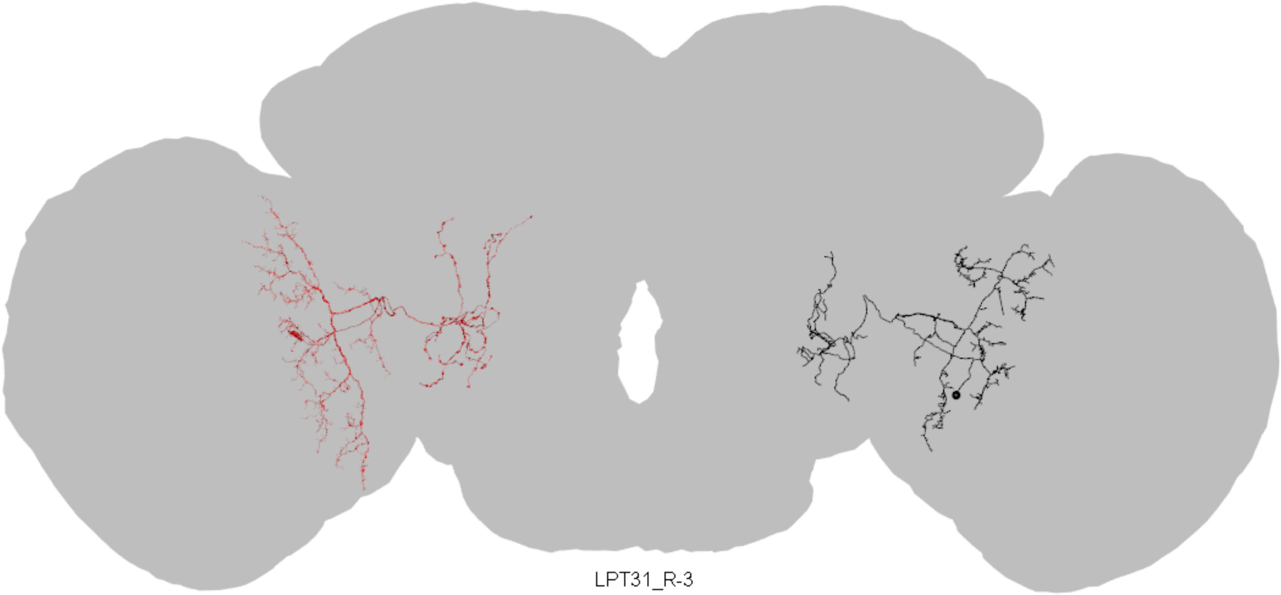

LPT31_R-3

Flywire ID = 720575940629577475

CATMAID skid = 7311493

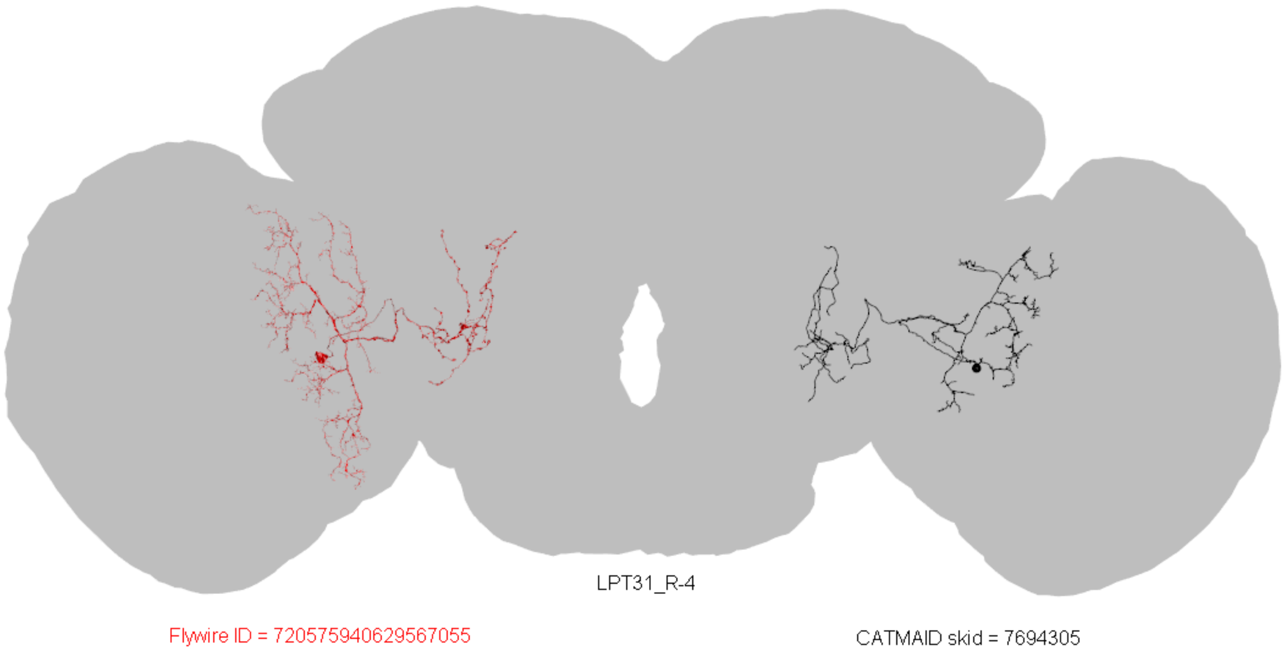
LPT31_R-4
Flywire ID = 720575940629567055
CATMAID skid = 7694305

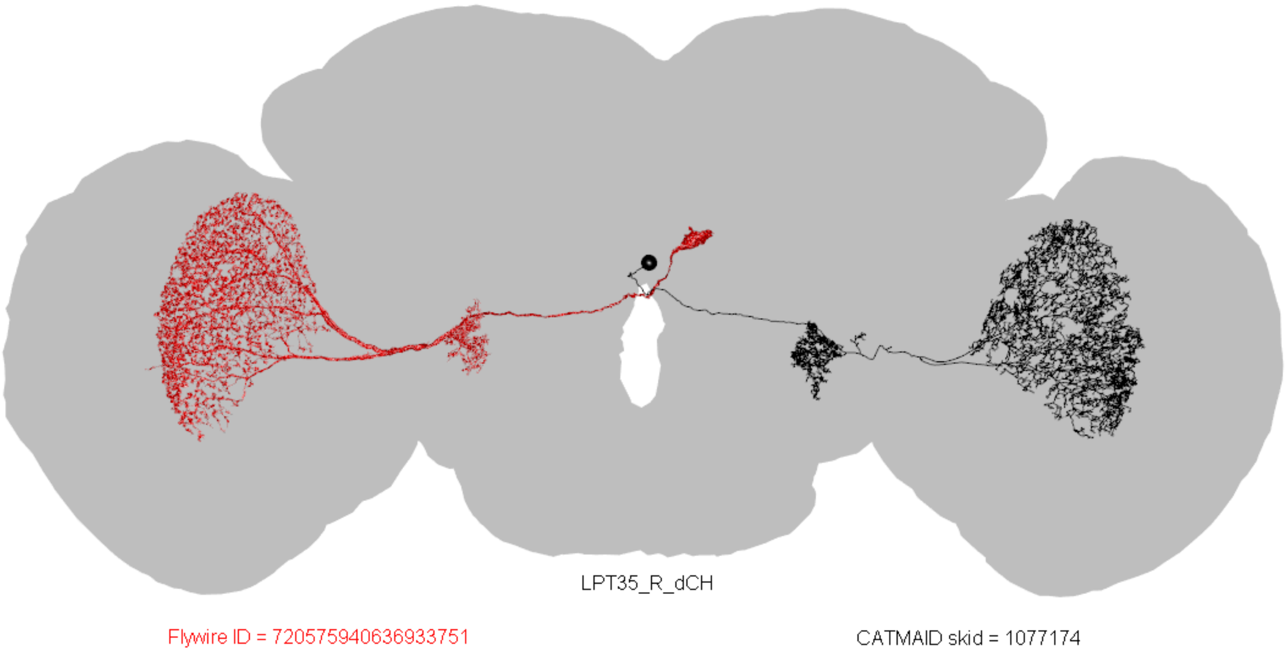

LPT35_R_dCH
Flywire ID = 720575940636933751
CATMAID skid = 1077174

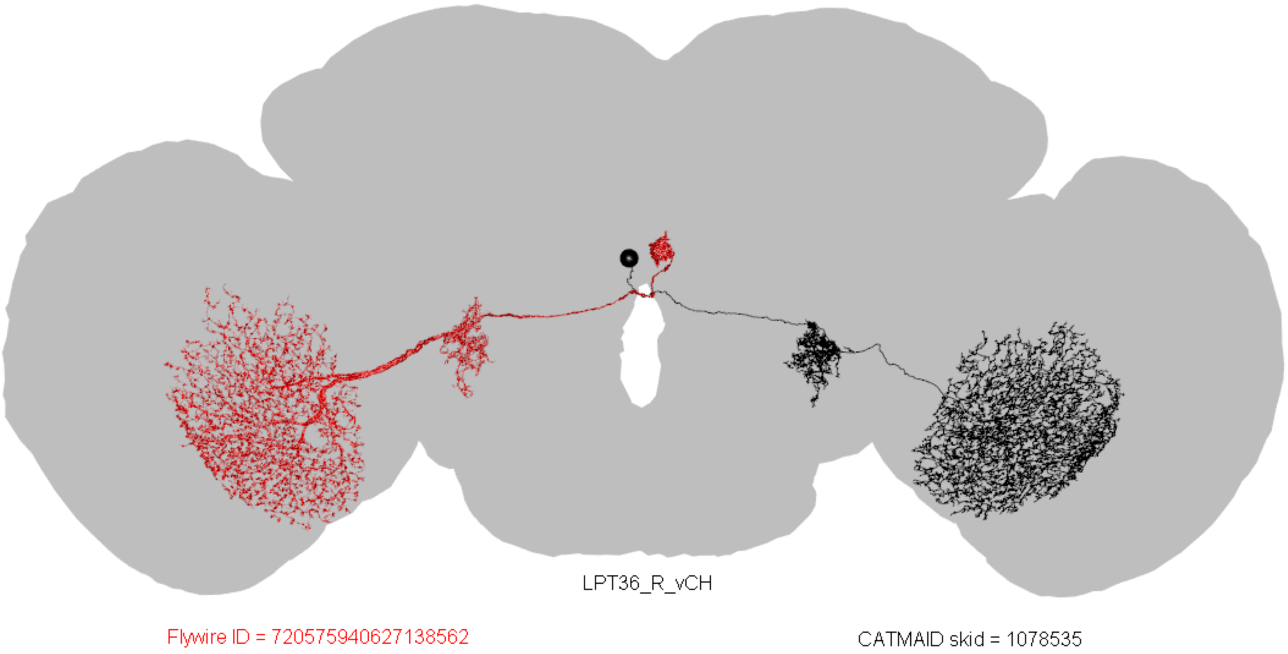

LPT36_R_vCH
Flywire ID = 720575940627138562
CATMAID skid = 1078535

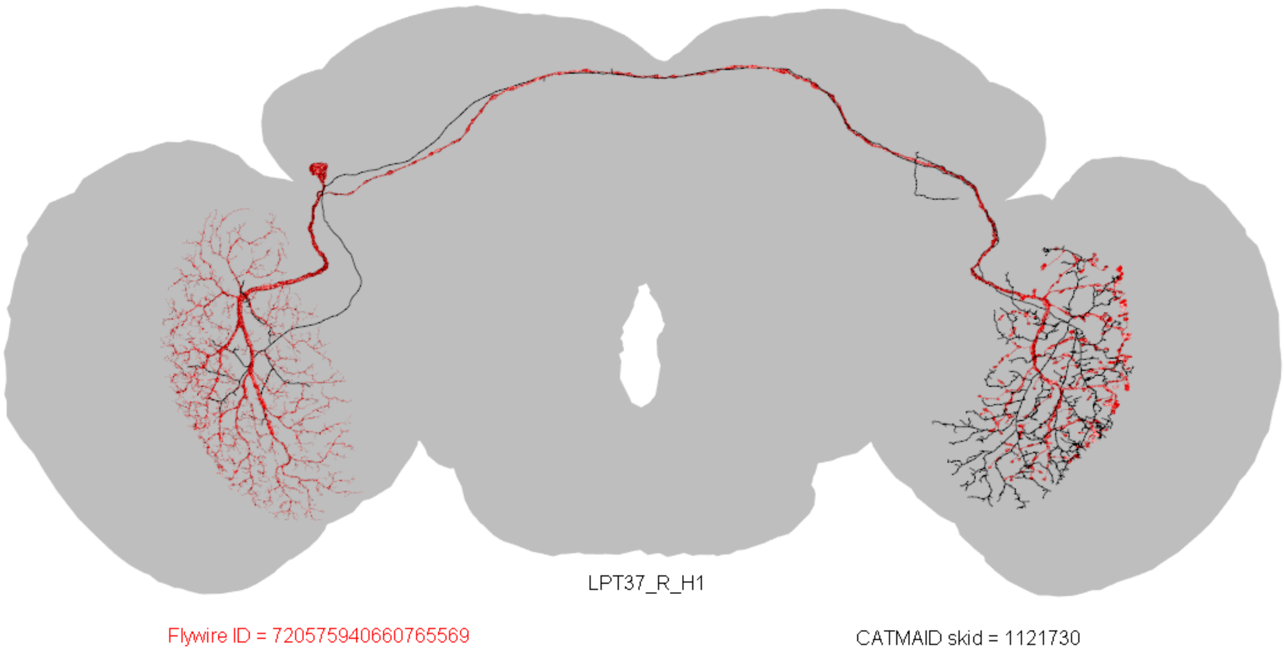
LPT37_R_H1
Flywire ID = 720575940660765569
CATMAID skid = 1121730

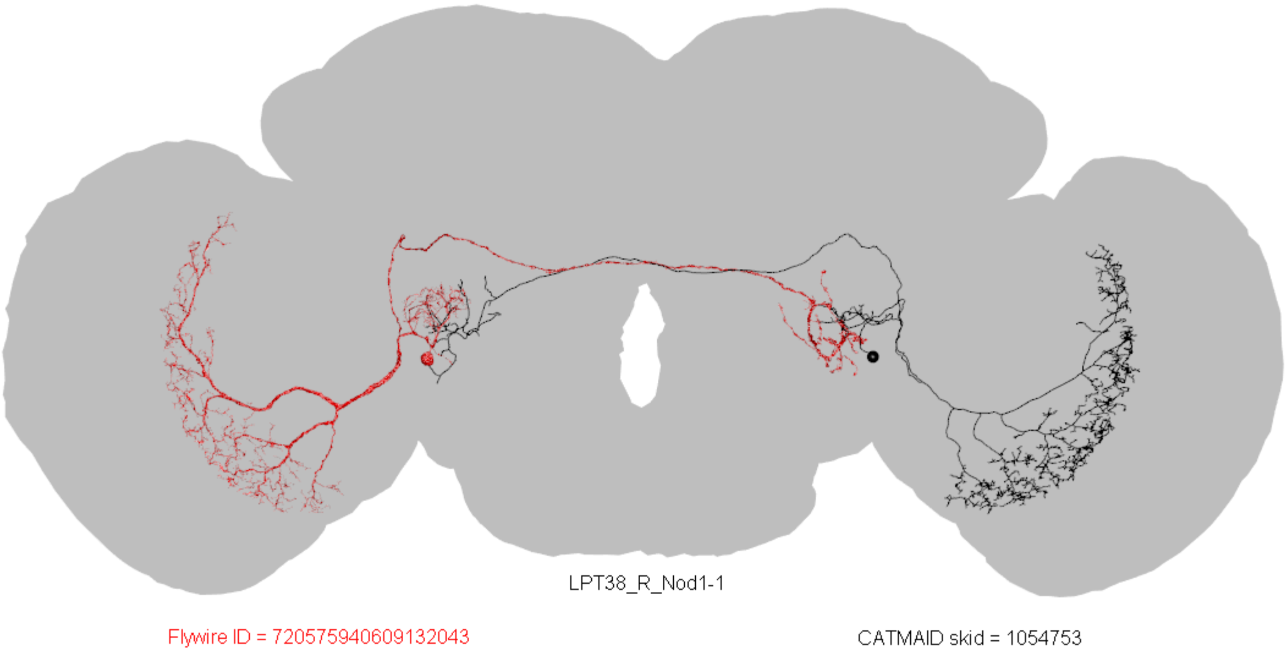
LPT38_R_Nod1-1
Flywire ID = 720575940609132043
CATMAID skid = 1054753

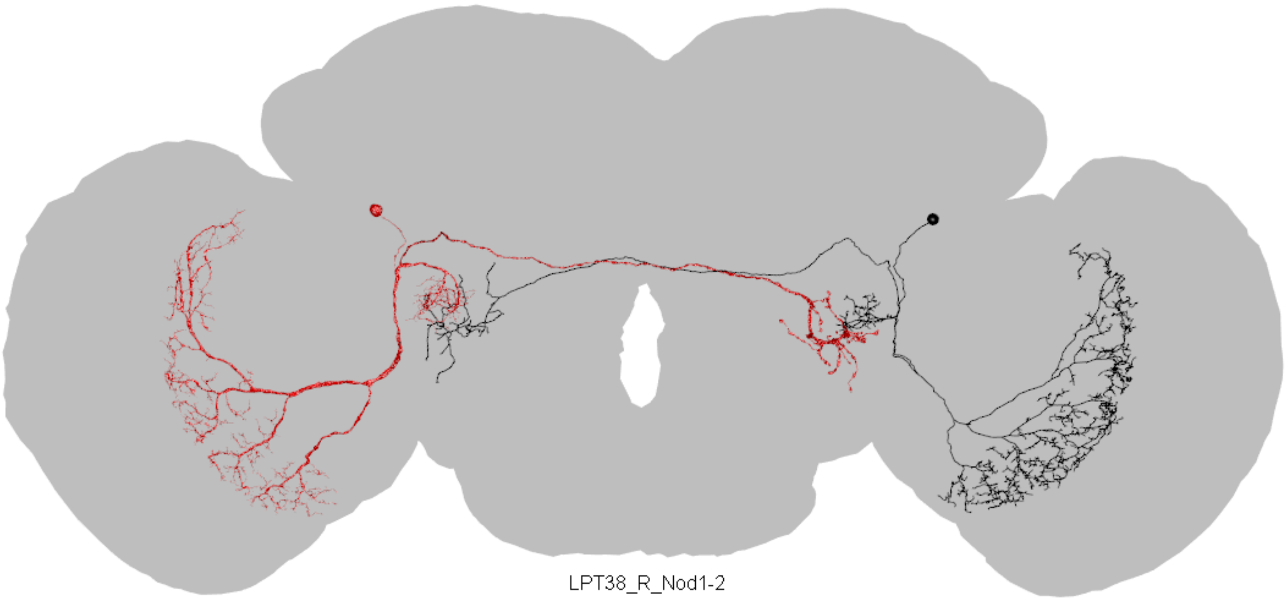

Flywire ID = 720575940628438427

CATMAID skid = 3546483

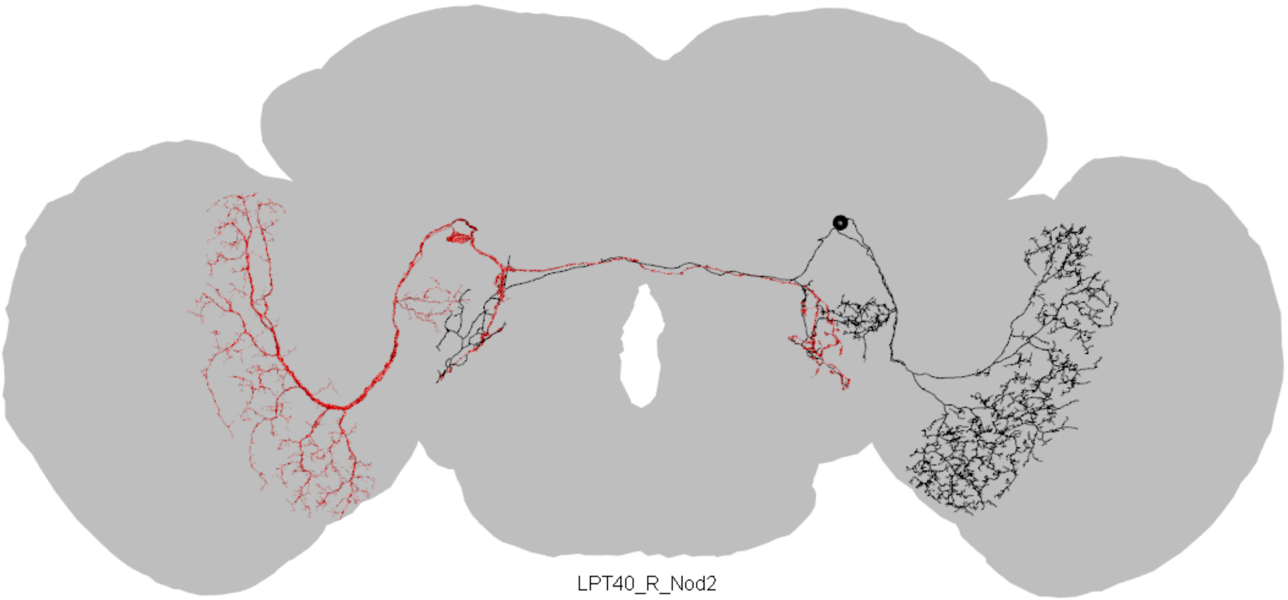

LPT40_R_Nod2

Flywire ID = 720575940623235683

CATMAID skid = 902072

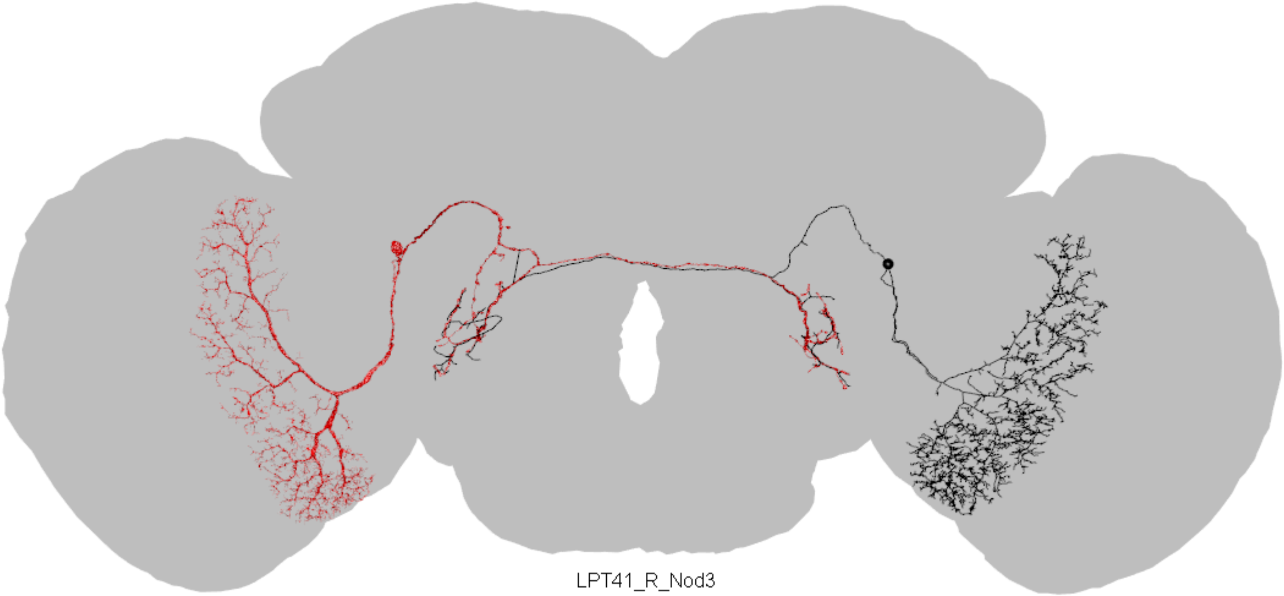

LPT41_R_Nod3

Flywire ID = 720575940623384781

CATMAID skid = 1058996

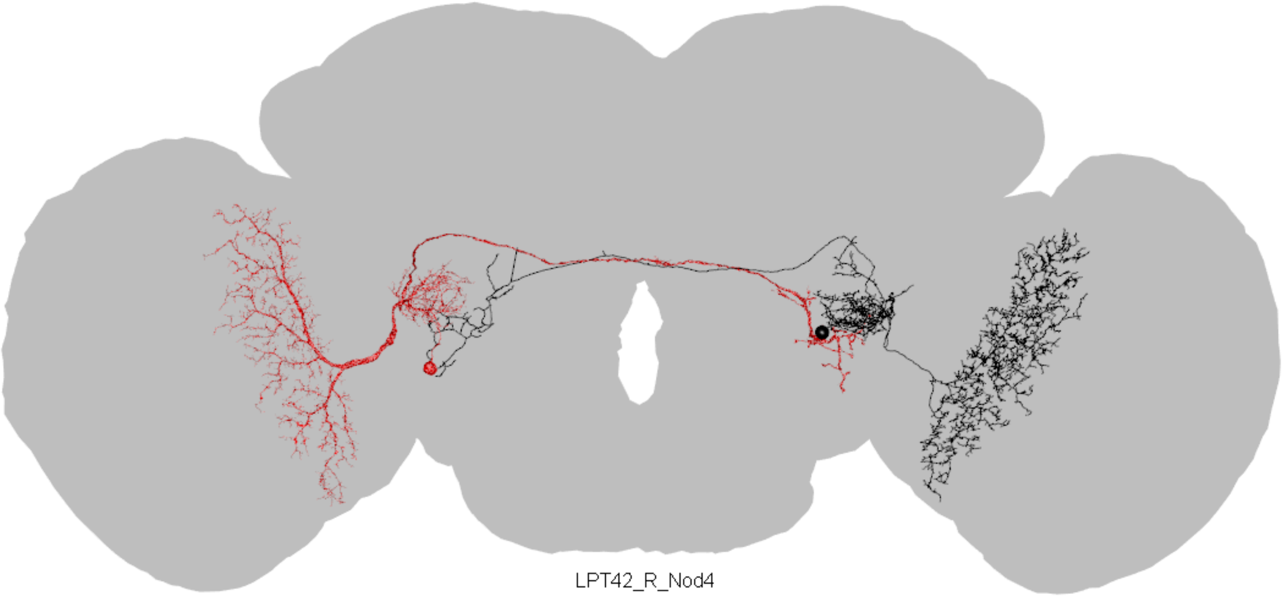

LPT42_R_Nod4

Flywire ID = 720575940625992781

CATMAID skid = 1106958

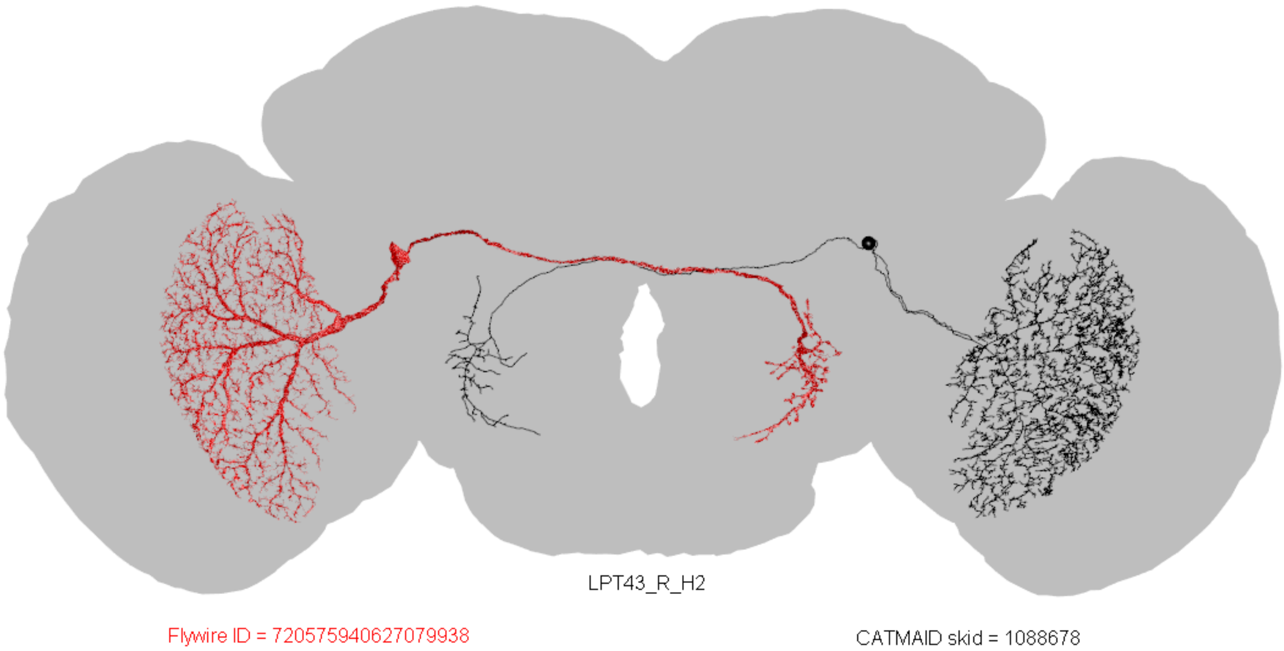
LPT43_R_H2
Flywire ID = 720575940627079938
CATMAID skid = 1088678

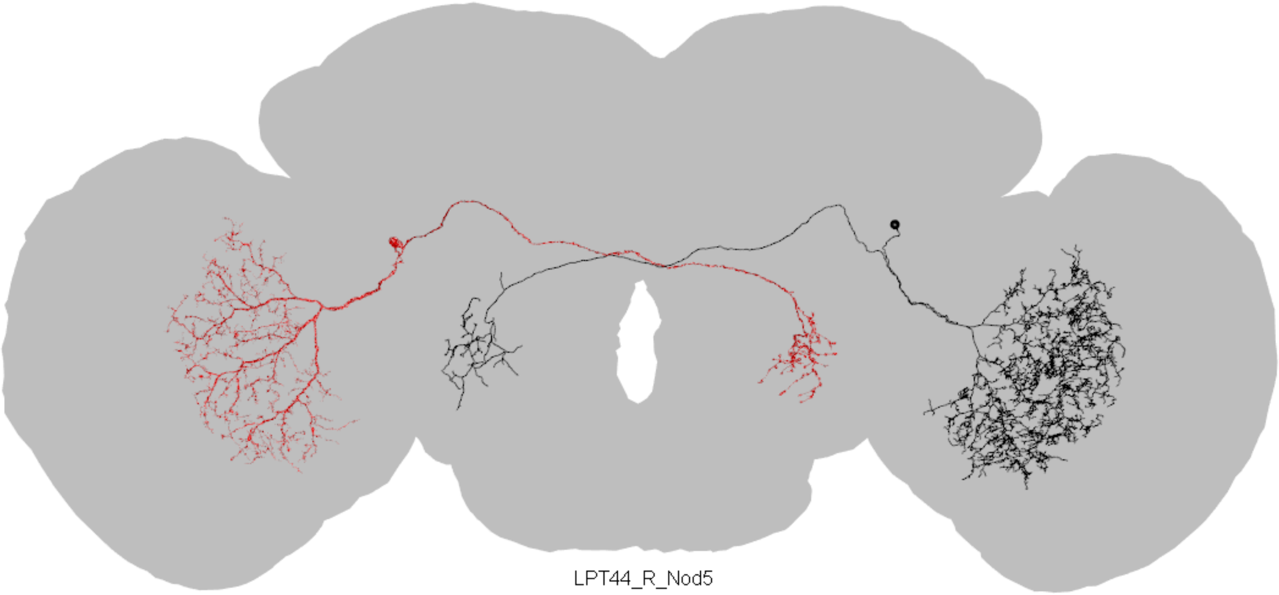
LPT44_R_Nod5

Flywire ID = 720575940633685459

CATMAID skid = 1121795

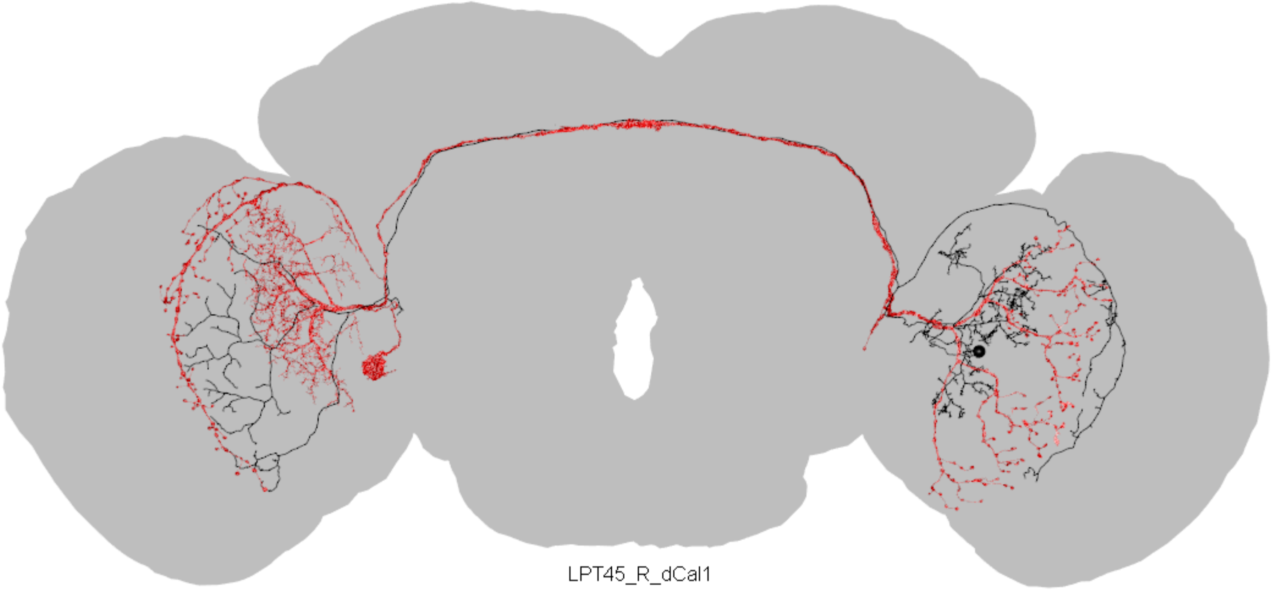

LPT45_R_dCal1

Flywire ID = 720575940628350997

CATMAID skid = 7204844

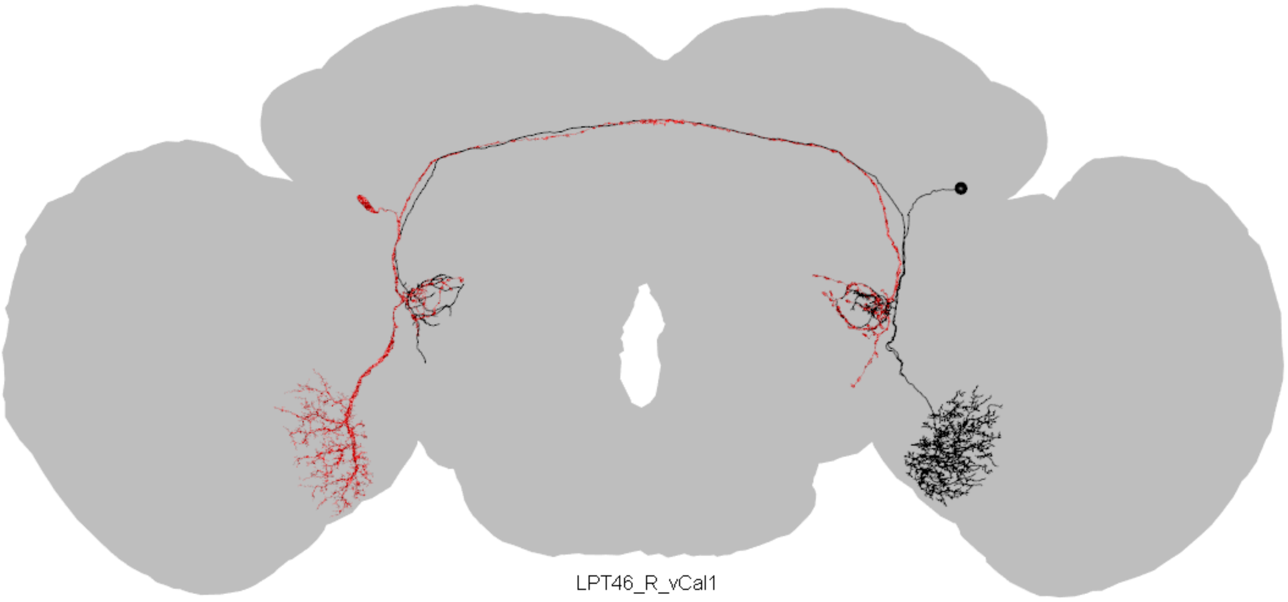

LPT46_R_vCal1

Flywire ID = 720575940641223888

CATMAID skid = 7510076

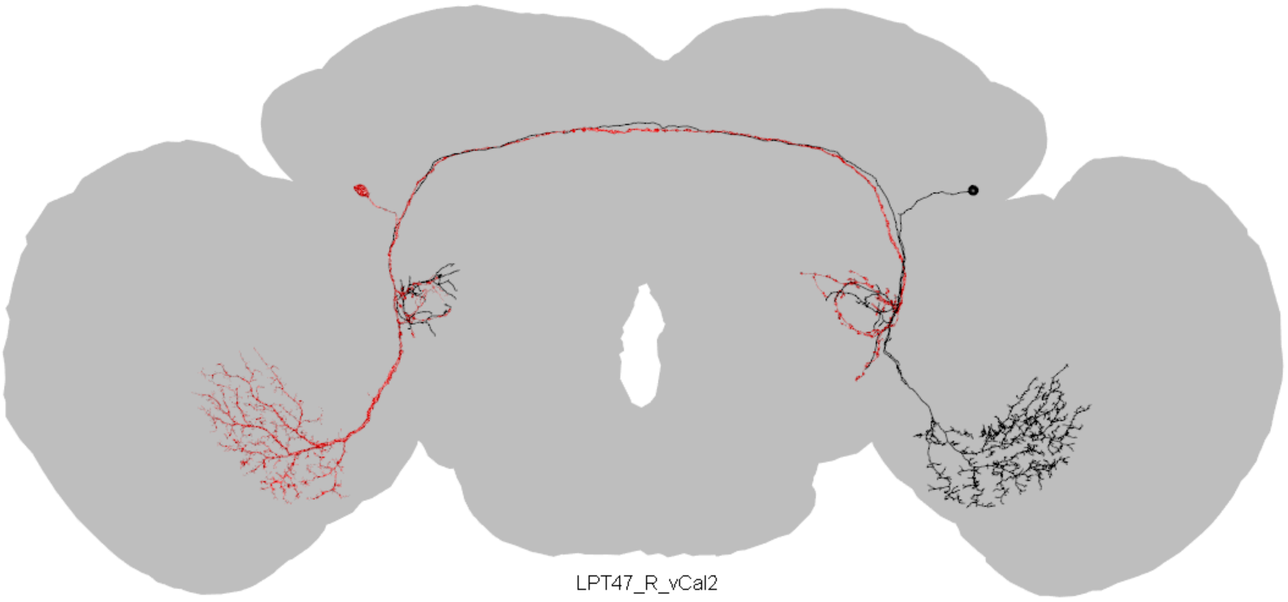

LPT47_R_vCal2

Flywire ID = 720575940618653524

CATMAID skid = 3529071

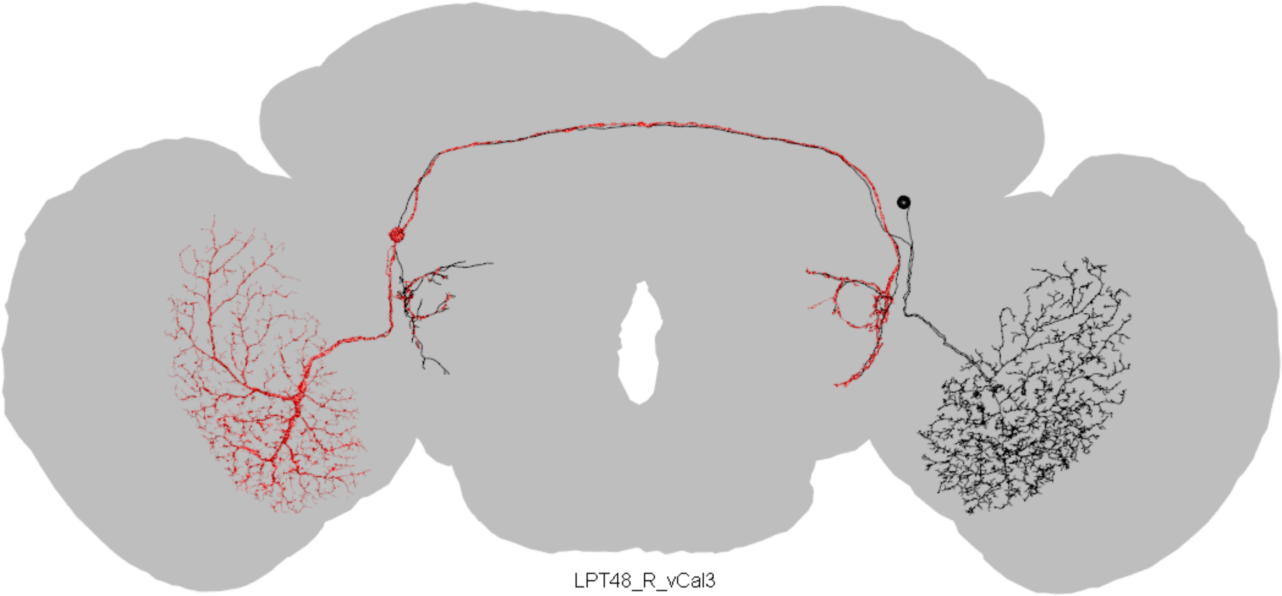

LPT48_R_vCal3

Flywire ID = 720575940626919780

CATMAID skid = 1056097

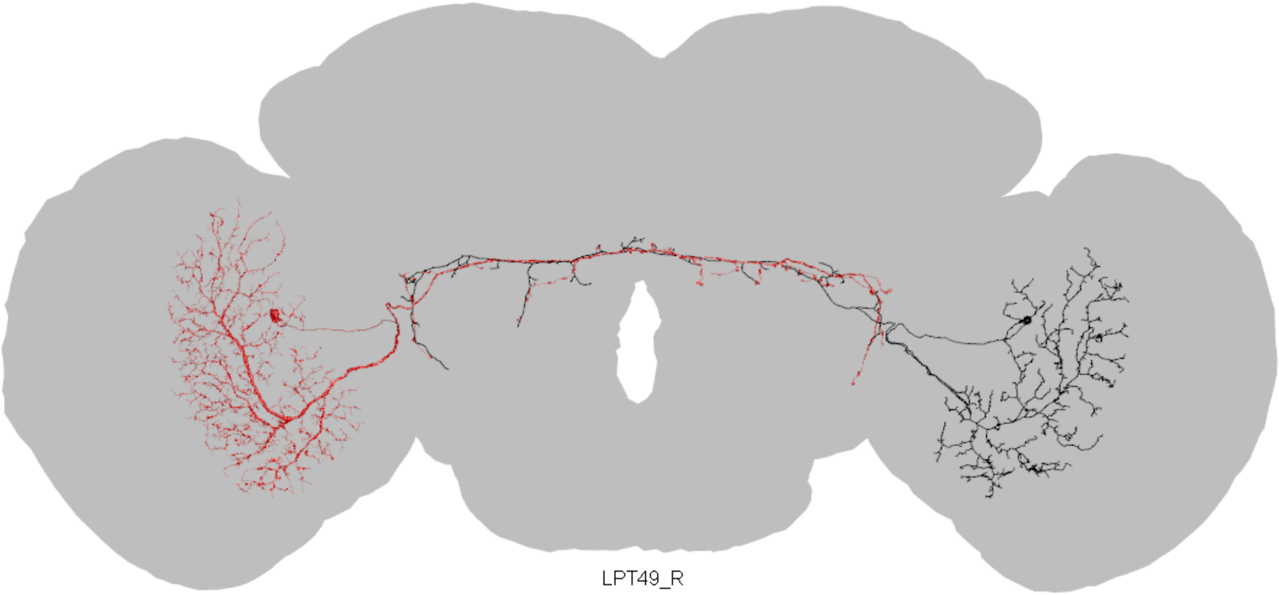

LPT49_R

Flywire ID = 720575940638155998

CATMAID skid = 1110693

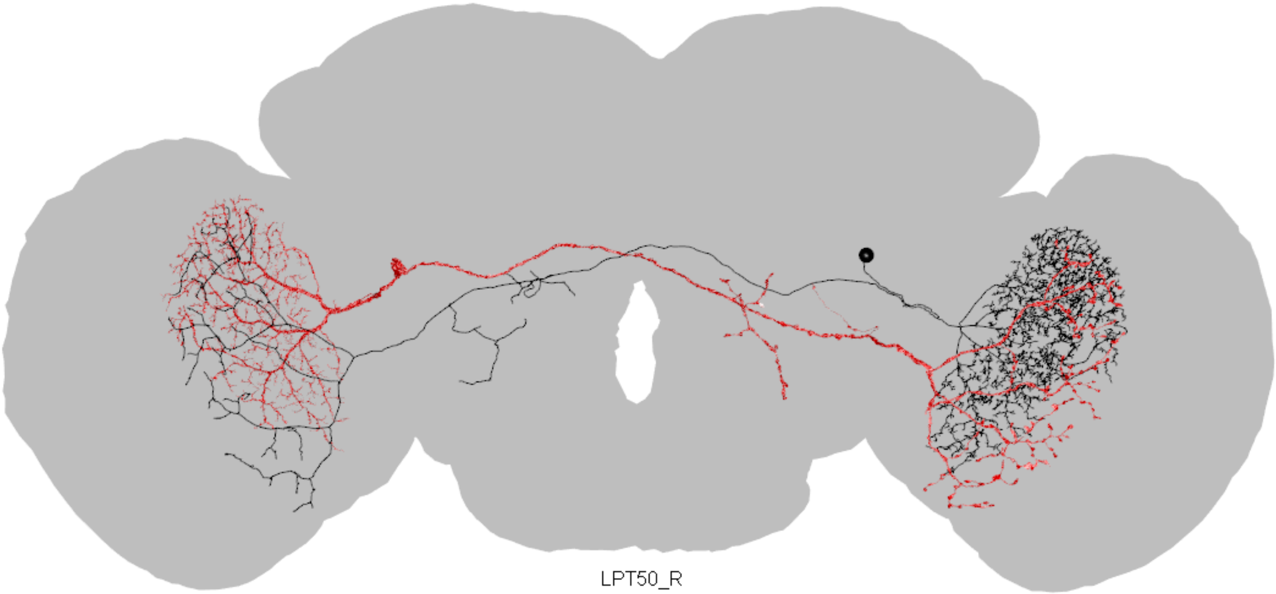

Flywire ID = 720575940655599777

CATMAID skid = 905761

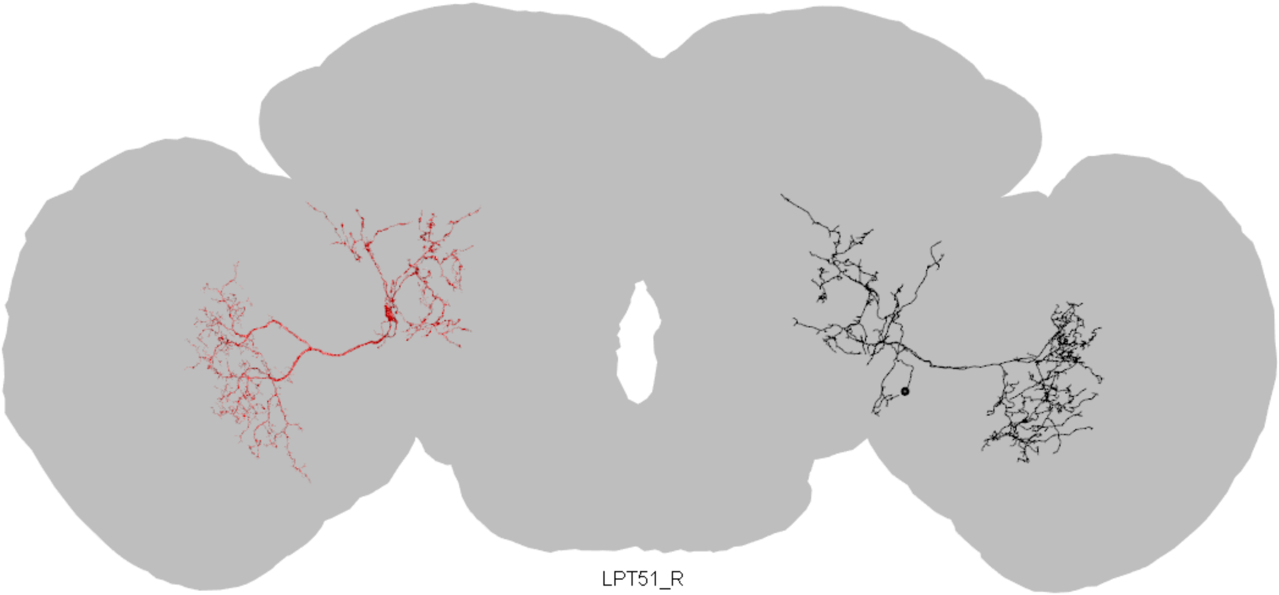

LPT51_R

Flywire ID = 720575940608287701

CATMAID skid = 4224711

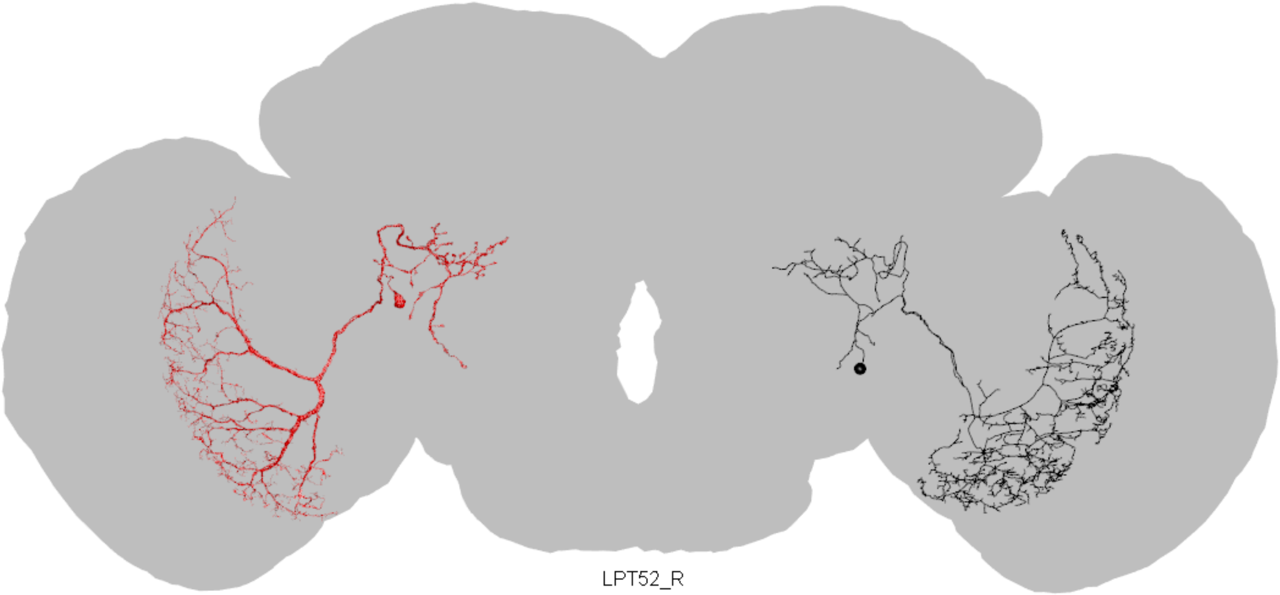

LPT52_R

Flywire ID = 720575940640716928

CATMAID skid = 1107296

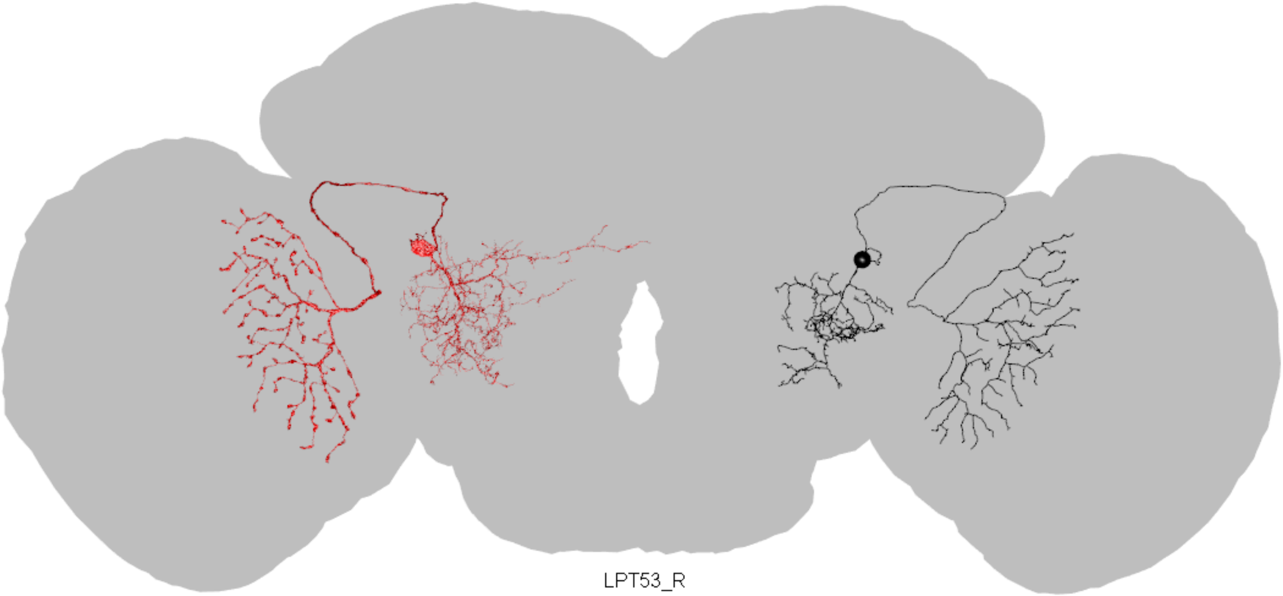

LPT53_R

Flywire ID = 720575940653093110

CATMAID skid = 1111992

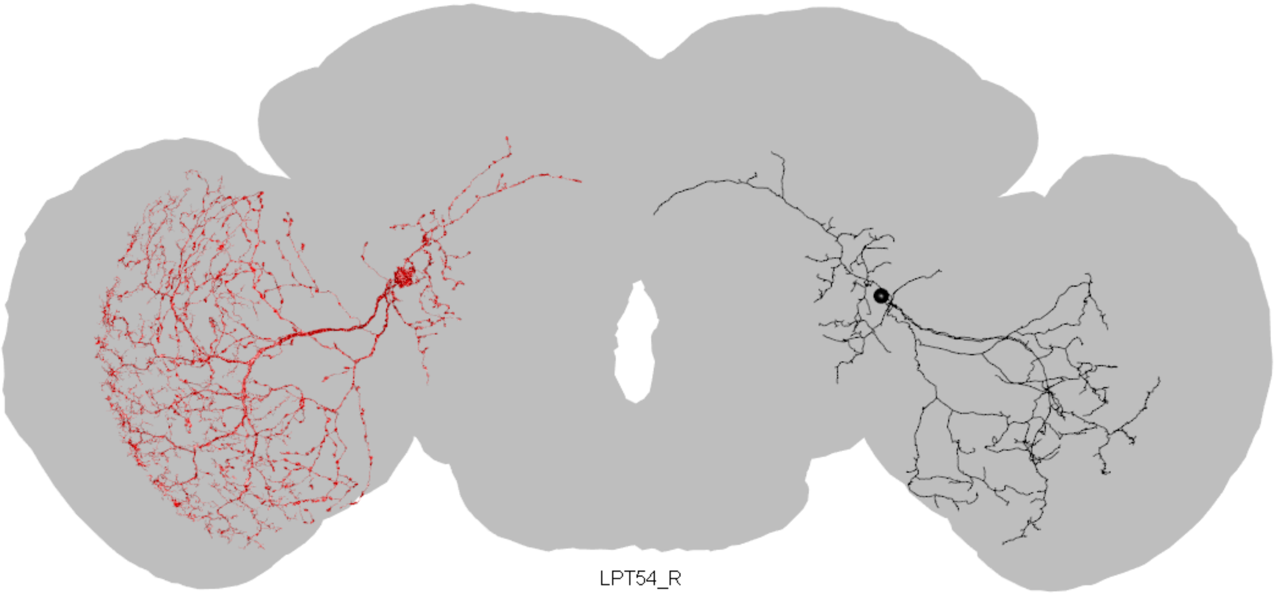

LPT54_R

Flywire ID = 720575940655602849

CATMAID skid = 4235388

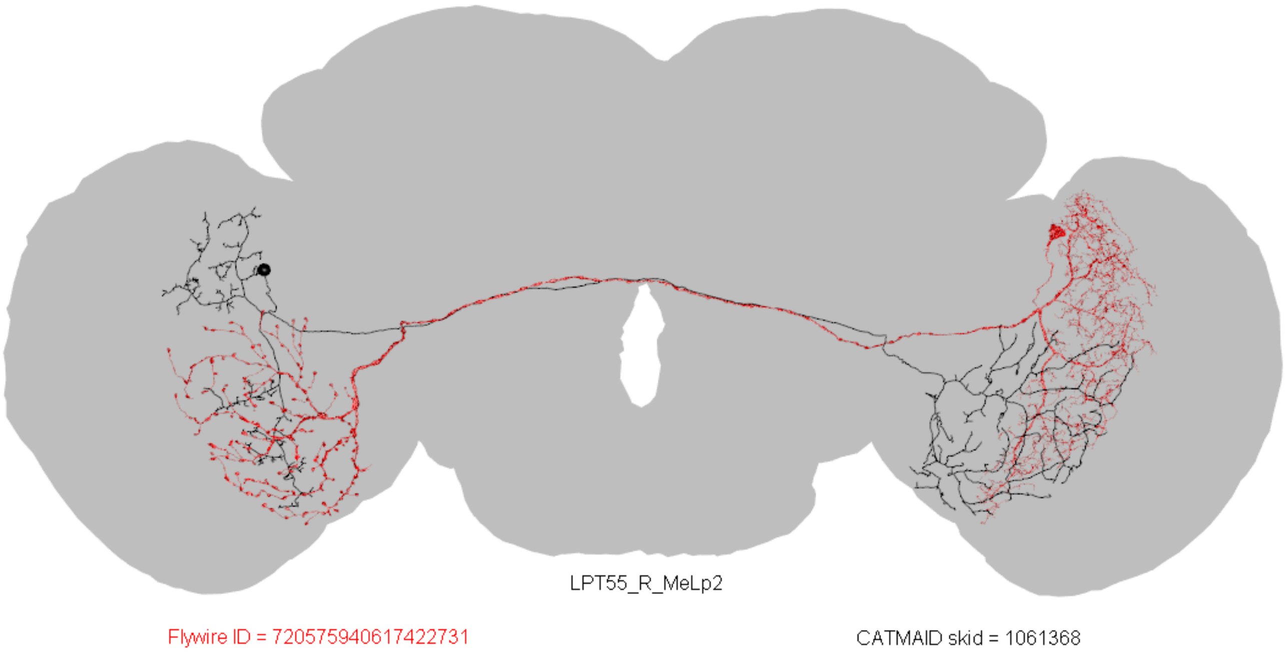

LPT55_R_MeLp2
Flywire ID = 720575940617422731
CATMAID skid = 1061368

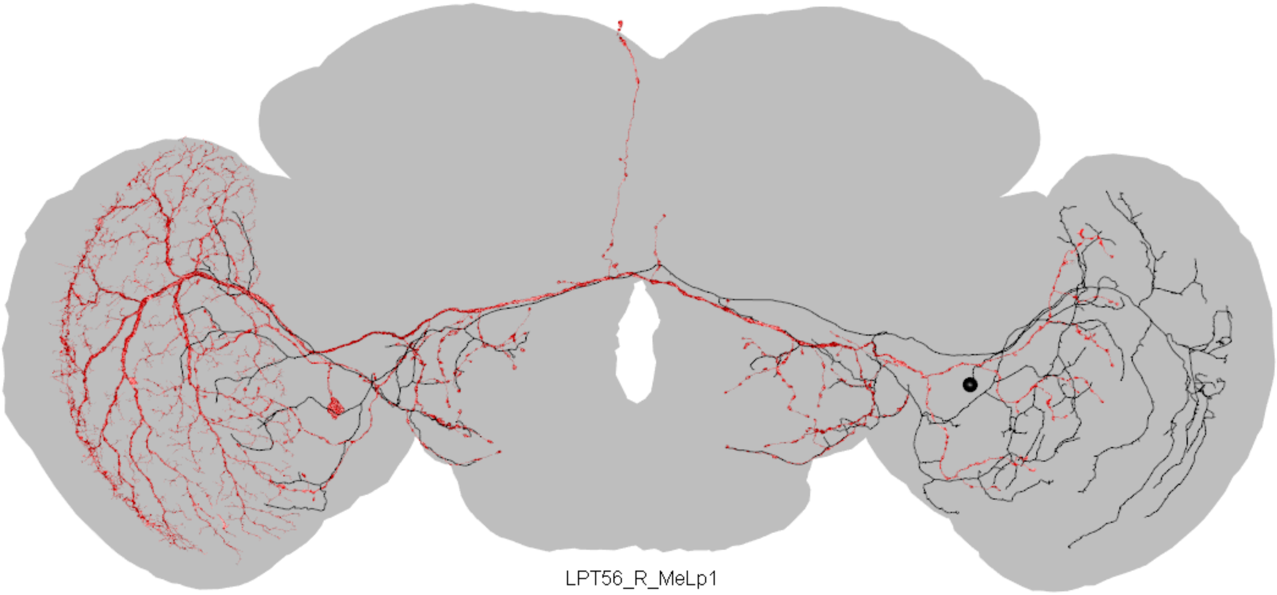

LPT56_R_MeLp1

Flywire ID = 720575940655602849

CATMAID skid = 3509520

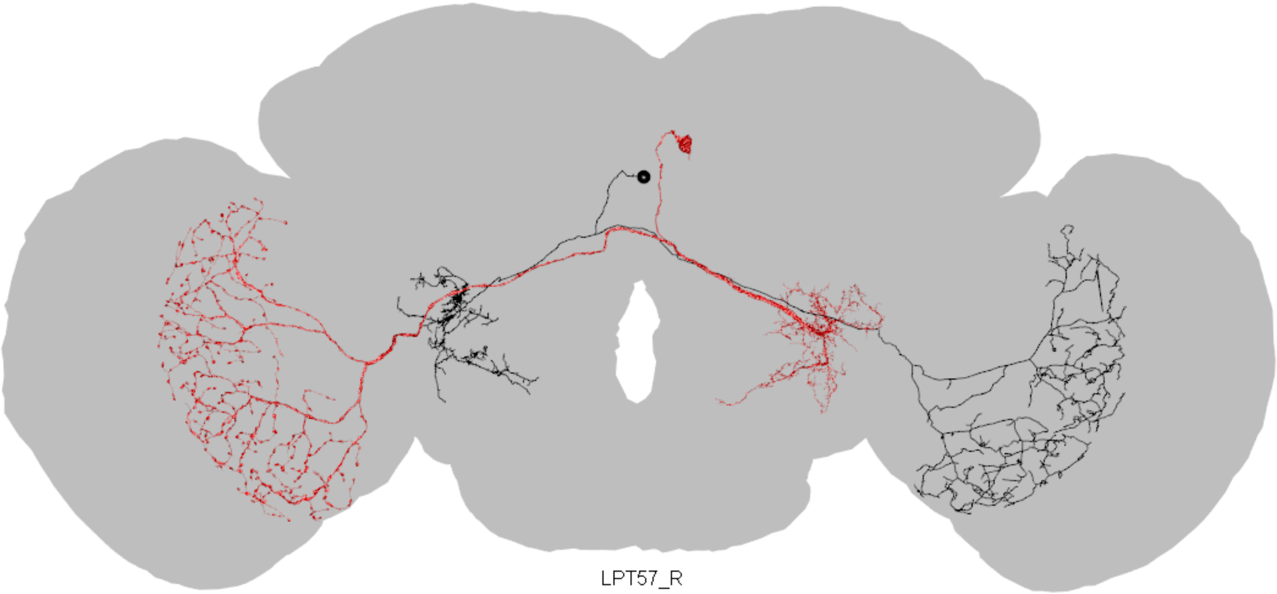

LPT57_R

Flywire ID = 720575940632041746

CATMAID skid = 886797

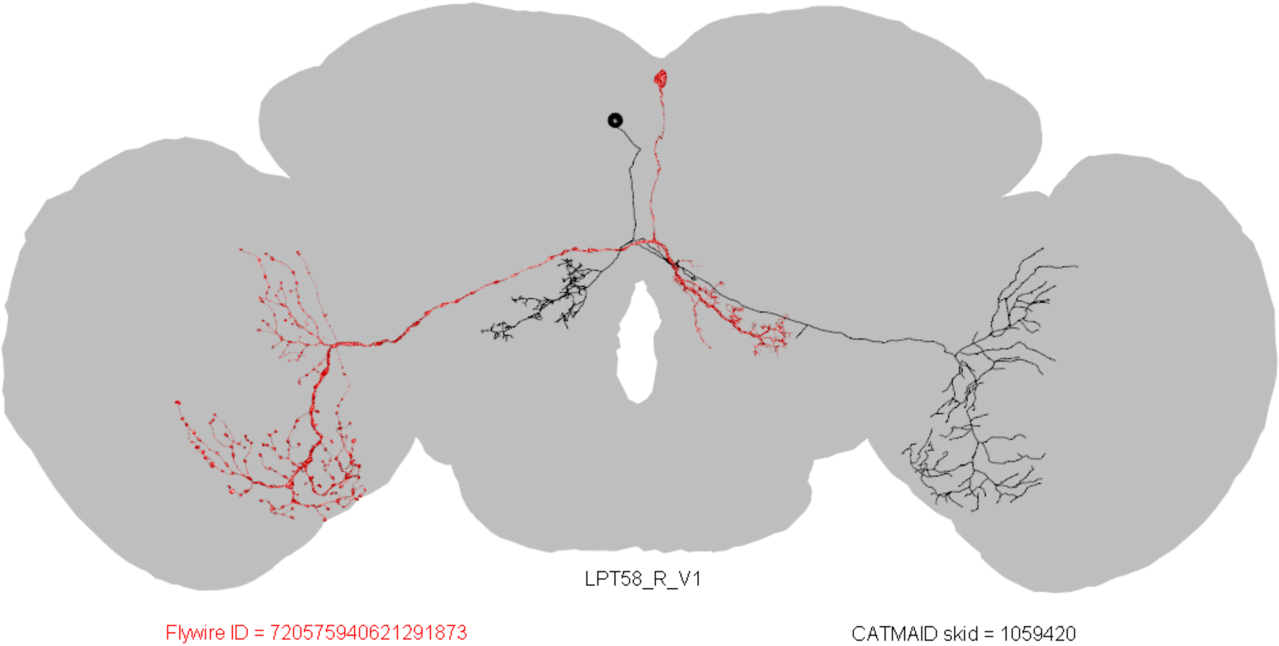
LPT58_R_V1
Flywire ID = 720575940621291873
CATMAID skid = 1059420
