# Supplementary material for: A comprehensive neuroanatomical survey of the Drosophila Lobula Plate Tangential Neurons with predictions for their optic flow sensitivity
Source: bioRxiv. 2023 Oct 17:2023.10.16.562634. Preprint. [Version 1] doi: 10.1101/2023.10.16.562634 (PMC10614863; doi:10.1101/2023.10.16.562634)
Supplement: Supplement 4 — Supplementary File 4: Gallery of LPT neurons overlayed with putative Hemibrain matches. The LPT neurons reconstructed in FAFB are shown in red, and the matched Hemibrain neurons are shown in black (56 right side matches, note that LPT51 is matched to 2 Hemibrain neurons, and 17 left side matches). [file media-4.pdf]

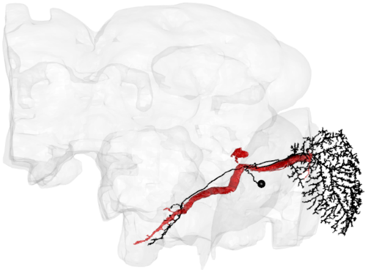

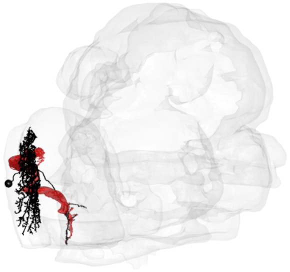

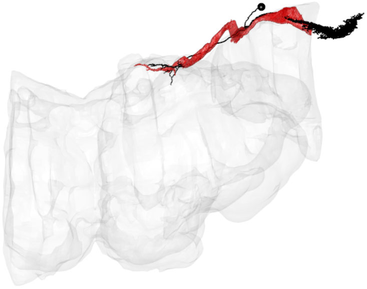

LPT01_R_HSN

CATMAID skid = 830793

Hemibrain bodyid= 2211443902

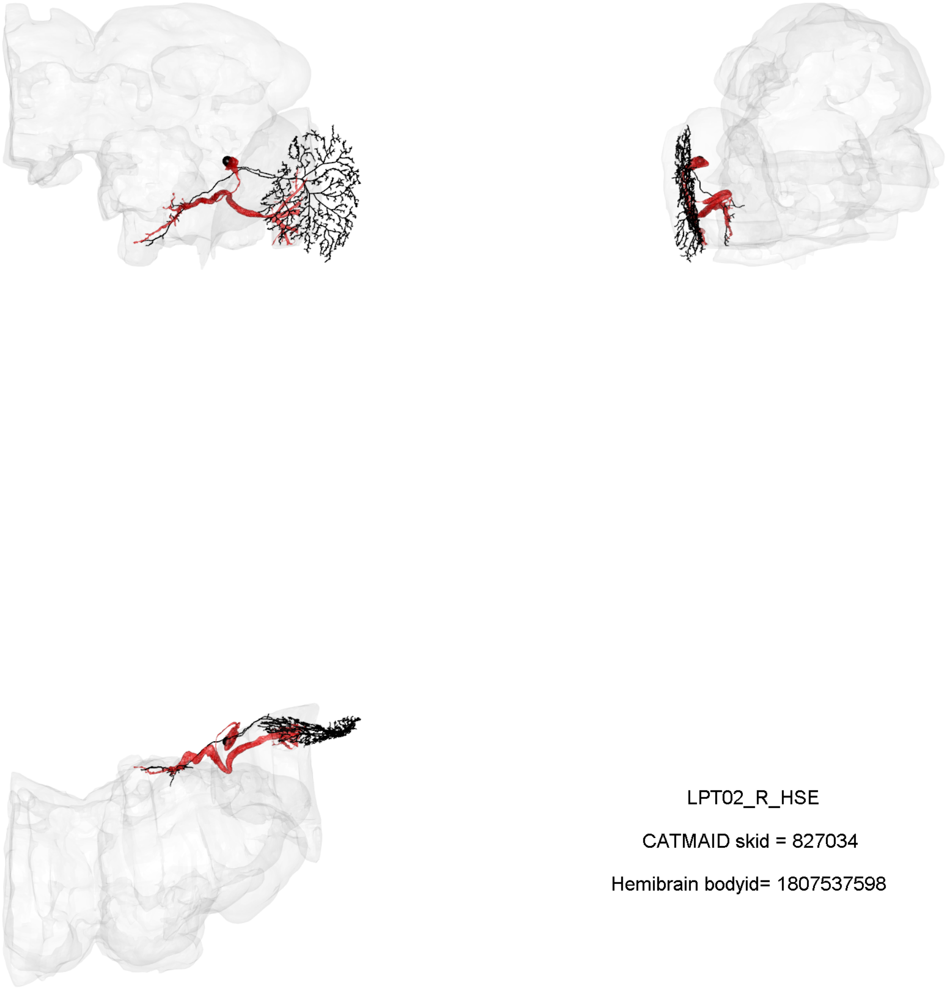

LPT02_R_HSE

CATMAID skid = 827034

Hemibrain bodyid= 1807537598

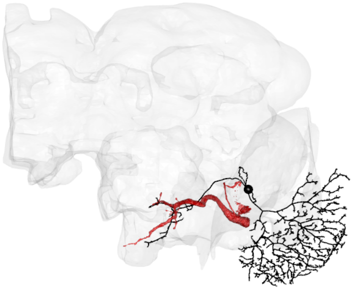

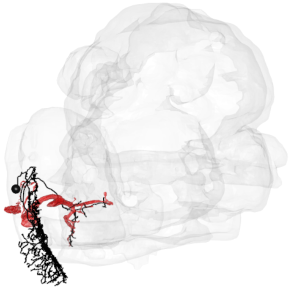

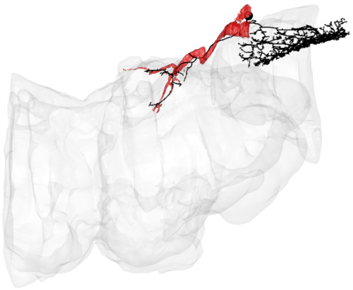

LPT03_R_HSS

CATMAID skid = 4058824

Hemibrain bodyid= 2179731270

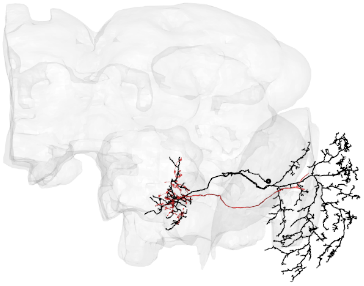

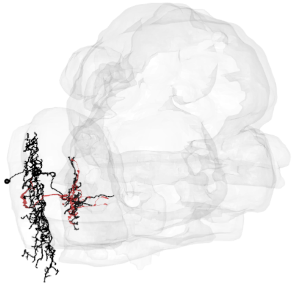

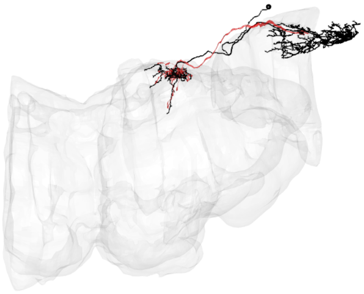

LPT04_R_HST

CATMAID skid = 985774

Hemibrain bodyid= 5813042688

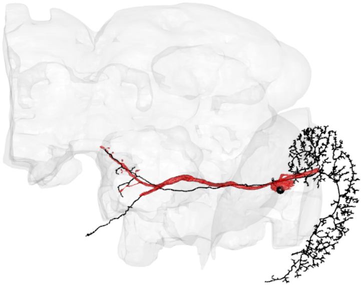

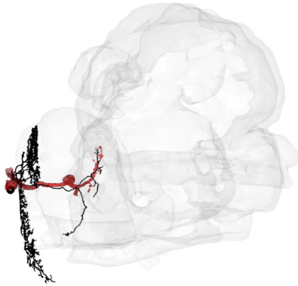

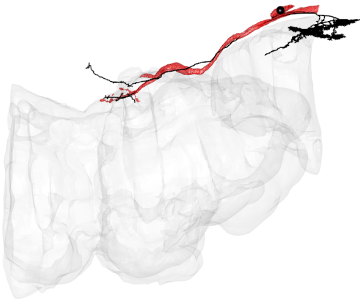

LPT05_R_VS1

CATMAID skid = 982897

Hemibrain bodyid= 5813024262

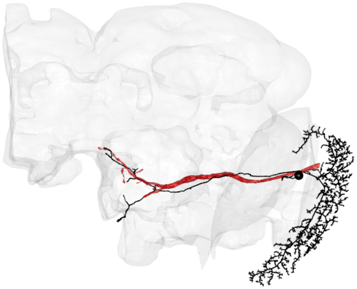

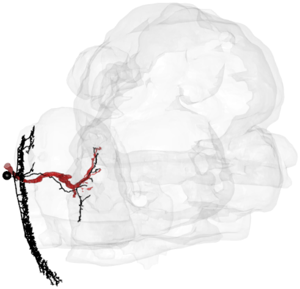

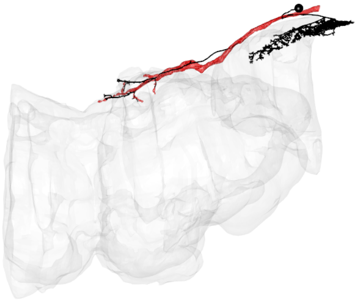

LPT06_R_VS2

CATMAID skid = 793032

Hemibrain bodyid= 1868619183

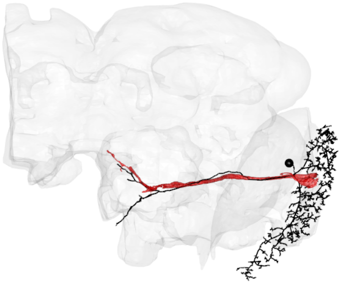

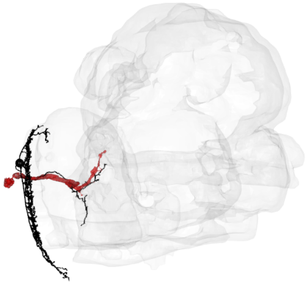

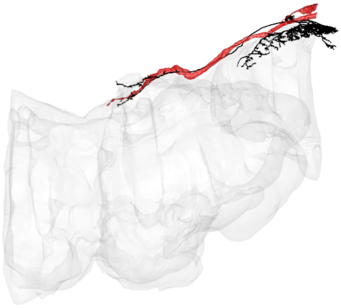

LPT07_R_VS3

CATMAID skid = 815776

Hemibrain bodyid= 5813025091

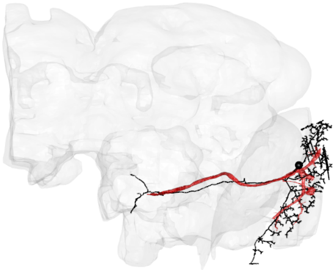

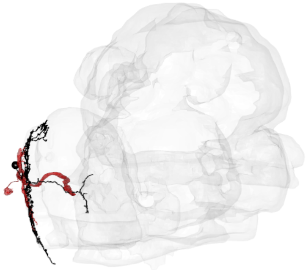

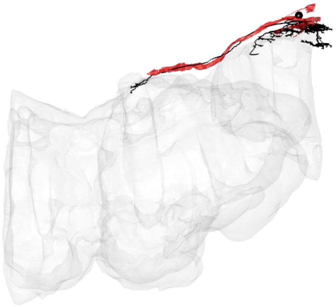

LPT08_R_VS4

CATMAID skid = 17686499

Hemibrain bodyid= 1557885051

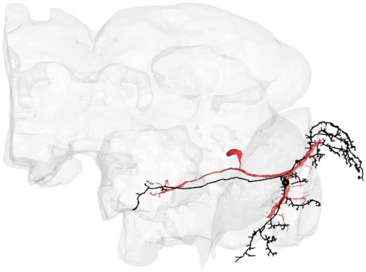

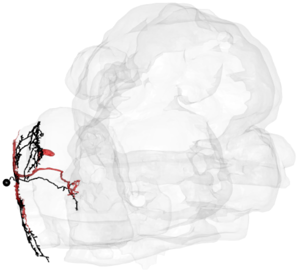

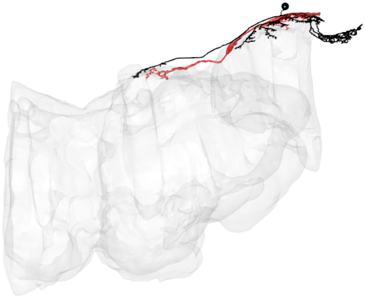

LPT09_R_VS5

CATMAID skid = 807401

Hemibrain bodyid= 5813033533

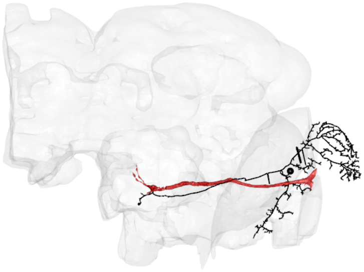

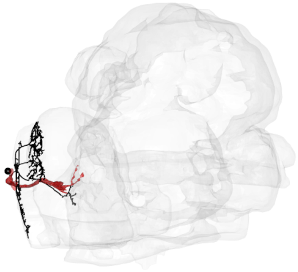

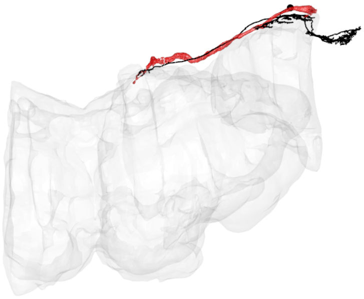

LPT10_R_VS6

CATMAID skid = 804539

Hemibrain bodyid= 1992161820

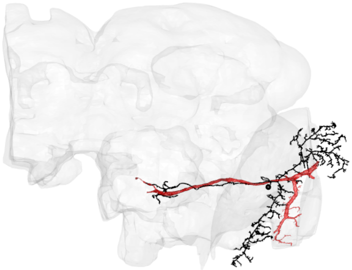

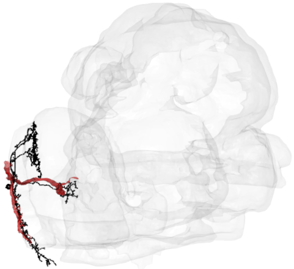

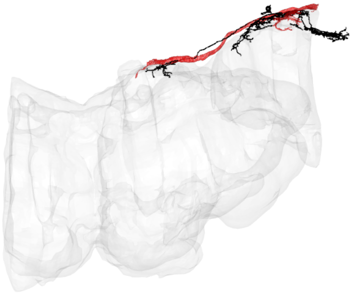

LPT11_R_VS7

CATMAID skid = 851432

Hemibrain bodyid= 1868255513

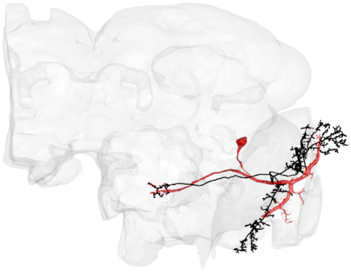

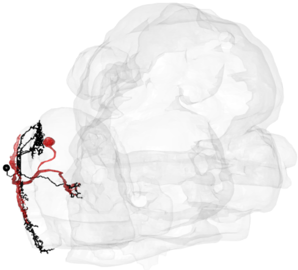

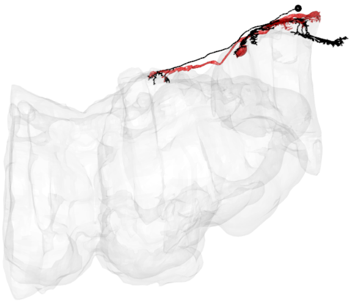

LPT12_R_VS8

CATMAID skid = 804092

Hemibrain bodyid= 1558230909

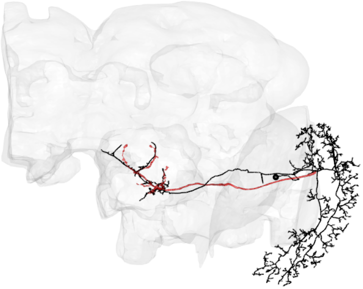

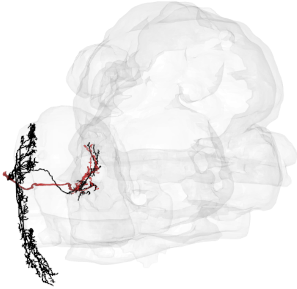

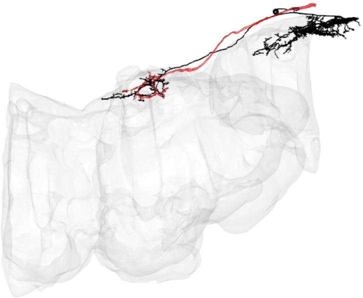

LPT13_R_VSm-1

CATMAID skid = 852286

Hemibrain bodyid= 1836516710

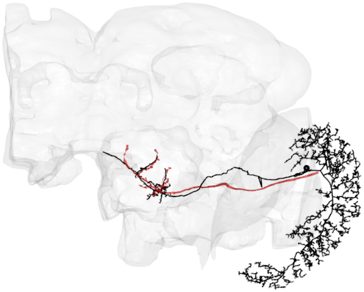

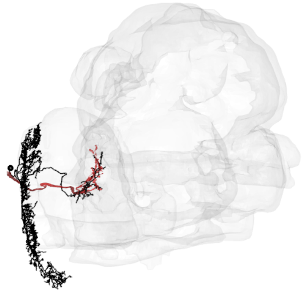

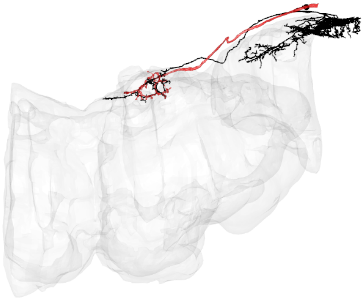

LPT13_R_VSm-2

CATMAID skid = 815241

Hemibrain bodyid= 1805481901

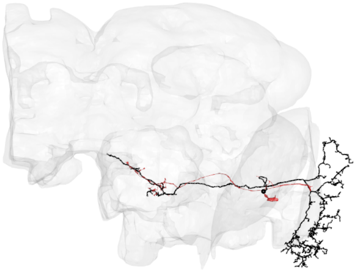

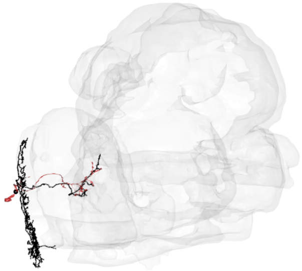

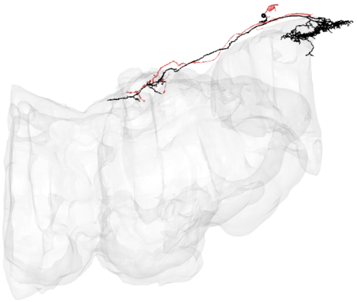

LPT15_R_VST1-2

CATMAID skid = 988674

Hemibrain bodyid= 5812993165

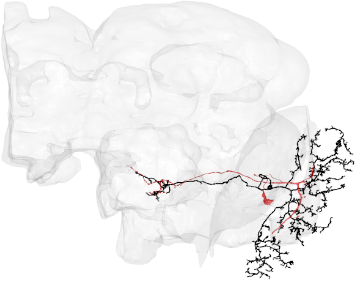

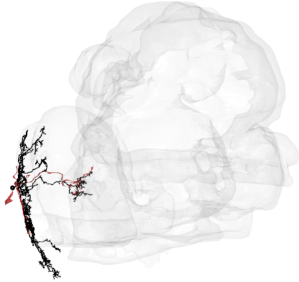

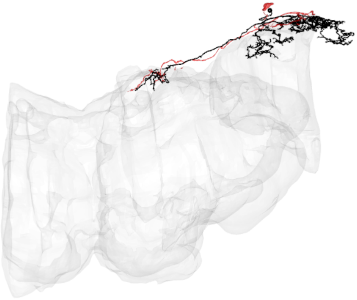

LPT17_R_VST2-1

CATMAID skid = 1123389

Hemibrain bodyid= 1837198715

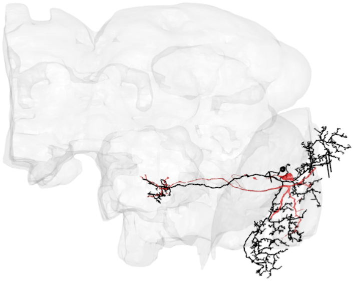

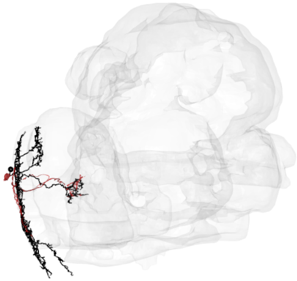

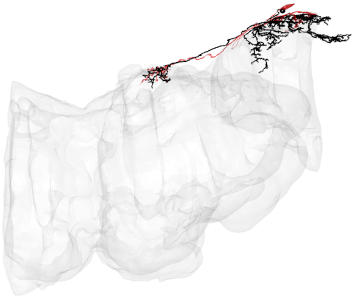

LPT17_R_VST2-2

CATMAID skid = 1112633

Hemibrain bodyid= 5813023313

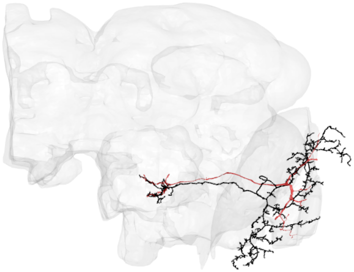

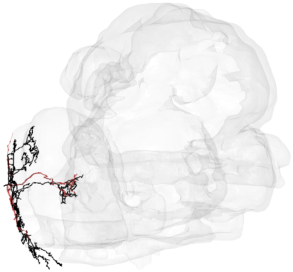

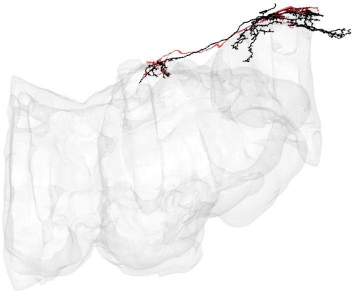

LPT17_R_VST2-3

CATMAID skid = 2852912

Hemibrain bodyid= 5813049185

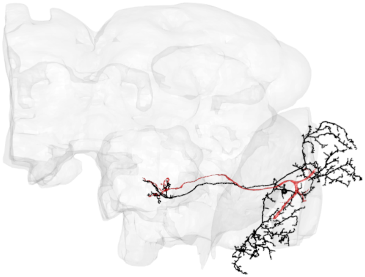

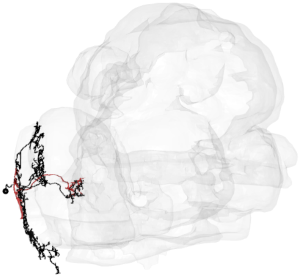

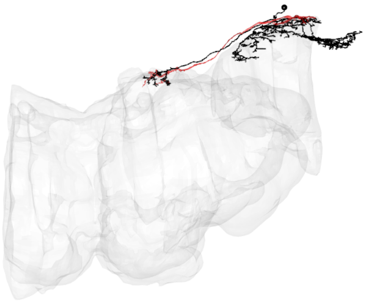

LPT17_R_VST2-4

CATMAID skid = 5031615

Hemibrain bodyid= 5813023581

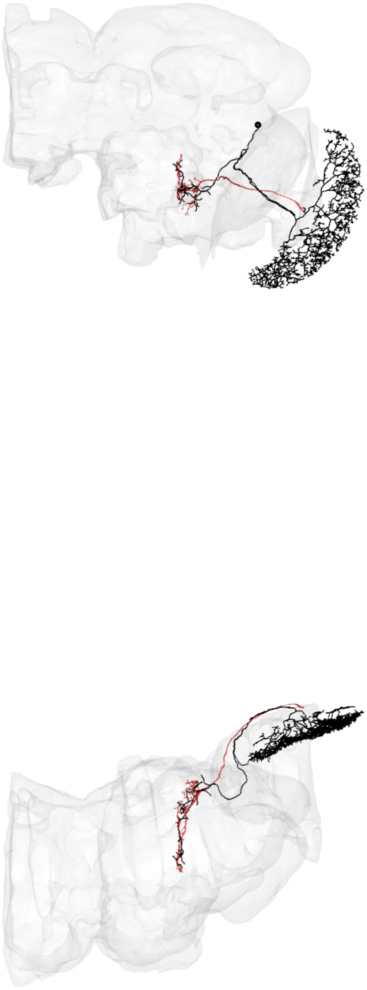

LPT21_R

CATMAID skid = 1110765

Hemibrain bodyid= 1850310331

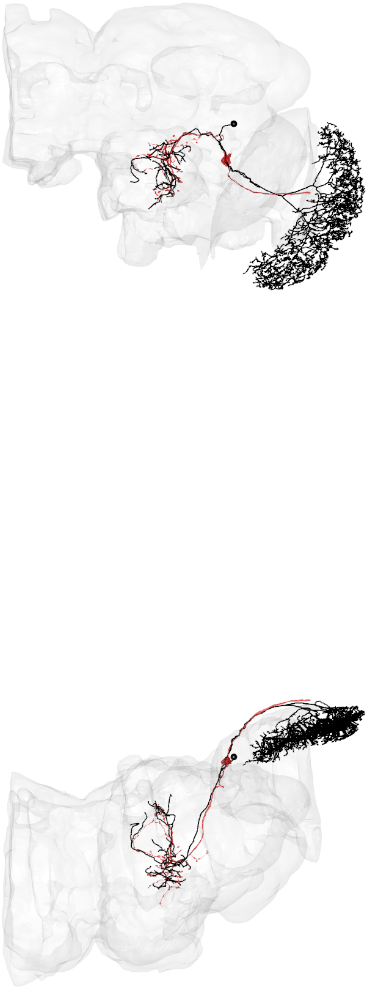

LPT22_R

CATMAID skid = 3510999

Hemibrain bodyid= 1501708149

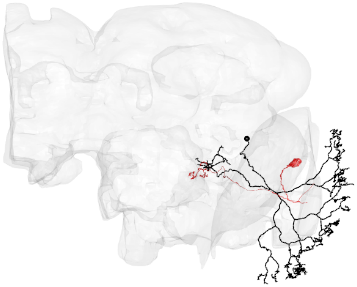

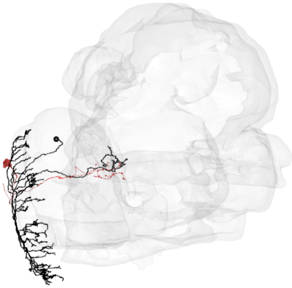

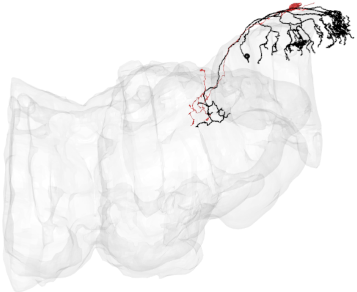

LPT23_R-1

CATMAID skid = 4135042

Hemibrain bodyid= 1842090544

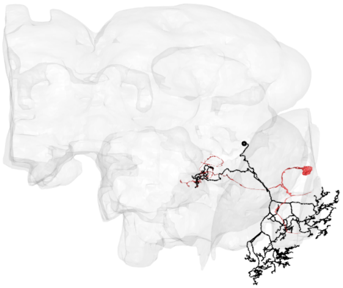

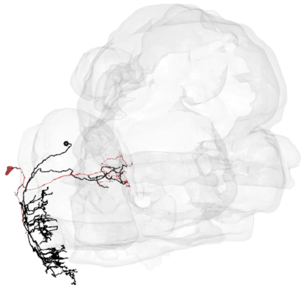

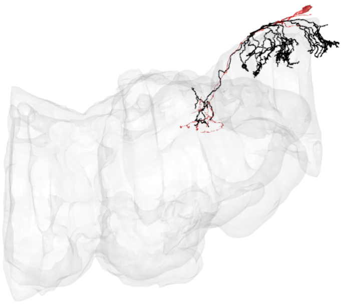

LPT23_R-2

CATMAID skid = 4504548

Hemibrain bodyid= 1751364895

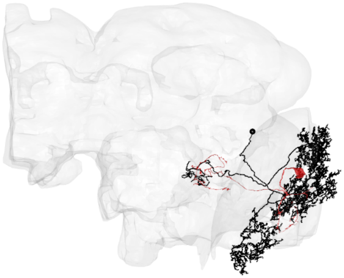

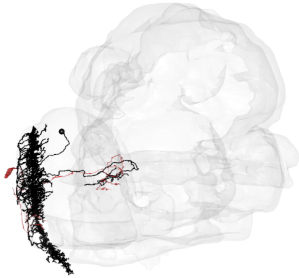

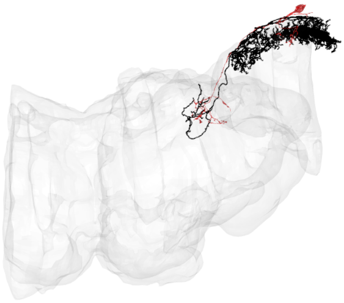

LPT23_R-3

CATMAID skid = 4504557

Hemibrain bodyid= 1751028010

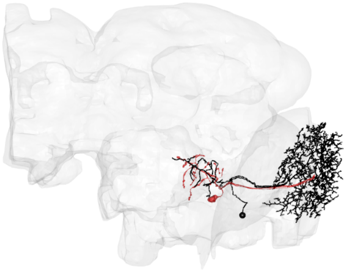

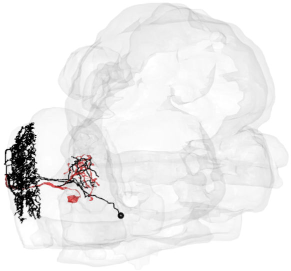

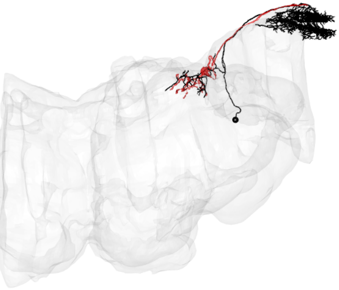

LPT26_R

CATMAID skid = 1107045

Hemibrain bodyid= 1747289181

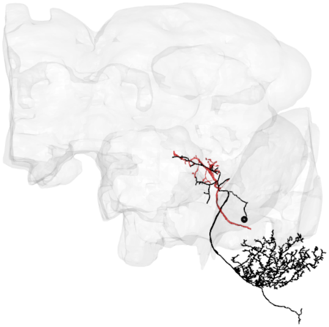

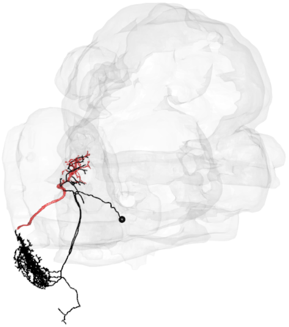

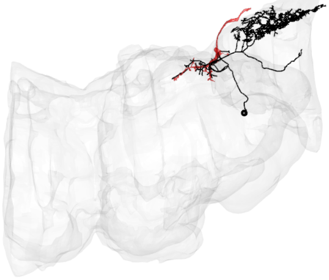

LPT27_R

CATMAID skid = 3514698

Hemibrain bodyid= 1747608690

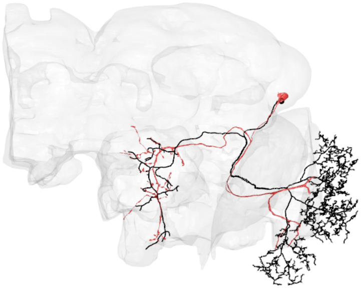

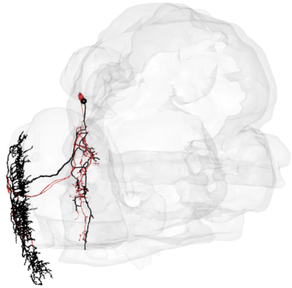

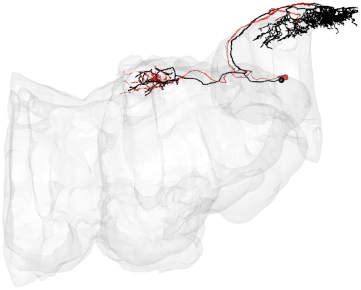

LPT28_R

CATMAID skid = 1071860

Hemibrain bodyid= 1407904797

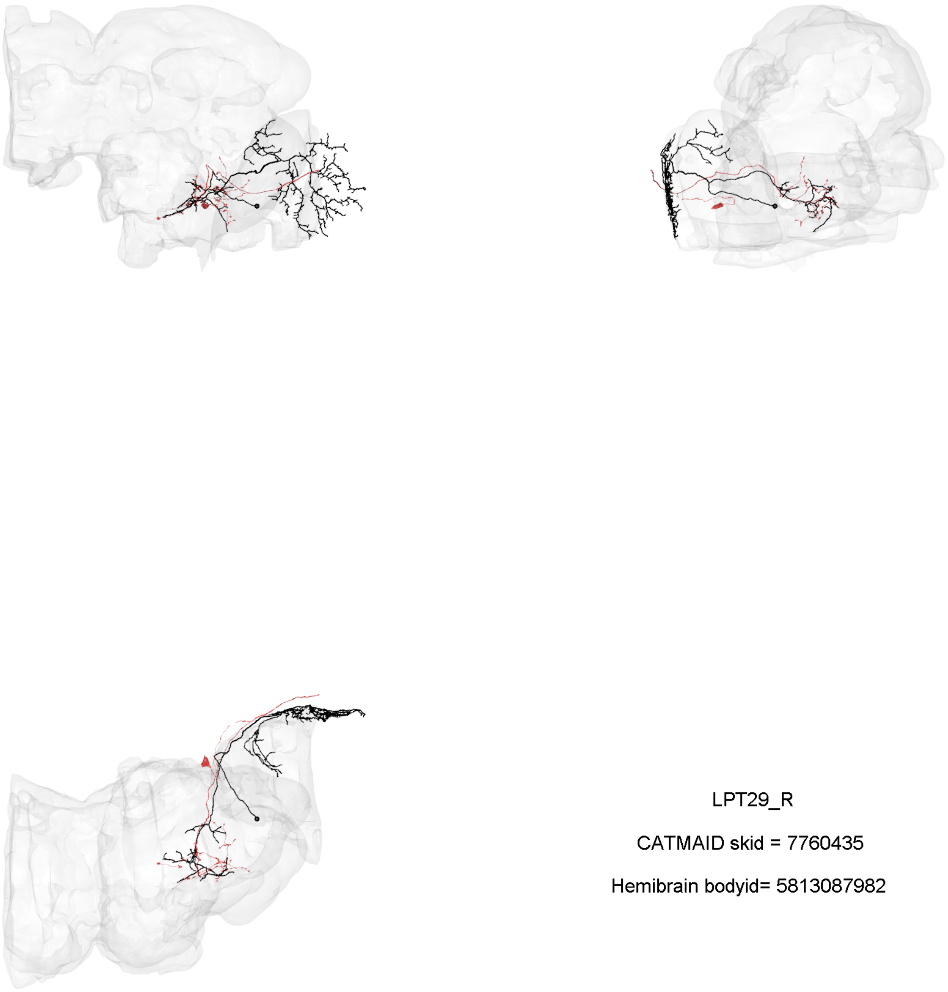

LPT29_R

CATMAID skid = 7760435

Hemibrain bodyid= 5813087982

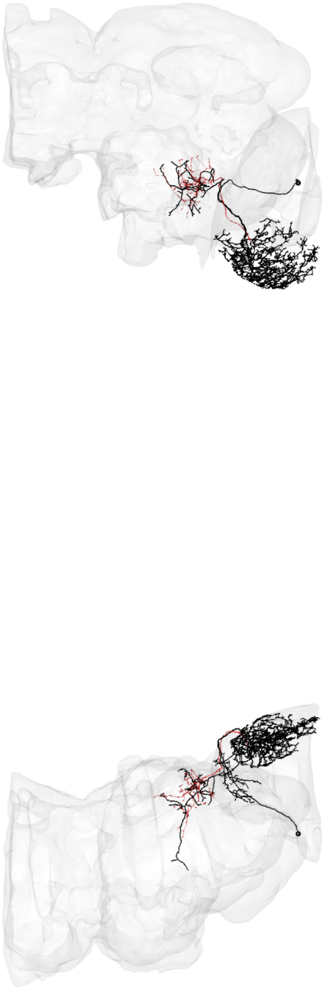

LPT30_R

CATMAID skid = 7674107

Hemibrain bodyid= 1777301335

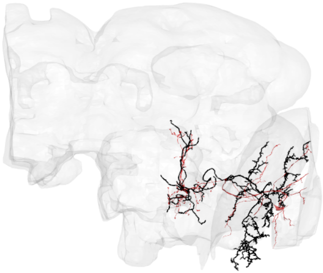

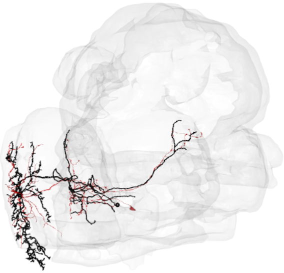

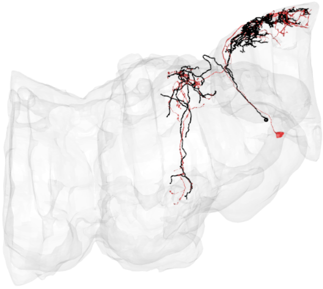

LPT31_R-1

CATMAID skid = 7690273

Hemibrain bodyid= 1622471963

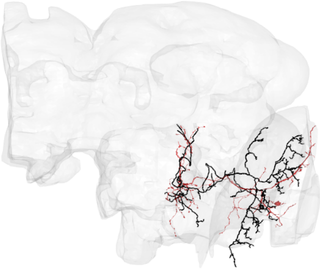

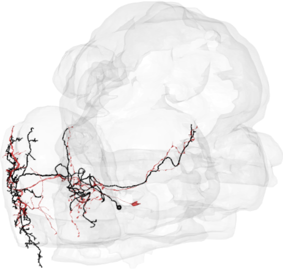

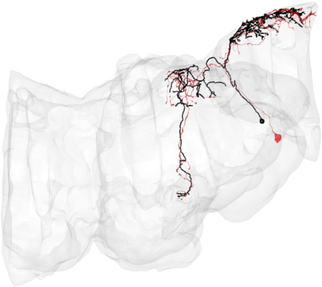

LPT31_R-2

CATMAID skid = 7311408

Hemibrain bodyid= 1434427171

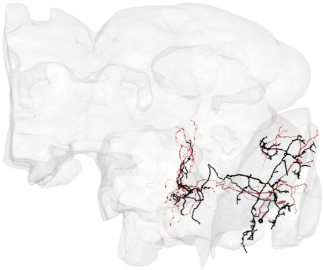

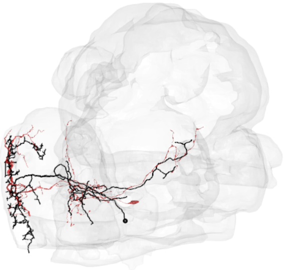

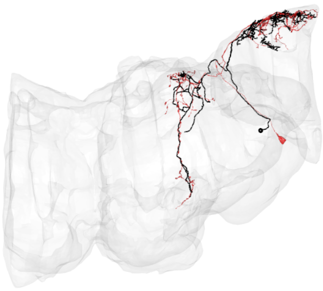

LPT31_R-3

CATMAID skid = 7311493

Hemibrain bodyid= 1434427905

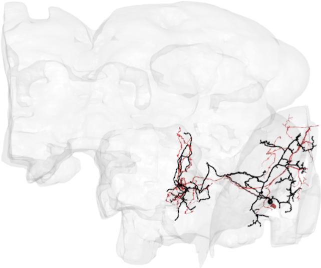

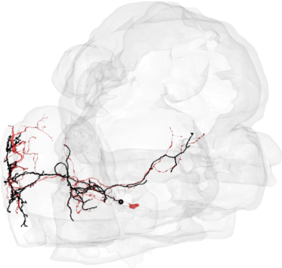

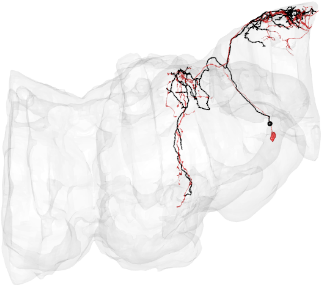

LPT31_R-4

CATMAID skid = 7694305

Hemibrain bodyid= 1344082025

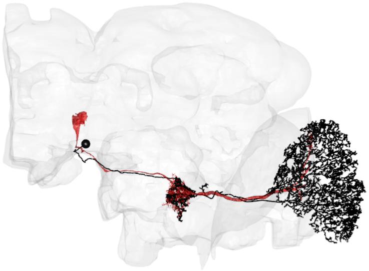
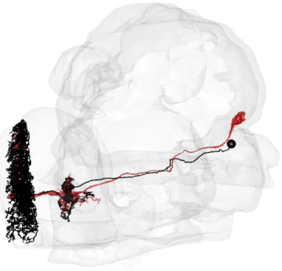
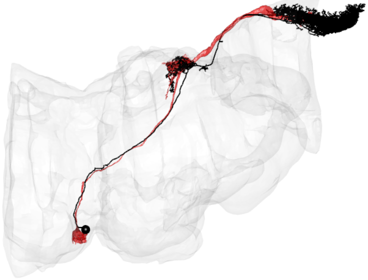

LPT35_R_dCH

CATMAID skid = 1077174

Hemibrain bodyid= 1466485353

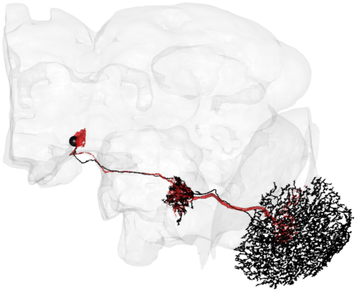

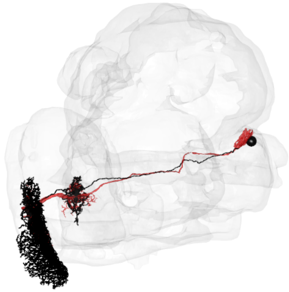

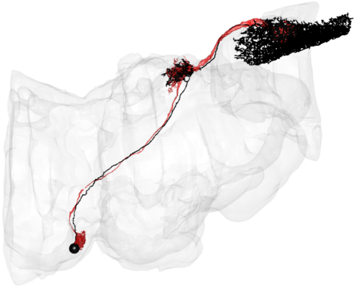

LPT36_R_vCH

CATMAID skid = 1078535

Hemibrain bodyid= 5813024201

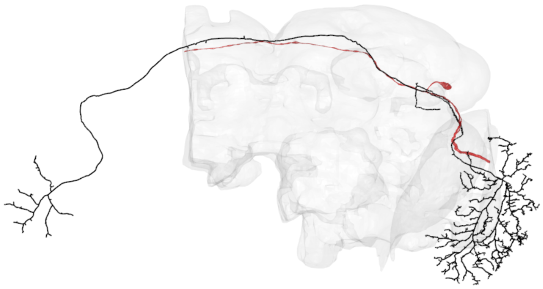

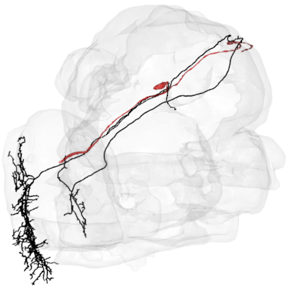

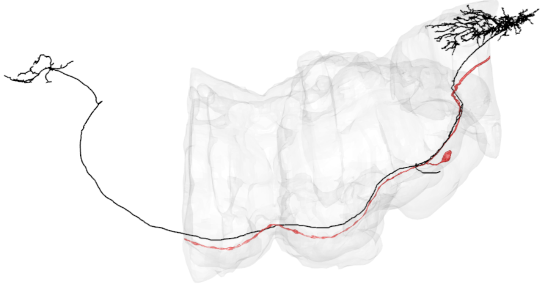

LPT37_R_H1

CATMAID skid = 1121730

Hemibrain bodyid= 676832896

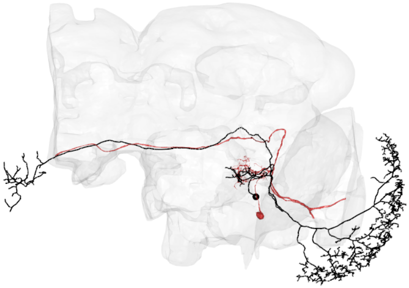

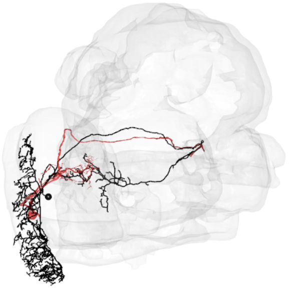

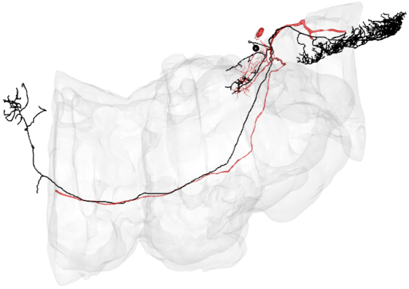

LPT38_R_Nod1-1

CATMAID skid = 1054753

Hemibrain bodyid= 5812996970

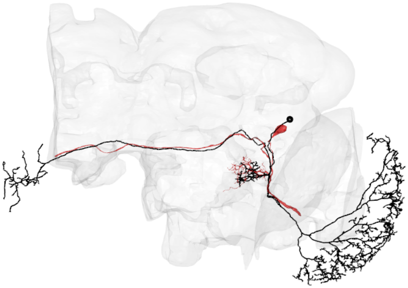

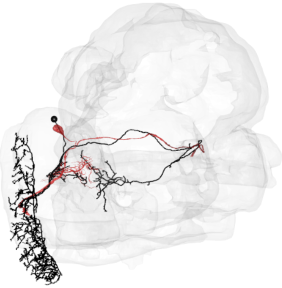

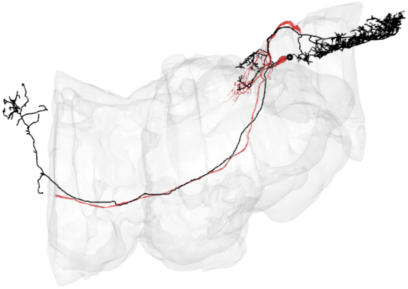

LPT38_R_Nod1-2

CATMAID skid = 3546483

Hemibrain bodyid= 5812993603

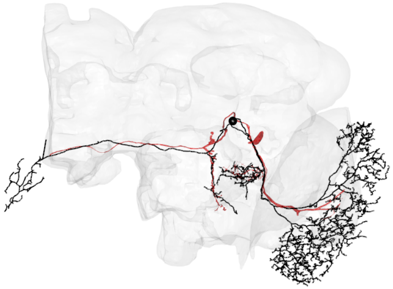

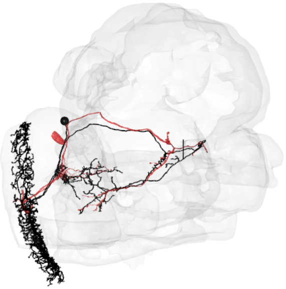

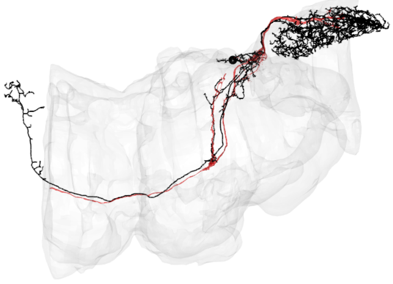

LPT40_R_Nod2

CATMAID skid = 902072

Hemibrain bodyid= 1315529069

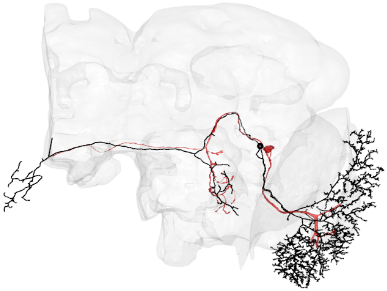

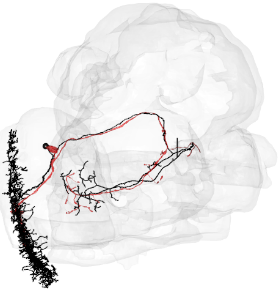

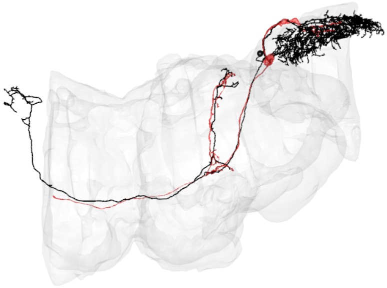

LPT41_R_Nod3

CATMAID skid = 1058996

Hemibrain bodyid= 1352706891

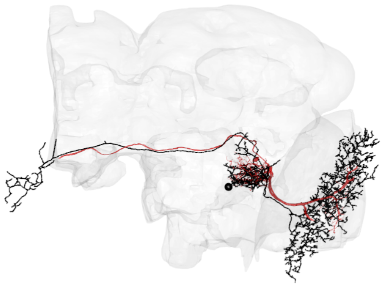

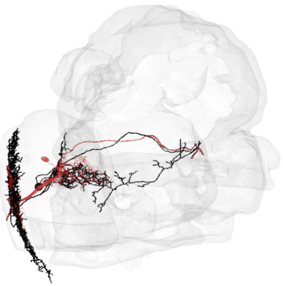

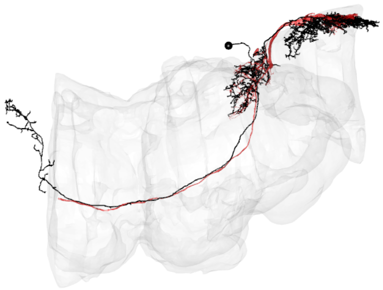

LPT42_R_Nod4

CATMAID skid = 1106958

Hemibrain bodyid= 1566524544

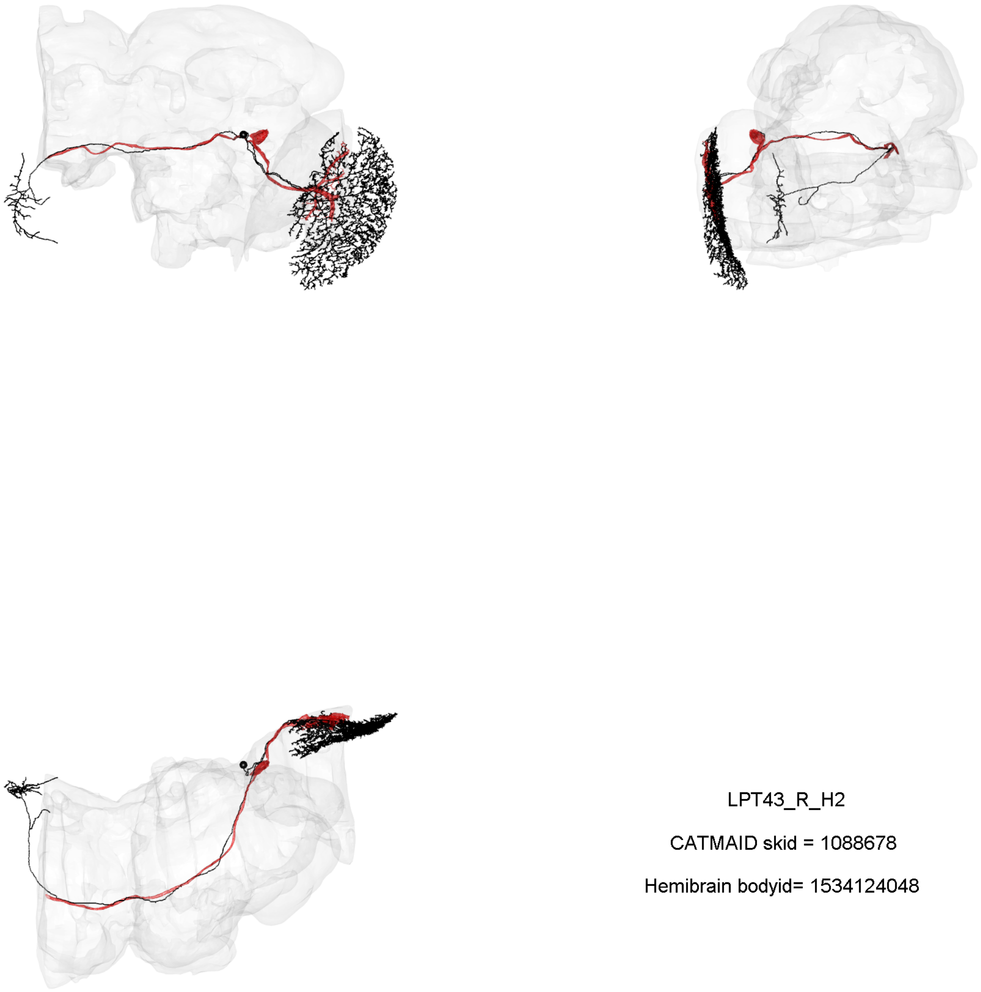

LPT43_R_H2

CATMAID skid = 1088678

Hemibrain bodyid= 1534124048

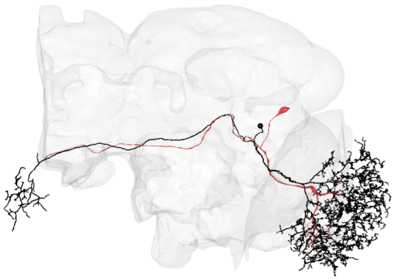

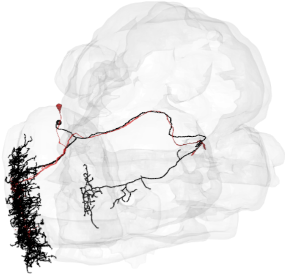

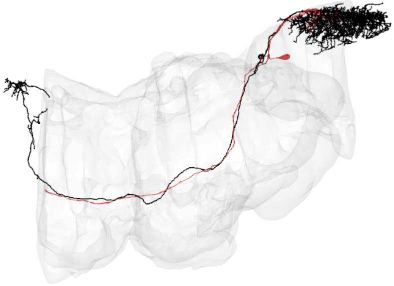

LPT44_R_Nod5

CATMAID skid = 1121795

Hemibrain bodyid= 1899956171

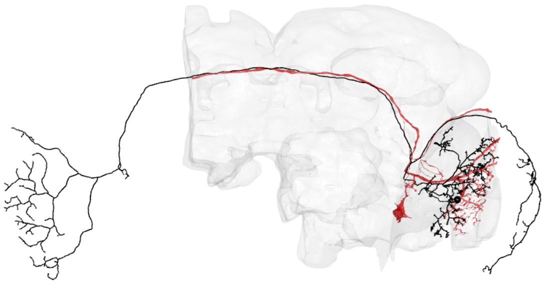

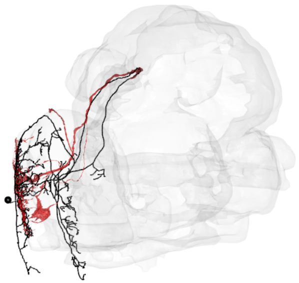

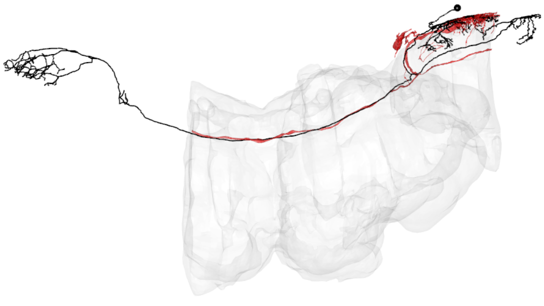

LPT45_R_dCal1

CATMAID skid = 7204844

Hemibrain bodyid= 943813788

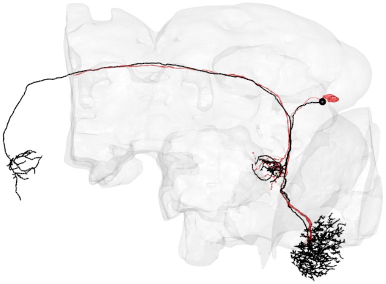

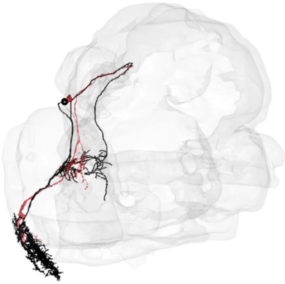

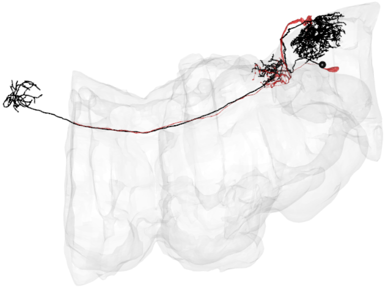

LPT46_R_vCal1

CATMAID skid = 7510076

Hemibrain bodyid= 1005174931

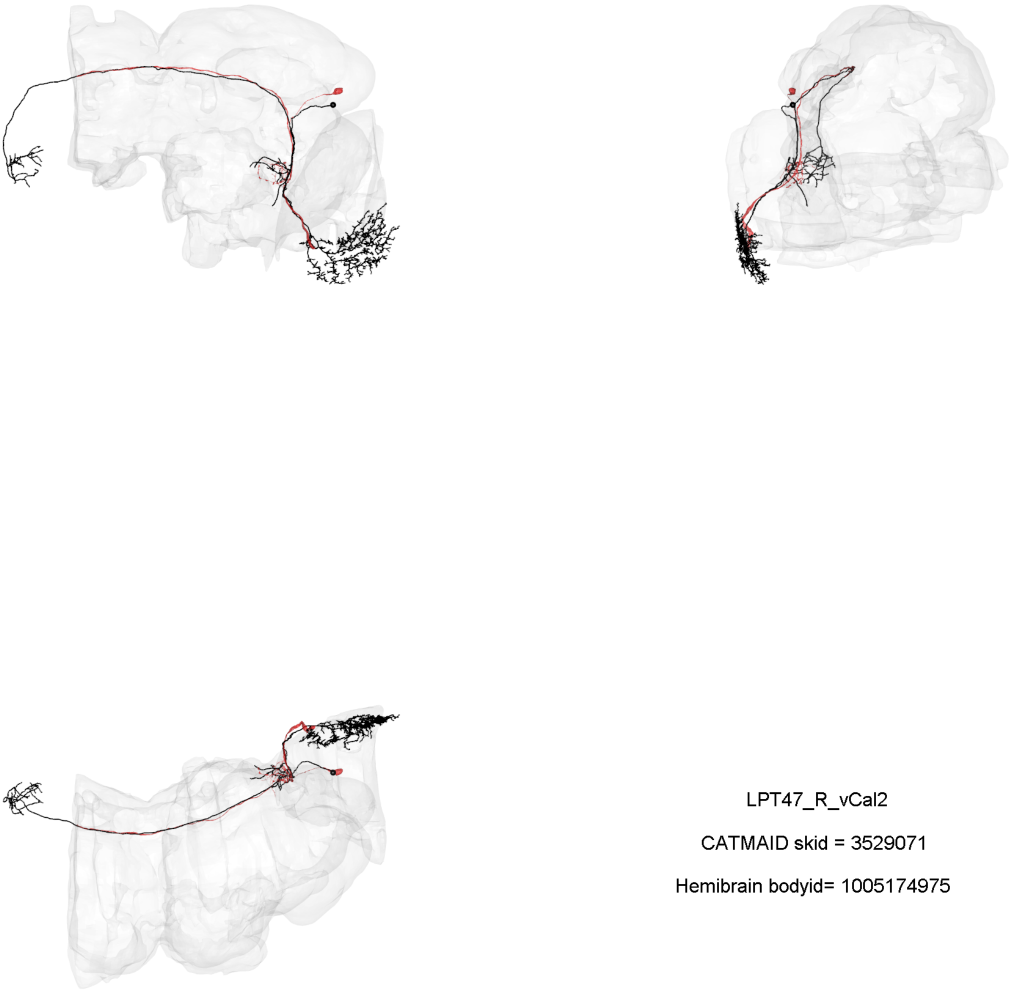

LPT47_R_vCal2

CATMAID skid = 3529071

Hemibrain bodyid= 1005174975

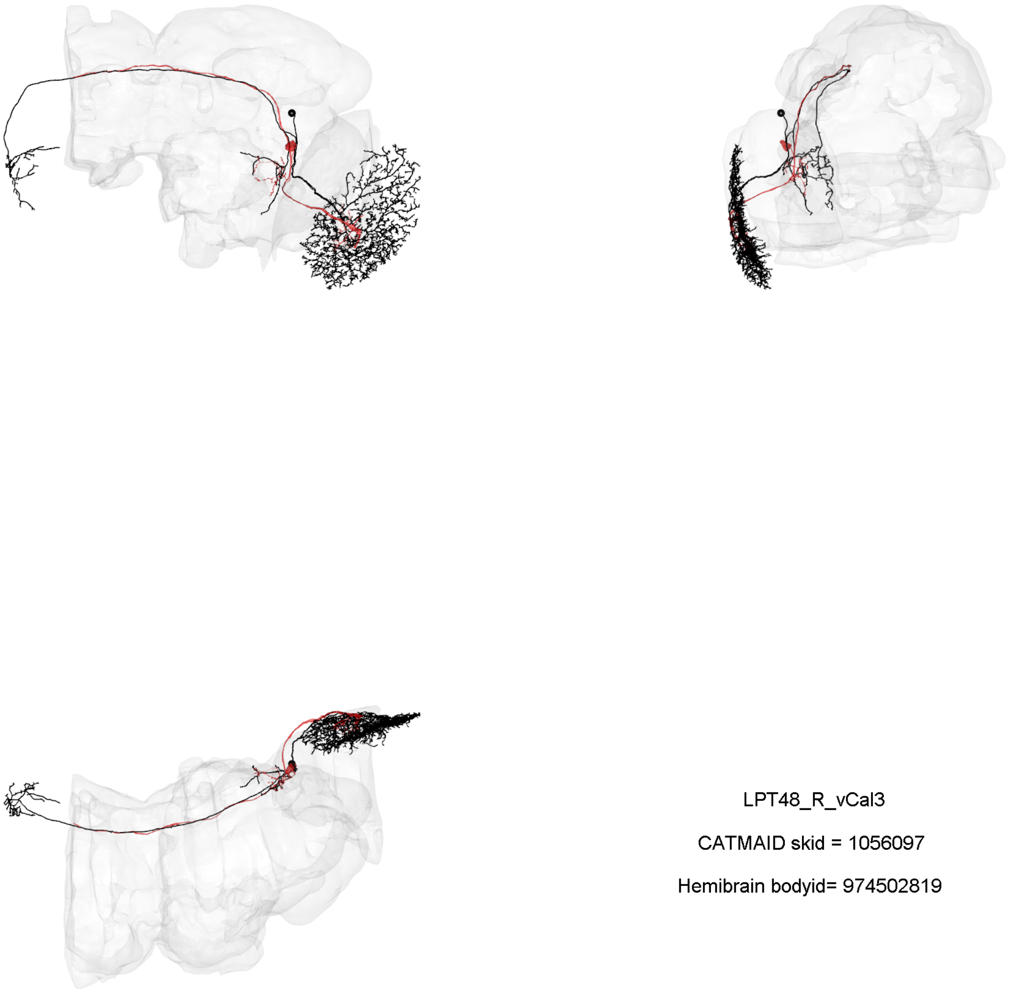

LPT48_R_vCal3

CATMAID skid = 1056097

Hemibrain bodyid= 974502819

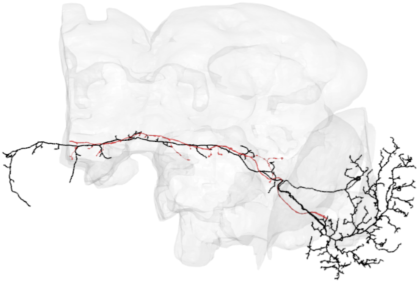

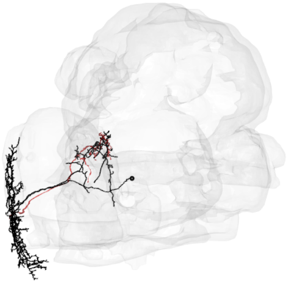

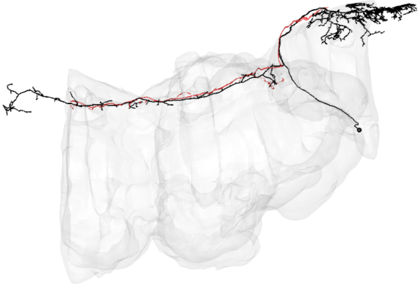

LPT49_R

CATMAID skid = 1110693

Hemibrain bodyid= 1654969939

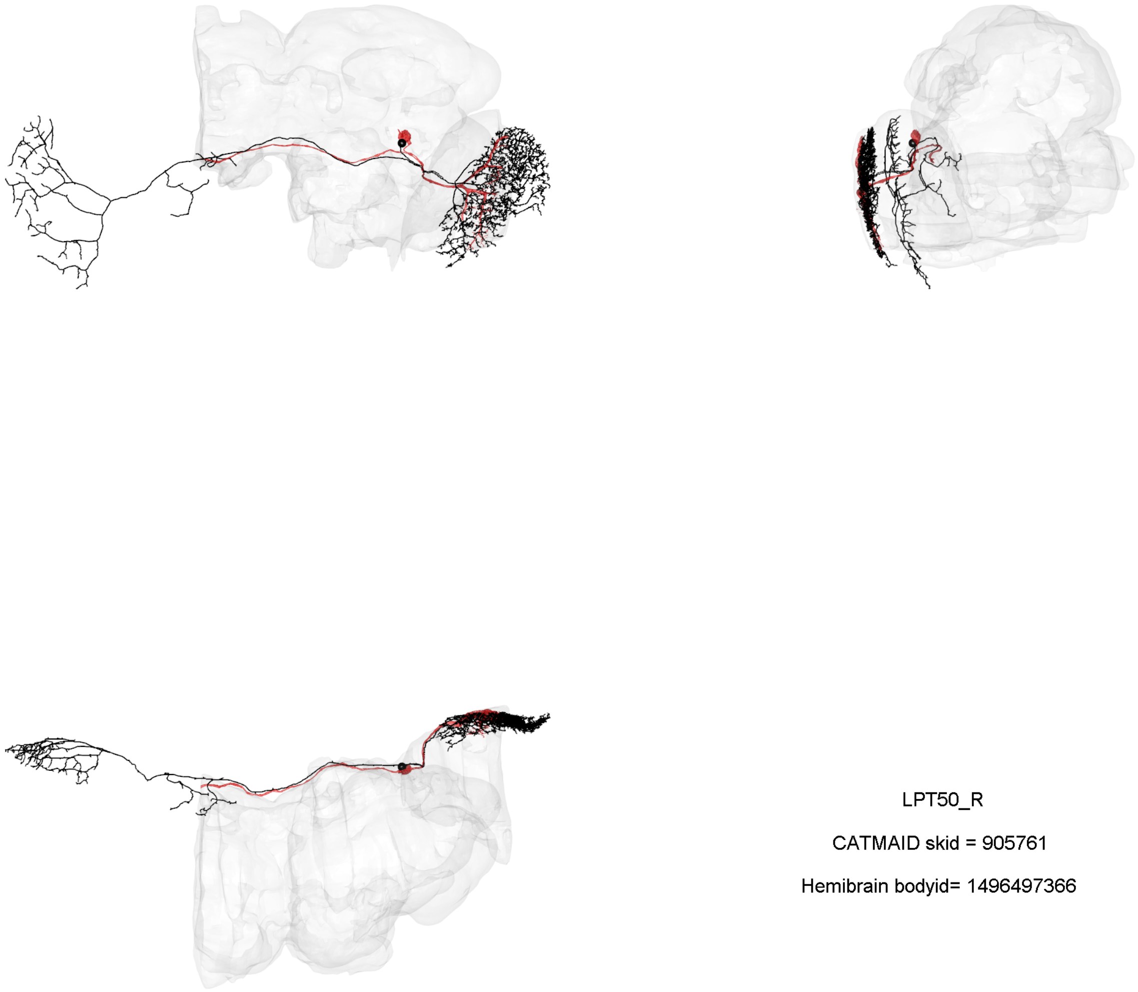

LPT50_R

CATMAID skid = 905761

Hemibrain bodyid= 1496497366

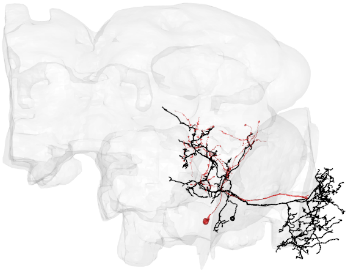

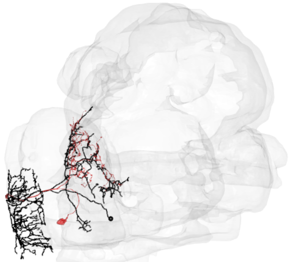

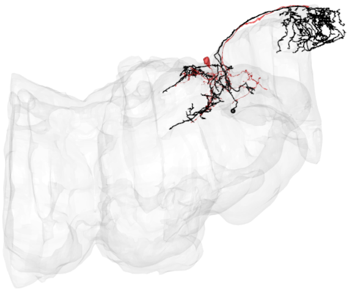

LPT51_R

CATMAID skid = 4224711

Hemibrain bodyid= 1313496323

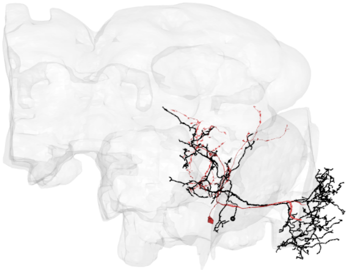

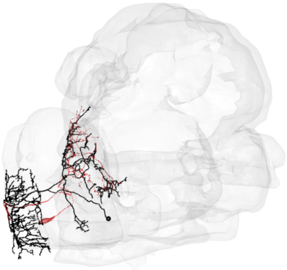

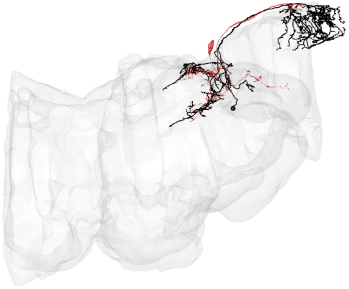

LPT51_R

CATMAID skid = 4224711

Hemibrain bodyid= 1282474090

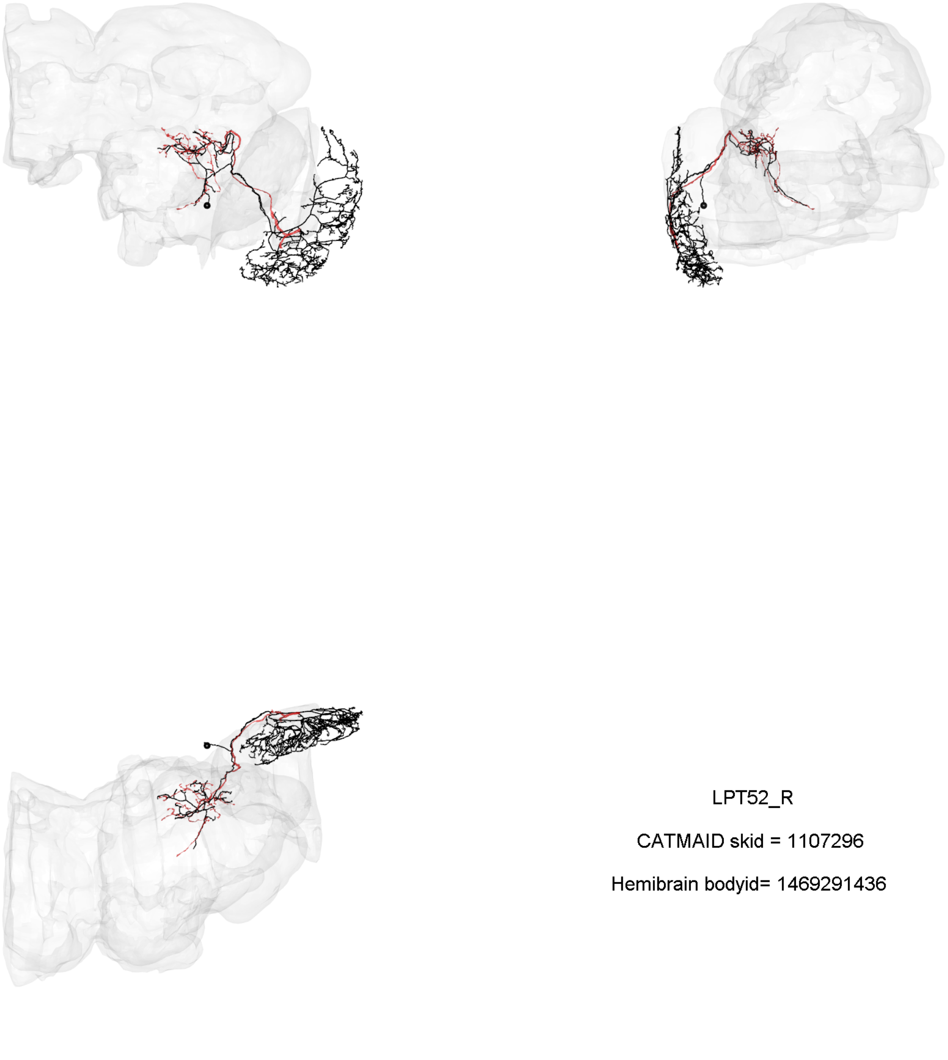

LPT52_R

CATMAID skid = 1107296

Hemibrain bodyid= 1469291436

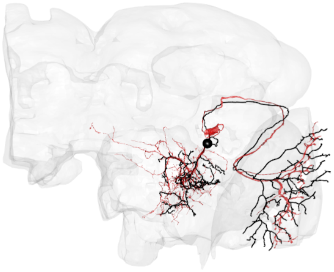

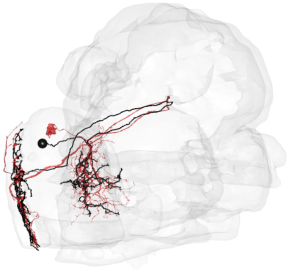

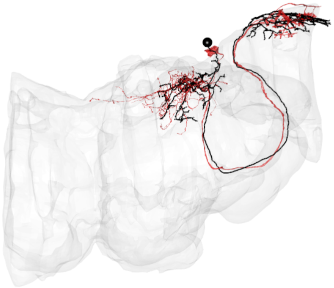

LPT53_R

CATMAID skid = 1111992

Hemibrain bodyid= 1231610379

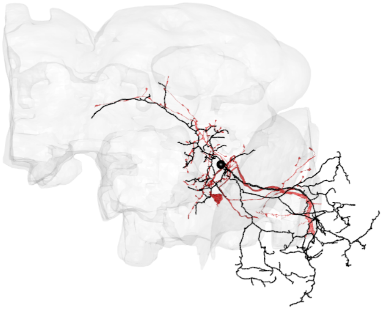

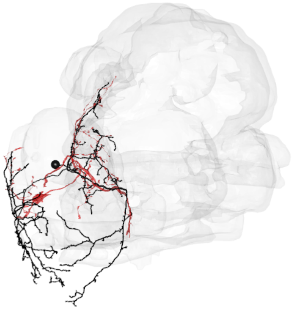

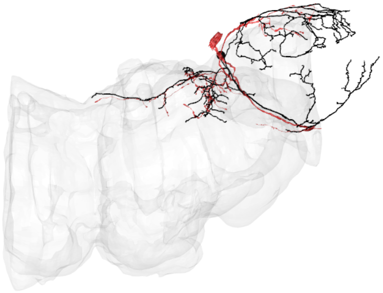

LPT54_R

CATMAID skid = 4235388

Hemibrain bodyid= 1129033939

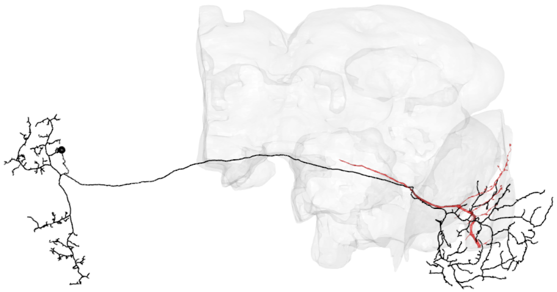

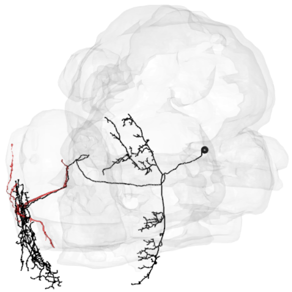

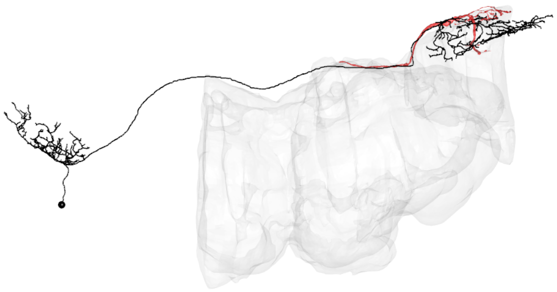

LPT55_R_MeLp2

CATMAID skid = 1061368

Hemibrain bodyid= 1558226341

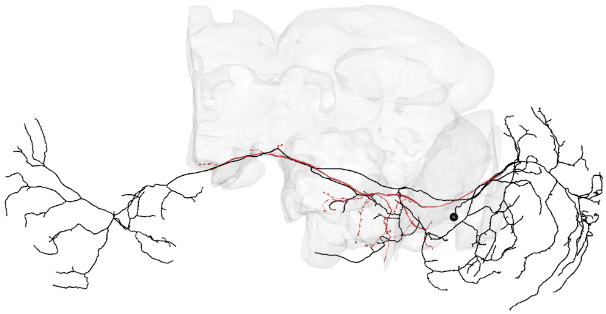

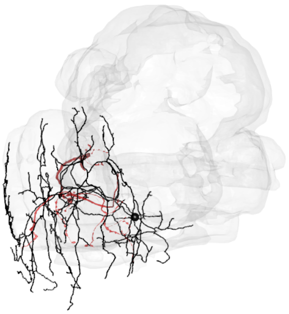

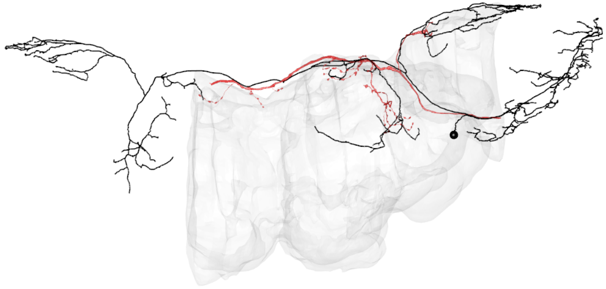

LPT56_R_MeLp1

CATMAID skid = 3509520

Hemibrain bodyid= 5813068976

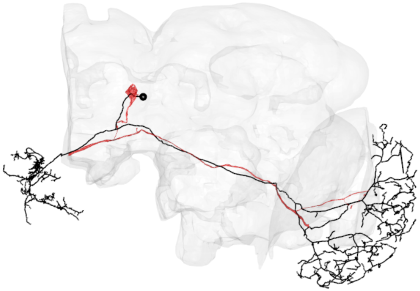

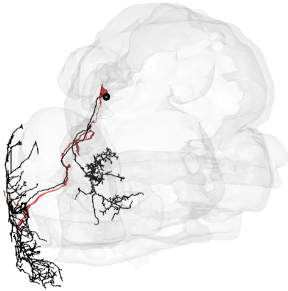

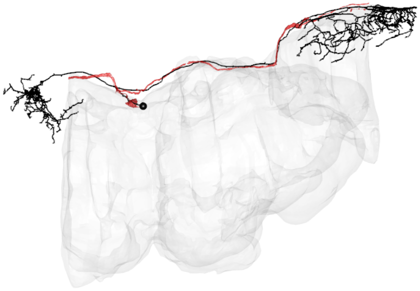

LPT57_R

CATMAID skid = 886797

Hemibrain bodyid= 5901198180

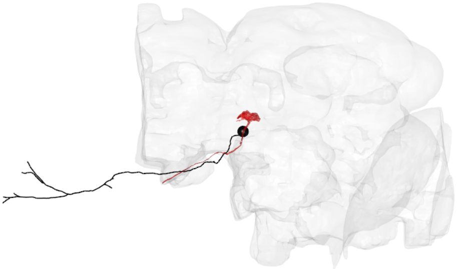

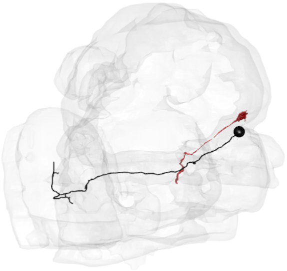

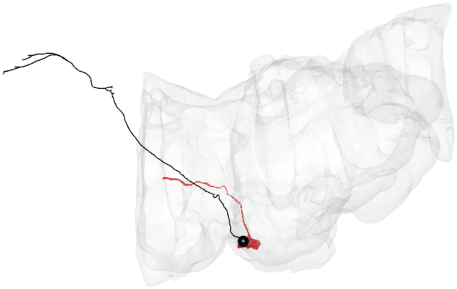

LPT35_L_dCH

CATMAID skid = 6243409

Hemibrain bodyid= 1545158404

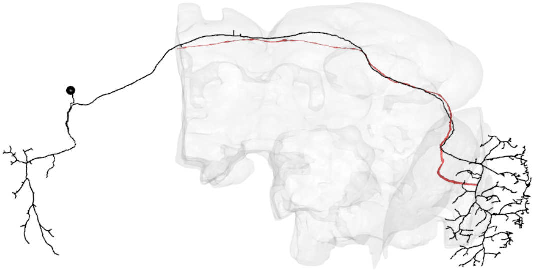

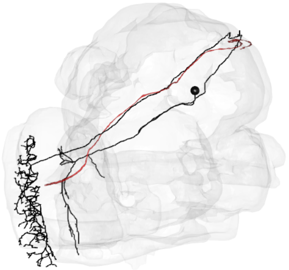

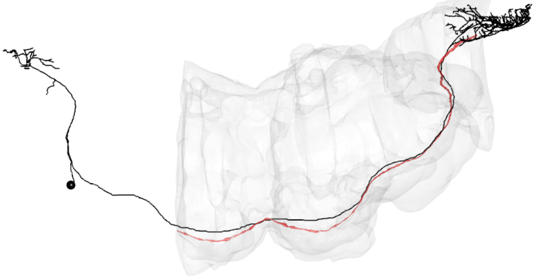

LPT37_L_H1

CATMAID skid = 1121335

Hemibrain bodyid= 1167783603

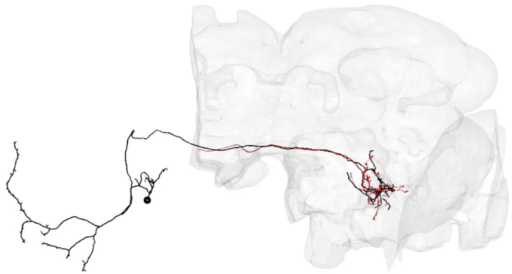

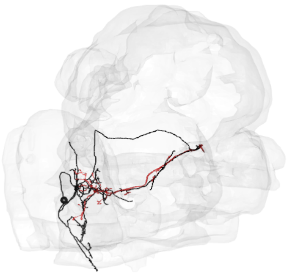

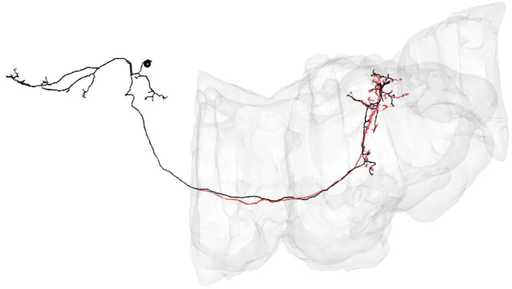

LPT38_L_Nod1-1

CATMAID skid = 6439204

Hemibrain bodyid= 1789306586

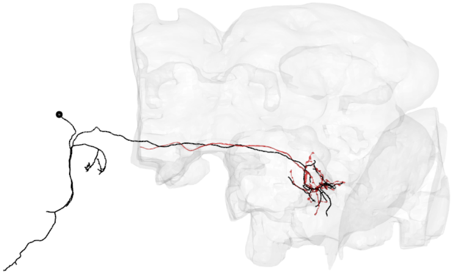

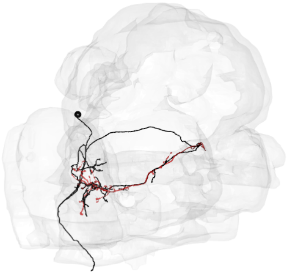

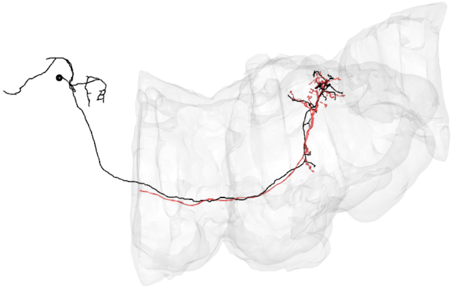

LPT38_L_Nod1-2

CATMAID skid = 6437945

Hemibrain bodyid= 1758621675

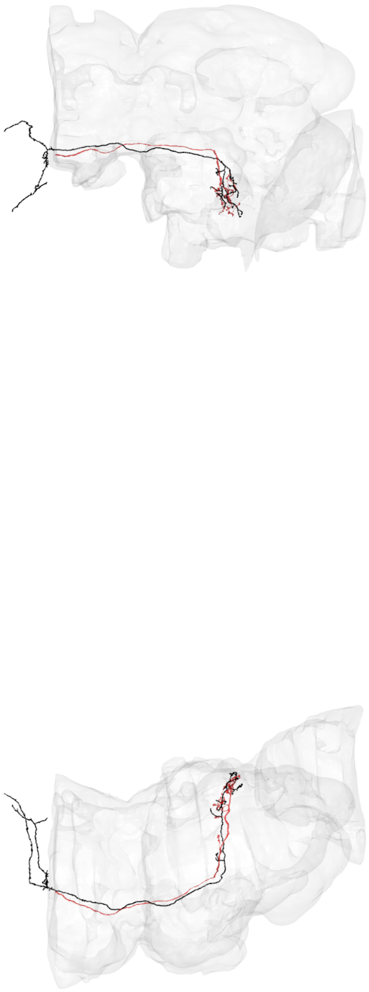

LPT40_L_Nod2

CATMAID skid = 16615842

Hemibrain bodyid= 1871778911

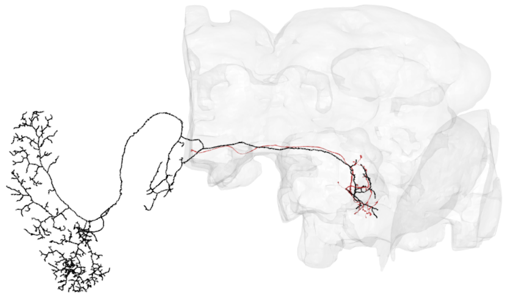

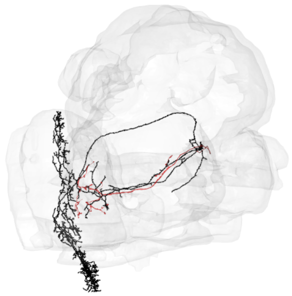

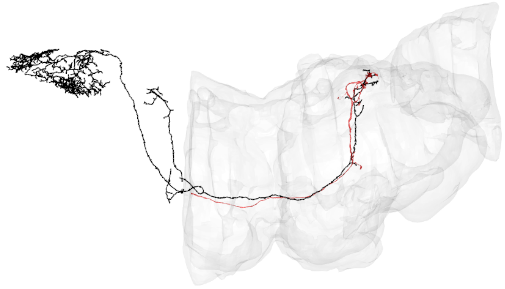

LPT41_L_Nod3

CATMAID skid = 15060018

Hemibrain bodyid= 1758617327

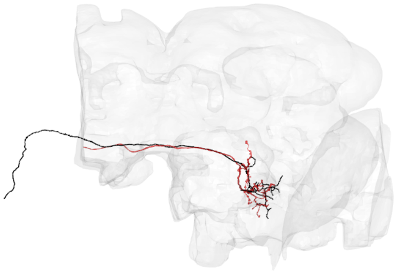
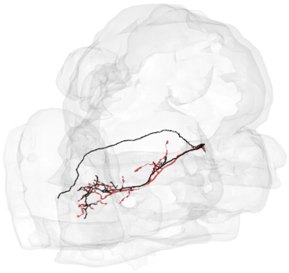
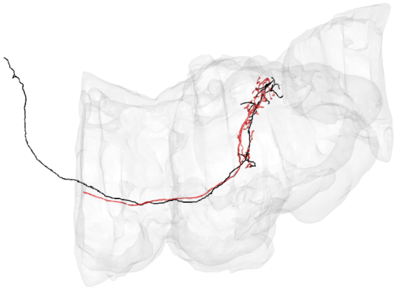

LPT42_L_Nod4

CATMAID skid = 16615419

Hemibrain bodyid= 1725837767

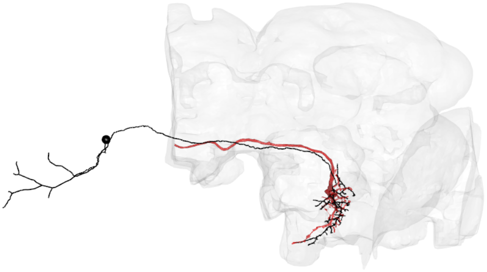

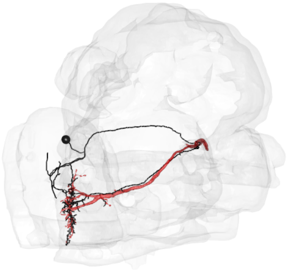

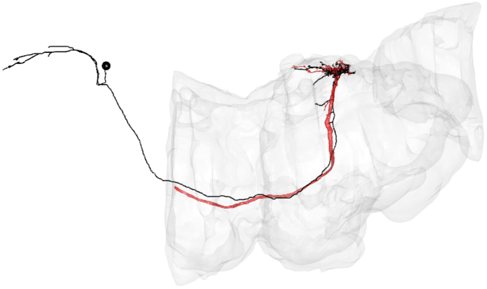

LPT43_L_H2

CATMAID skid = 5232902

Hemibrain bodyid= 5813078454

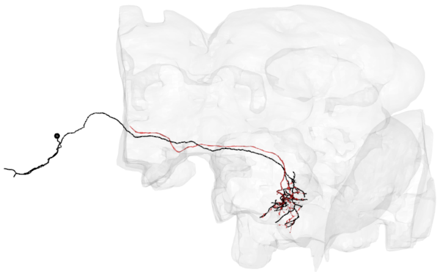

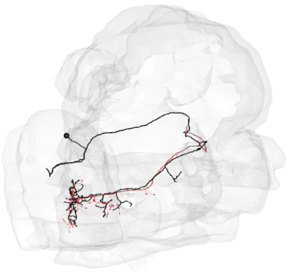

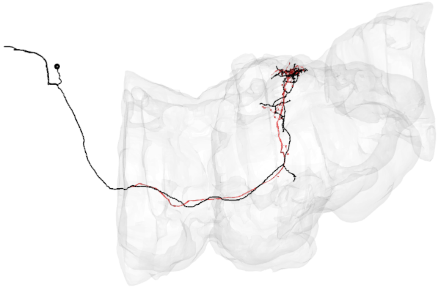

LPT44_L_Nod5

CATMAID skid = 7231304

Hemibrain bodyid= 5812994338

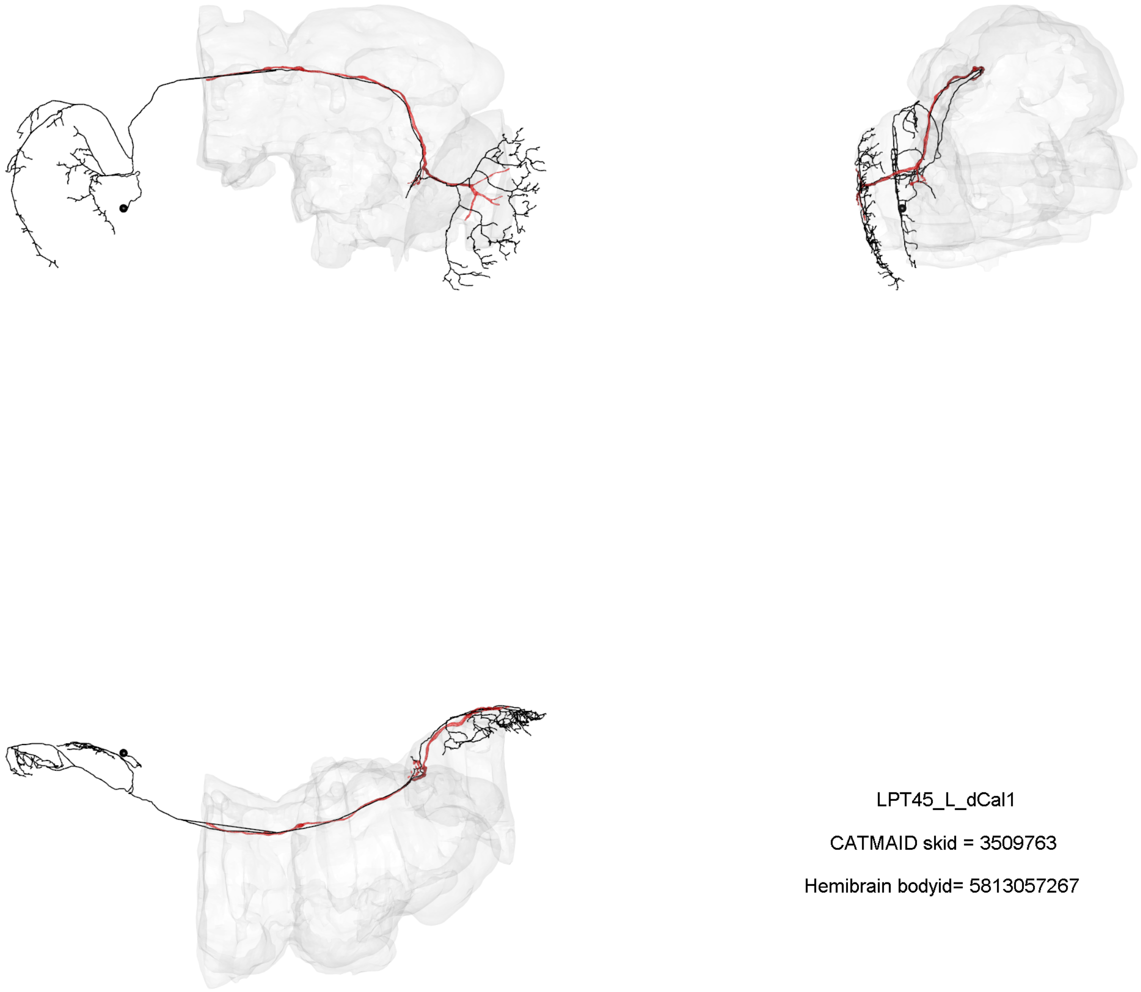

LPT45_L_dCal1

CATMAID skid = 3509763

Hemibrain bodyid= 5813057267

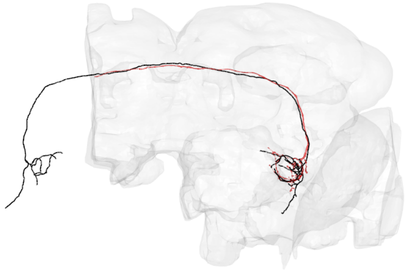

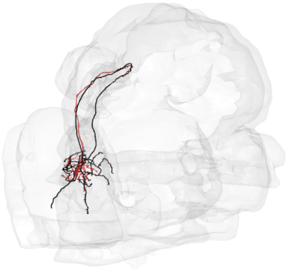

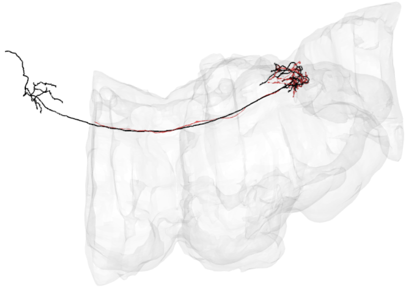

LPT46_L_vCal1

CATMAID skid = 8747266

Hemibrain bodyid= 943468720

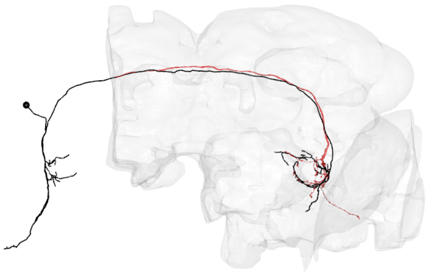

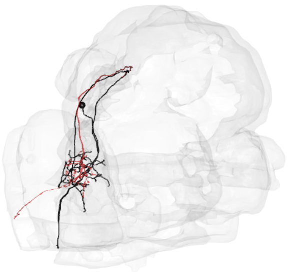

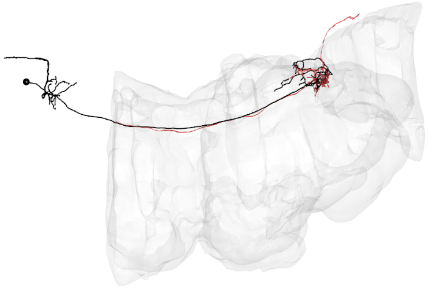

LPT47_L_vCal2

CATMAID skid = 7449616

Hemibrain bodyid= 943472755

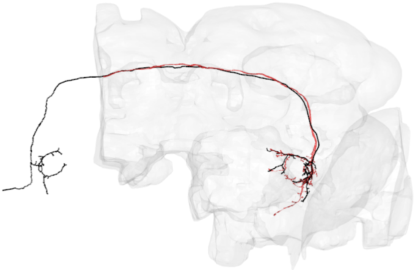

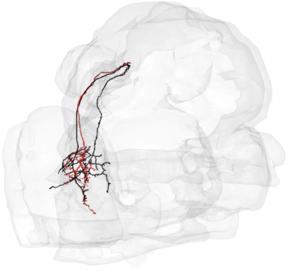

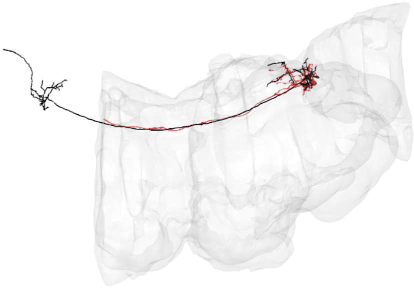

LPT48_L_vCal3

CATMAID skid = 11230125

Hemibrain bodyid= 943472763

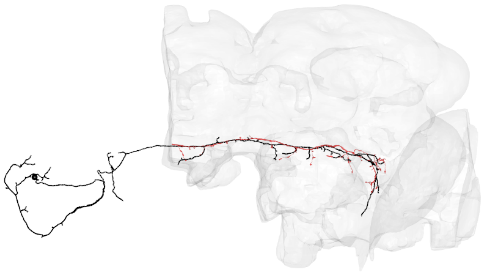
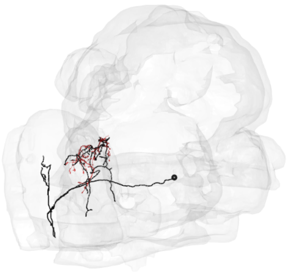
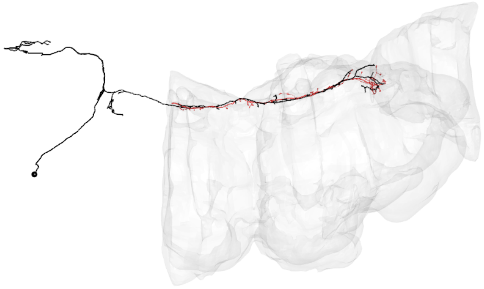

LPT49_L

CATMAID skid = 17072553

Hemibrain bodyid= 1685896788

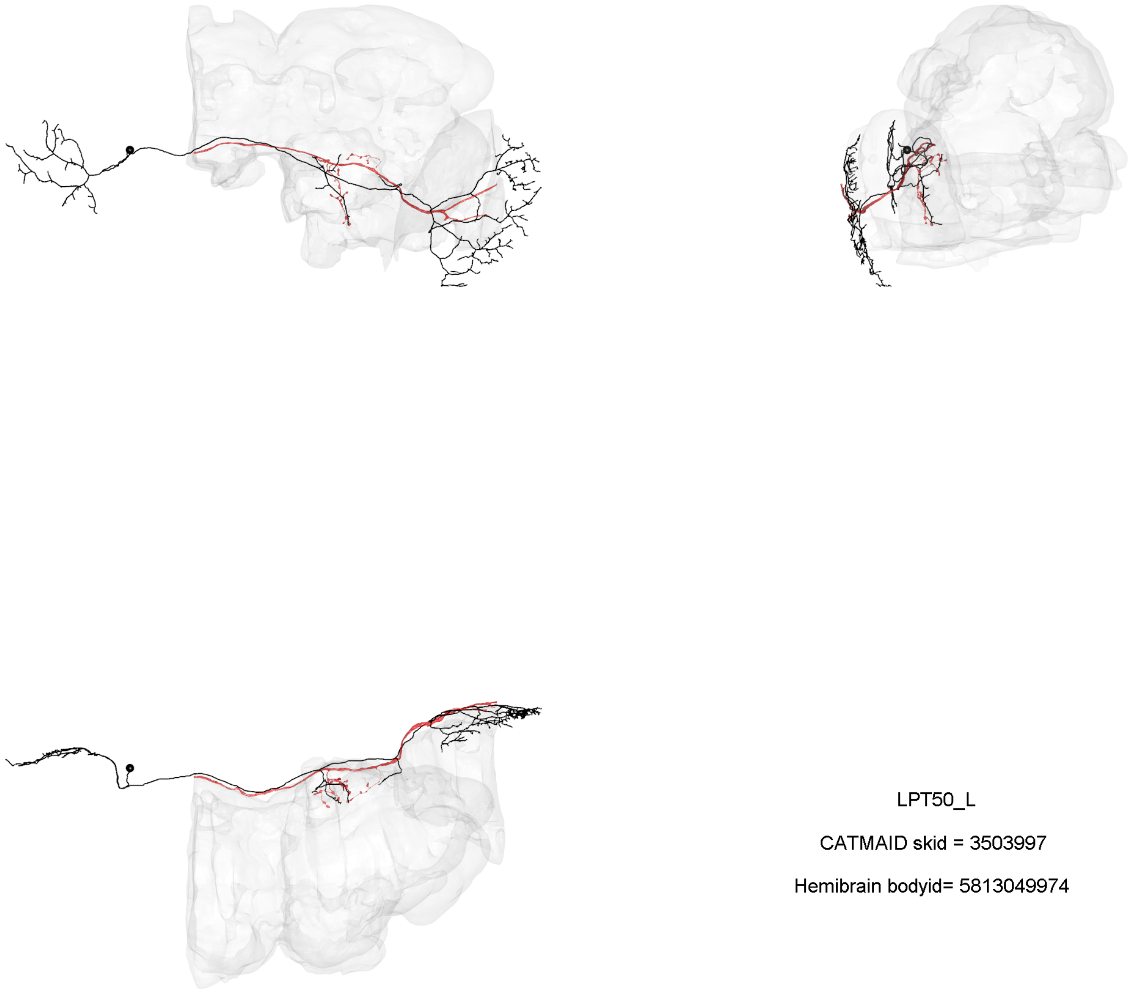

LPT50_L

CATMAID skid = 3503997

Hemibrain bodyid= 5813049974

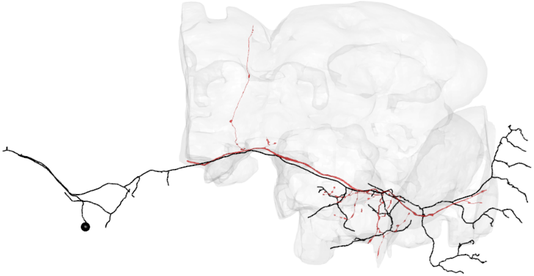

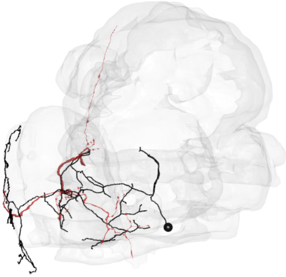

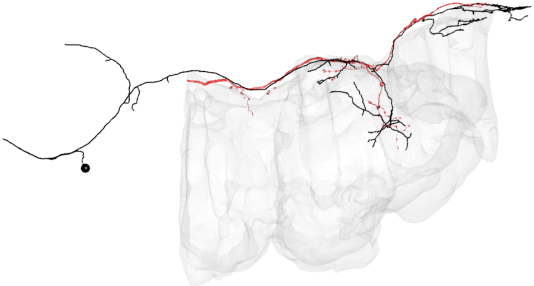

LPT56_L_MeLo1

CATMAID skid = 4224594

Hemibrain bodyid= 5813063227

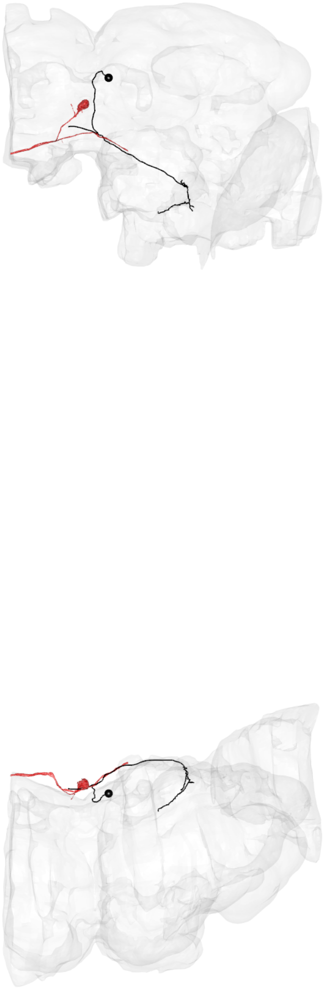

LPT57_L

CATMAID skid = 17059178

Hemibrain bodyid= 5813045086
