# Supplementary material for: A comprehensive neuroanatomical survey of the Drosophila Lobula Plate Tangential Neurons with predictions for their optic flow sensitivity
Source: bioRxiv. 2023 Oct 17:2023.10.16.562634. Preprint. [Version 1] doi: 10.1101/2023.10.16.562634 (PMC10614863; doi:10.1101/2023.10.16.562634)
Supplement: Supplement 7 [file NIHPP2023.10.16.562634v1-supplement-7.pdf]

bioRxiv preprint doi: https://doi.org/10.1101/2023.10.16.562634; this version posted October 17, 2023. The copyright holder for this preprint (which was not certified by peer review) is the author/funder, who has granted bioRxiv a license to display the preprint in perpetuity. It is made available under aCC-BY 4.0 International license.

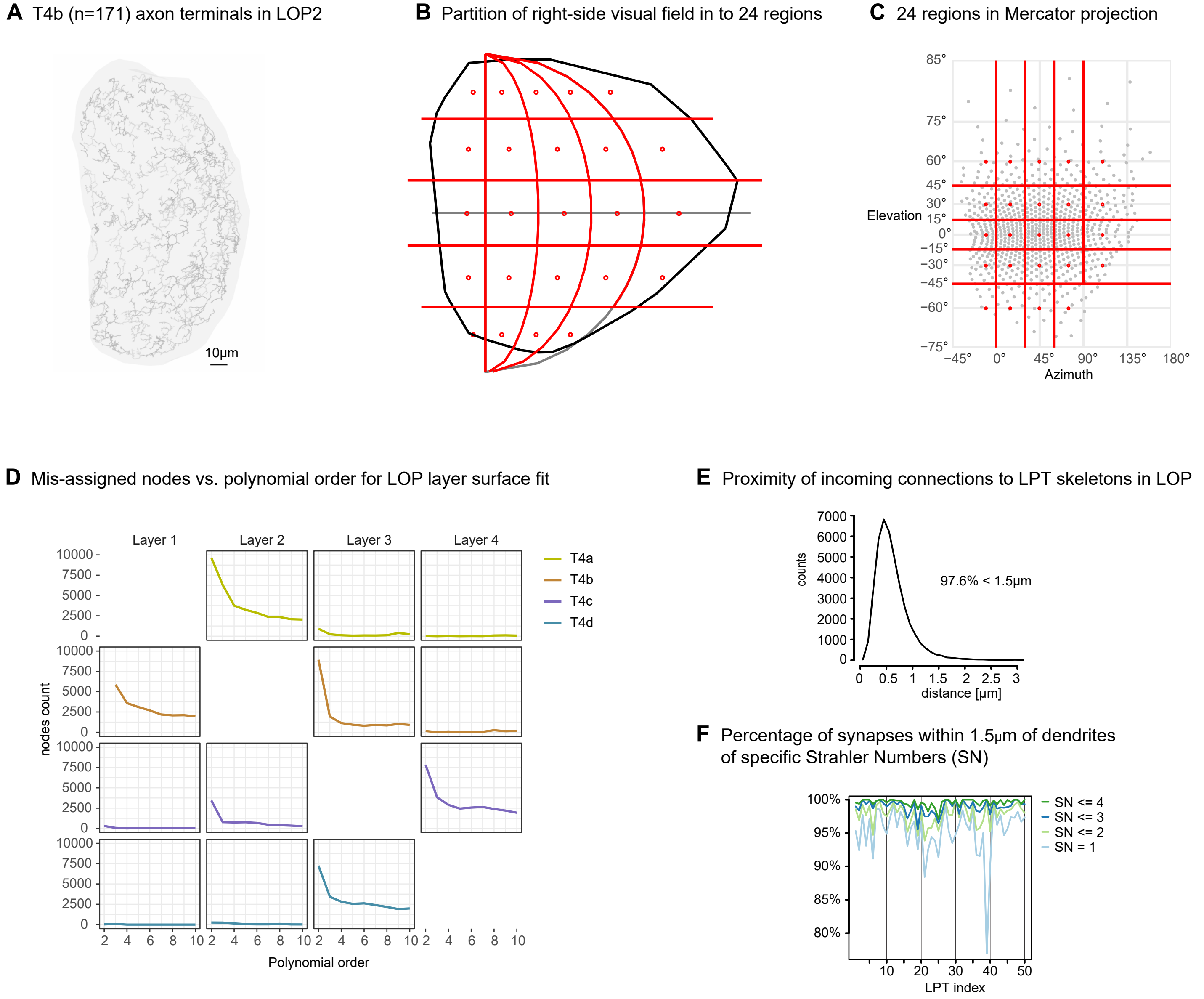

Figure 2 - figure supplement 1

bioRxiv preprint doi: https://doi.org/10.1101/2023.10.16.562634; this version posted October 17, 2023. The copyright holder for this preprint (which was not certified by peer review) is the author/funder, who has granted bioRxiv a license to display the preprint in perpetuity. It is made available under aCC-BY 4.0 International license.

Figure 2—figure supplement 1: Analysis details supporting the computational predictions of LPT PMPS.

A. EM reconstructed T4b axon terminals in LOP2. **B** and **C.** To present down-sampled PMPMs, the field of view of the fly's right eye is divided into 24 regions. The average preferred direction in each region is represented by arrows in the PMPMs, whose locations are denoted by red dots here. This averaging is carried out for all flow field plots in this manuscript. Here we show both Mollweide and Mercator projections. **D.** For the analysis presented in the manuscript, the LOP layers are constructed as a polynomial surface fit to the axon terminals of each T4 subtype. We use 5th order polynomials, and evaluated the quality of assignments of nodes in T4 axon terminals to layers to avoid over-fitting. The mis-assigned nodes are plotted as a function of the order of the polynomial surface fit. **E.** Distance between annotated synaptic connector and the nearest node of the reconstructed neuron skeleton, for all LPT neurons described in this paper. Note that 97.6% of connectors fall within 1.5 μm of the skeleton. **F.** Percentage of synapses captured by including varying levels of Strahler branches of the dendritic tree, within this 1.5 μm distance, for the main LPTs described in this manuscript (except for the feedback neurons that are not major T4/T5 targets). Note that SN <=3 captures most synapses, using SN>=4 may lead to overestimate of the dendritic coverage.

bioRxiv preprint doi: https://doi.org/10.1101/2023.10.16.562634; this version posted October 17, 2023. The copyright holder for this preprint (which was not certified by peer review) is the author/funder, who has granted bioRxiv a license to display the preprint in perpetuity. It is made available under aCC-BY 4.0 International license.

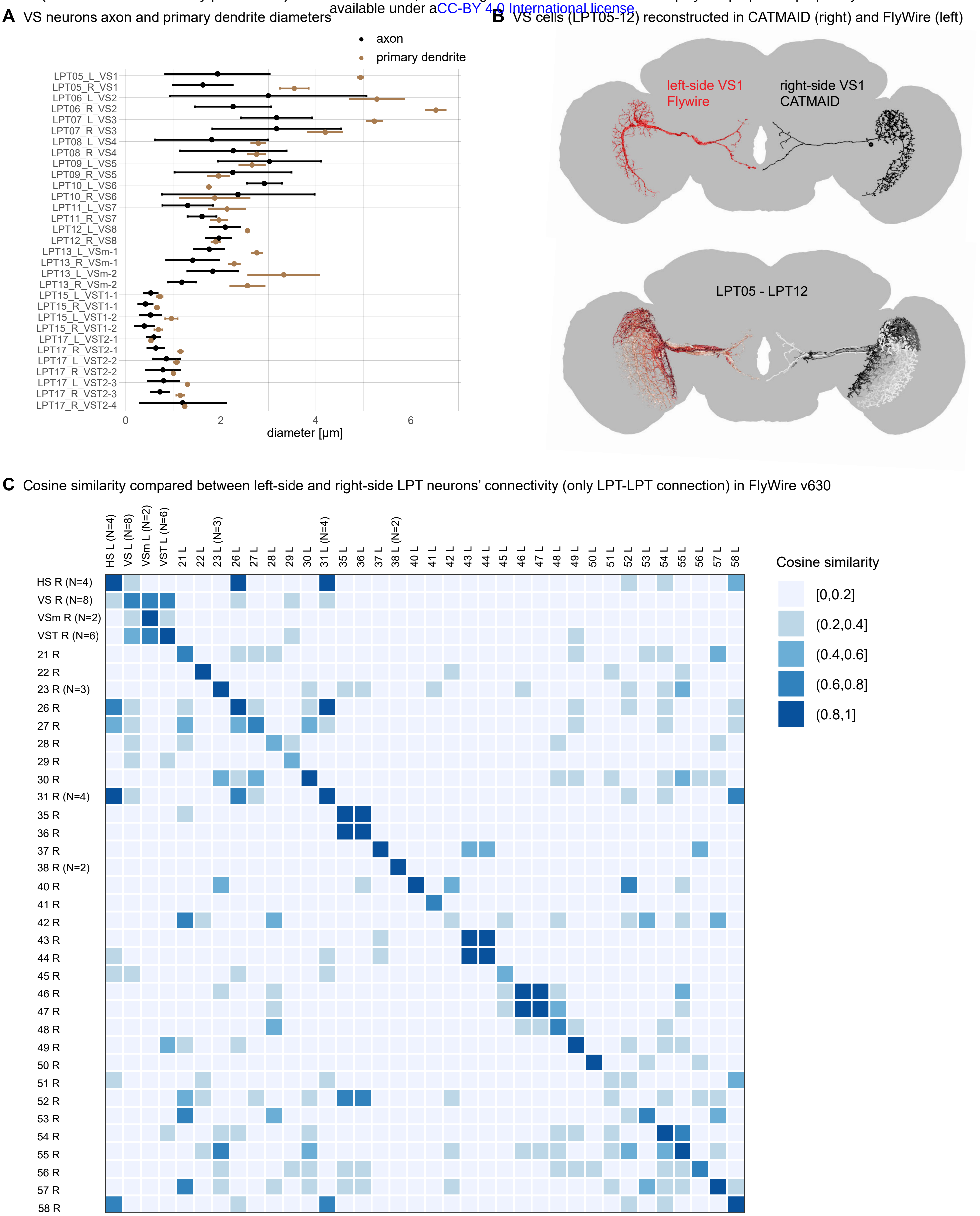

Figure 3 - figure supplement 1

bioRxiv preprint doi: https://doi.org/10.1101/2023.10.16.562634; this version posted October 17, 2023. The copyright holder for this preprint (which was not certified by peer review) is the author/funder, who has granted bioRxiv a license to display the preprint in perpetuity. It is made available under aCC-BY 4.0 International license.

Figure 3—figure supplement 1: Supporting evidence for identification of LPT neurons

A. Axon and primary dendrite diameters for all VS neurons in FAFB. We note that 16 VS cells were eventually found on the right and left sides of the brain, but at the time these measurements were made, one of the LPT17 cells on the left side had not yet been located. **B**. Comparison of EM reconstruction of VS neurons, in the FAFB brain, left-side neurons from FlyWire (in shades of red) and right-side neurons manually reconstructed in CATMAID (in shades of black). **C.** Cosine-similarity comparison of left vs. right-side LPT neurons in Flywire. Some cells are treated as a group, as indicated, and the cosine similarity is computed on the group connectivity average. For this computation, an LPT neuron's connectivity is characterized by its connection with all other LPT neurons only. All FlyWire data based on the March 2023 public release (version 630).

bioRxiv preprint doi: https://doi.org/10.1101/2023.10.16.562634; this version posted October 17, 2023. The copyright holder for this preprint (which was not certified by peer review) is the author/funder, who has granted bioRxiv a license to display the preprint in perpetuity. It is made available under aCC-BY 4.0 International license.

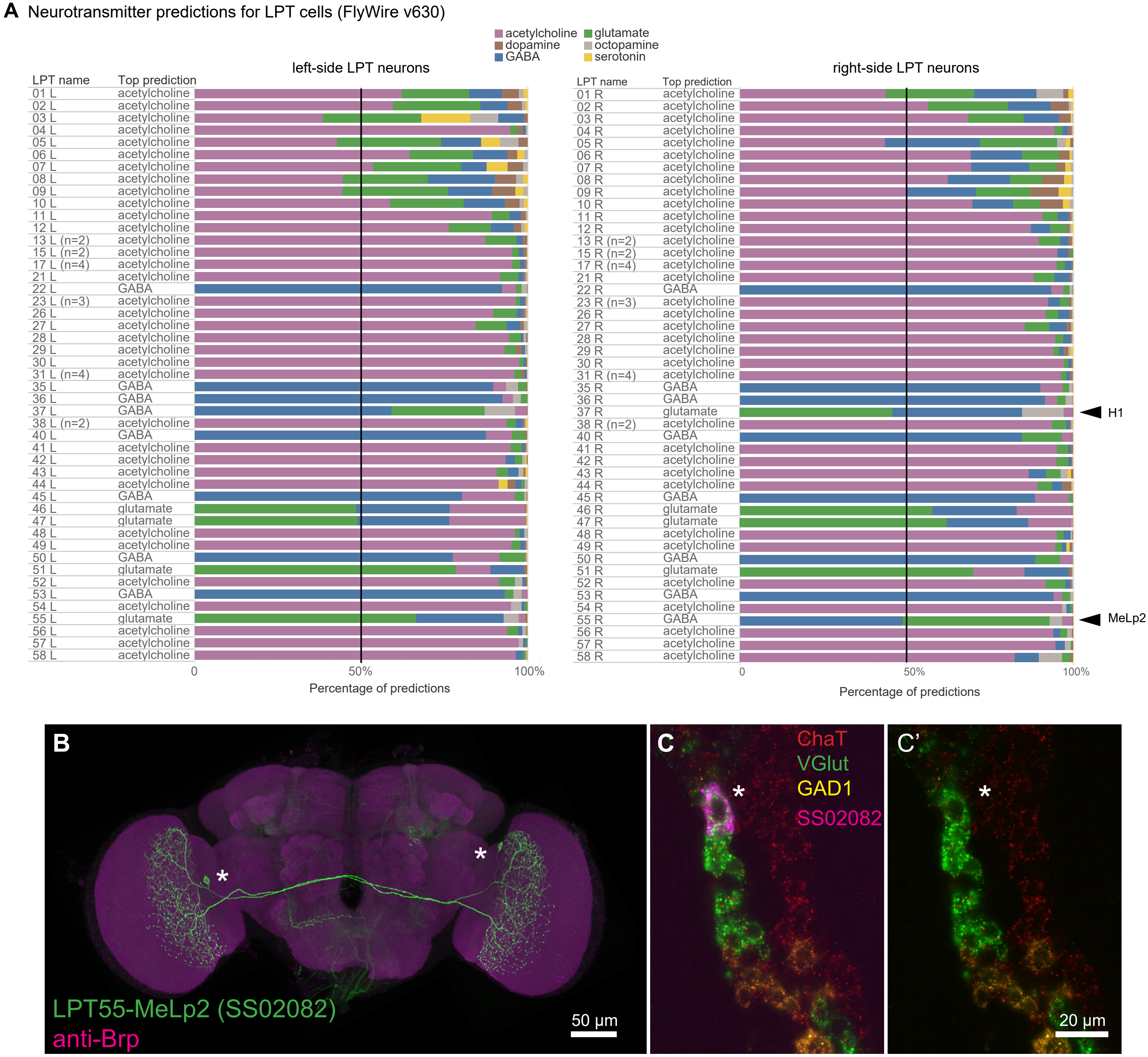

Figure 3 - figure supplement 2

bioRxiv preprint doi: https://doi.org/10.1101/2023.10.16.562634; this version posted October 17, 2023. The copyright holder for this preprint (which was not certified by peer review) is the author/funder, who has granted bioRxiv a license to display the preprint in perpetuity. It is made available under aCC-BY 4.0 International license.

Figure 3—figure supplement 2: Neurotransmitter predictions for the LPT neurons.
A. Neurotransmitter prediction for all LPT neurons, based on the method of (Eckstein et al., 2020) for the matched neurons in FlyWire. Only 2 cell types show right/left discrepancy in the top prediction, indicated with arrowheads. **B,C,C'.** Experimental evidence that MeLp2 neurons are glutamatergic. **B.** Expression pattern of a split-GAL4 line (SS02082) labelling LPT55-MeLp2 neurons. GAL4-driven expression of a membrane-targeted GFP is in green, a general neuropile label (anti-Brp) in magenta. The asterisks show the soma locations of the right and left MeLp2 neuron. **C and C'.** EASI-FISH labeling (Eddison and Ihrke, 2022; Wang et al., 2021) of transcripts indicative of cholinergic (ChAT; red), glutamatergic (VGlut; green) or GABAergic (GAD1; yellow) transmitter phenotypes. A MeLp2 cell body (indicated by the adjacent asterisk in both C and C') is labeled in magenta in C. Images show different channel combinations of a single confocal section.

bioRxiv preprint doi: https://doi.org/10.1101/2023.10.16.562634; this version posted October 17, 2023. The copyright holder for this preprint (which was not certified by peer review) is the author/funder, who has granted bioRxiv a license to display the preprint in perpetuity. It is made available under aCC-BY 4.0 International license.

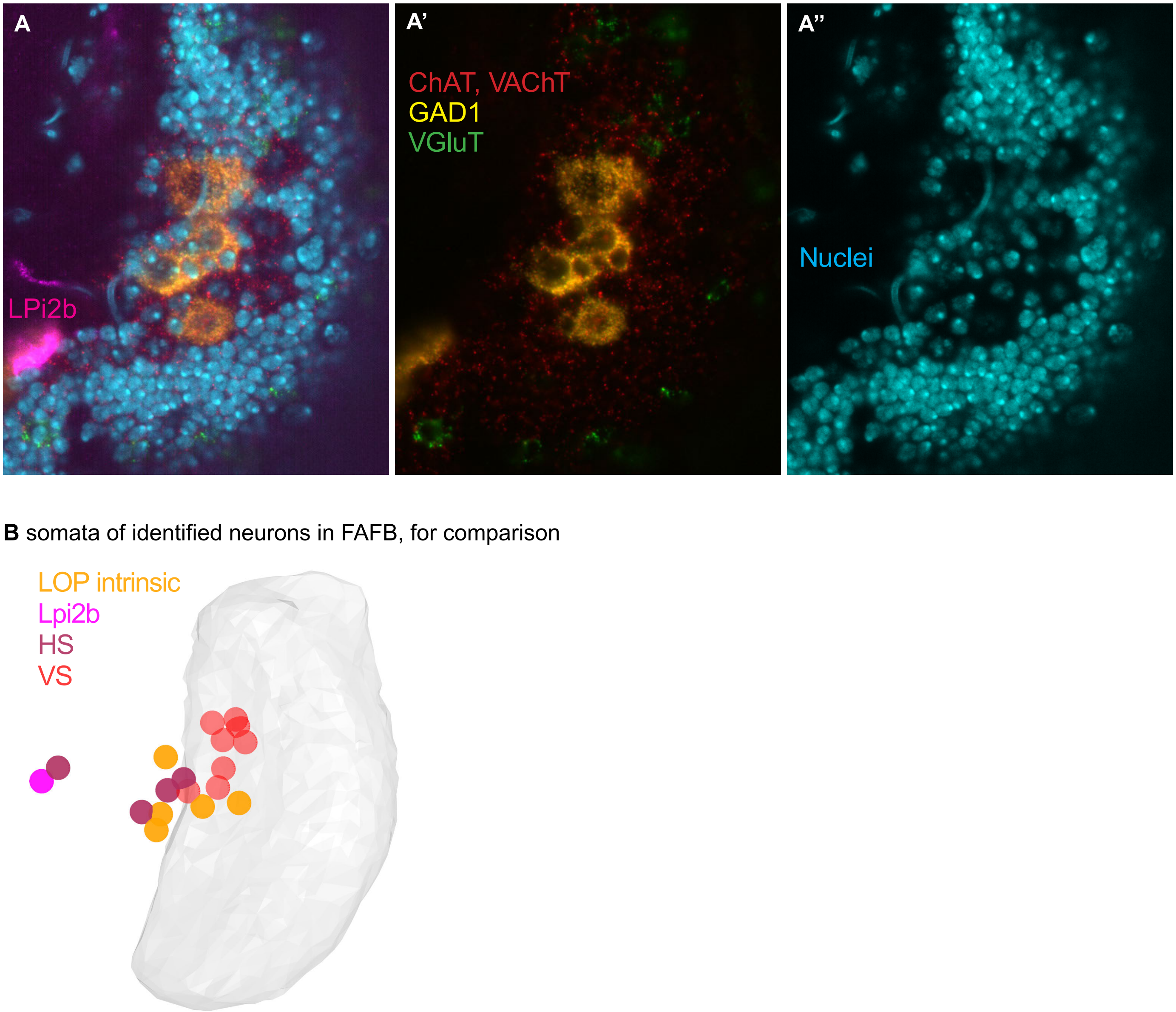

Figure 3 - figure supplement 3

bioRxiv preprint doi: https://doi.org/10.1101/2023.10.16.562634; this version posted October 17, 2023. The copyright holder for this preprint (which was not certified by peer review) is the author/funder, who has granted bioRxiv a license to display the preprint in perpetuity. It is made available under aCC-BY 4.0 International license.

Figure 3—figure supplement 3: Additional supporting evidence for the neurotransmitter predictions.

A ,A',A''. EASI-FISH labeling (Eddison and Ihrke, 2022; Wang et al., 2021) of ChAT (red), VGlut (green) and GAD1 (yellow) in the lobula plate cell body rind, in the region where the HS and VS somata are found. Nuclei are also labeled (blue). An LPi2b neuron labelled by a split-GAL4 driver line (SS 53141) is shown in magenta.

Images show different channel combinations of a single confocal section (A, all channels; A' transmitter indicators only; A'', nuclei only). Large cells in this region appear to be either GABAergic (GAD1 marker, orange) or cholinergic (ChAT marker, red). **B.** For comparison with (A), we use the soma locations from the FAFB LPT set: LPi2b (magenta), other large optic lobe intrinsic LOP neurons (orange; these include Am1, 2 LPi12, Lpi21, and an undescribed cell that appears to be a large layer four LPi), 4 HS neurons (maroon), and 8 VS neurons (red). The large optic lobe intrinsic neurons are only partially reconstructed and not included in the LPT survey reported in this study, but could be matched to known cell types (Shinomiya et al., 2022). Given that these are all the large cell bodies we found in this region, and several of the LPis are expected to be inhibitory, we suggest that the GABAergic cells in the EASI-FISH images are the large LPi and Am1 neurons and the cholinergic cells are the HS and large VS cells.

bioRxiv preprint doi: https://doi.org/10.1101/2023.10.16.562634; this version posted October 17, 2023. The copyright holder for this preprint (which was not certified by peer review) is the author/funder, who has granted bioRxiv a license to display the preprint in perpetuity. It is made available under aCC-BY 4.0 International license.

Morphology and LOP coverage for non-T4-input LPT neurons

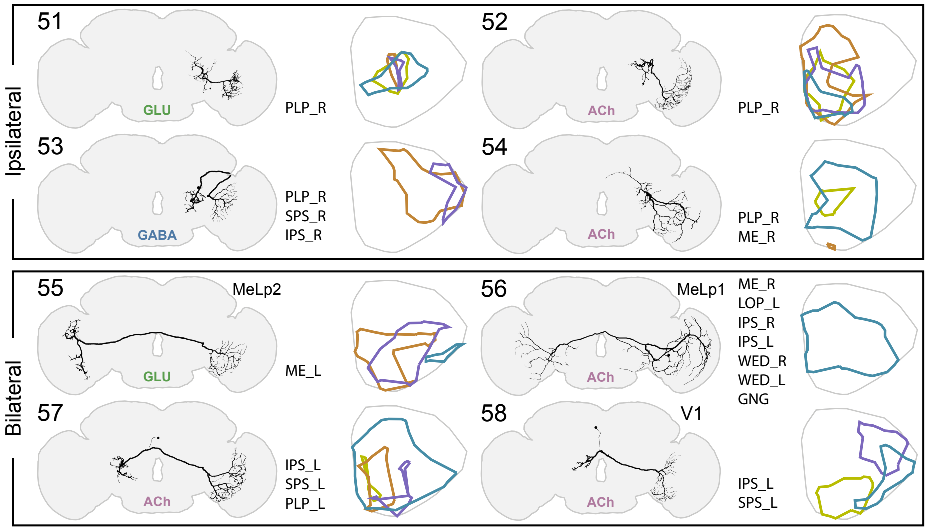

Figure 4 - figure supplement 1

bioRxiv preprint doi: https://doi.org/10.1101/2023.10.16.562634; this version posted October 17, 2023. The copyright holder for this preprint (which was not certified by peer review) is the author/funder, who has granted bioRxiv a license to display the preprint in perpetuity. It is made available under aCC-BY 4.0 International license.

Figure 4—figure supplement 1: morphology and layer coverage of the LOP-input LPT neurons

The remaining LPT neurons receive little T4 inputs, instead they most likely provide inputs to the LOP, some as feedback from the central brain. As these neurons receive minimal T4 inputs, we do not predict their motion pattern maps.

bioRxiv preprint doi: https://doi.org/10.1101/2023.10.16.562634; this version posted October 17, 2023. The copyright holder for this preprint (which was not certified by peer review) is the author/funder, who has granted bioRxiv a license to display the preprint in perpetuity. It is made available under aCC-BY 4.0 International license.

A Optimal axes of rotation based on PMPM

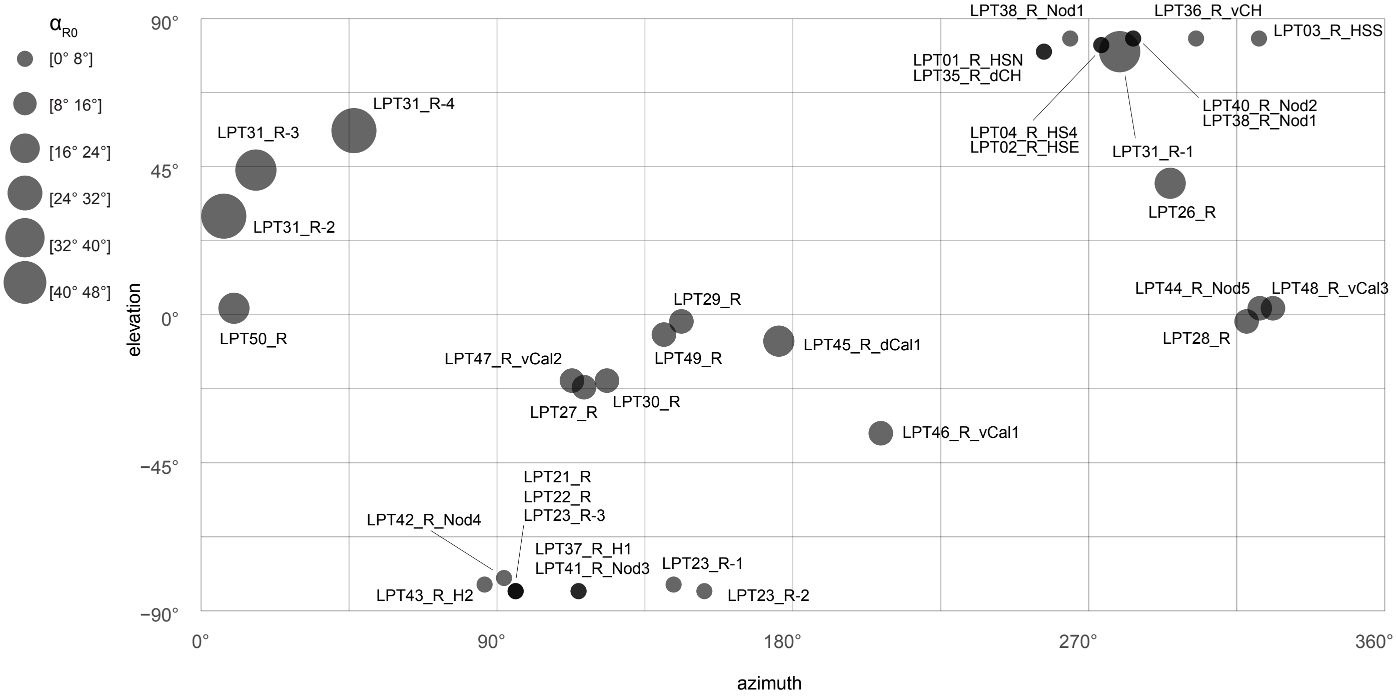

B Optimal axes of translation based on PMPM

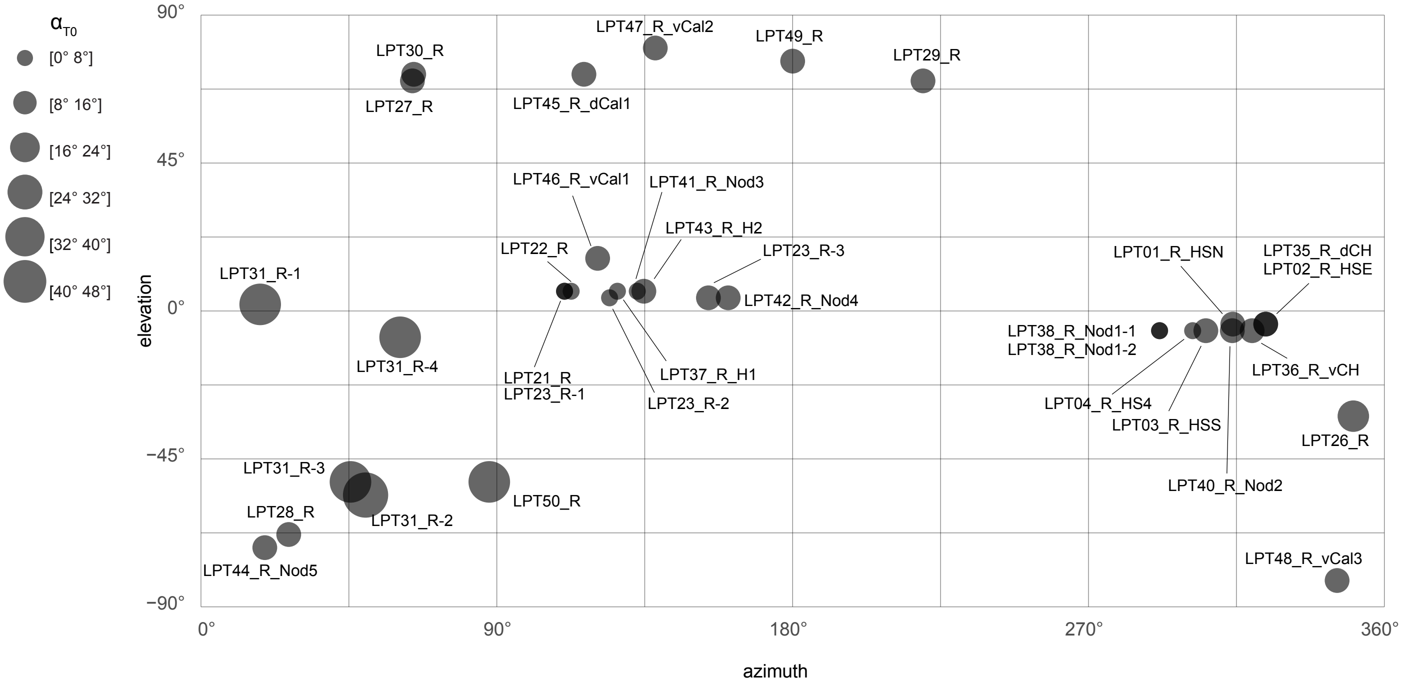

Figure 4 - figure supplement 2

bioRxiv preprint doi: https://doi.org/10.1101/2023.10.16.562634; this version posted October 17, 2023. The copyright holder for this preprint (which was not certified by peer review) is the author/funder, who has granted bioRxiv a license to display the preprint in perpetuity. It is made available under aCC-BY 4.0 International license.

Figure 4—figure supplement 2: Optimal rotation or translation axes predicted by PMPMs. These two plots summarize the optimal rotation (top) and translation (bottom) axes for the LPTs of Figures 3 and 4, excluding the VS neurons, which are treated in Figure 3B. The size of each neuron's marker indicates the size of the angular error.

bioRxiv preprint doi: https://doi.org/10.1101/2023.10.16.562634; this version posted October 17, 2023. The copyright holder for this preprint (which was not certified by peer review) is the author/funder, who has granted bioRxiv a license to display the preprint in perpetuity. It is made available under aCC-BY 4.0 International license.

A Improvement in connectivity completeness after additional proofreading

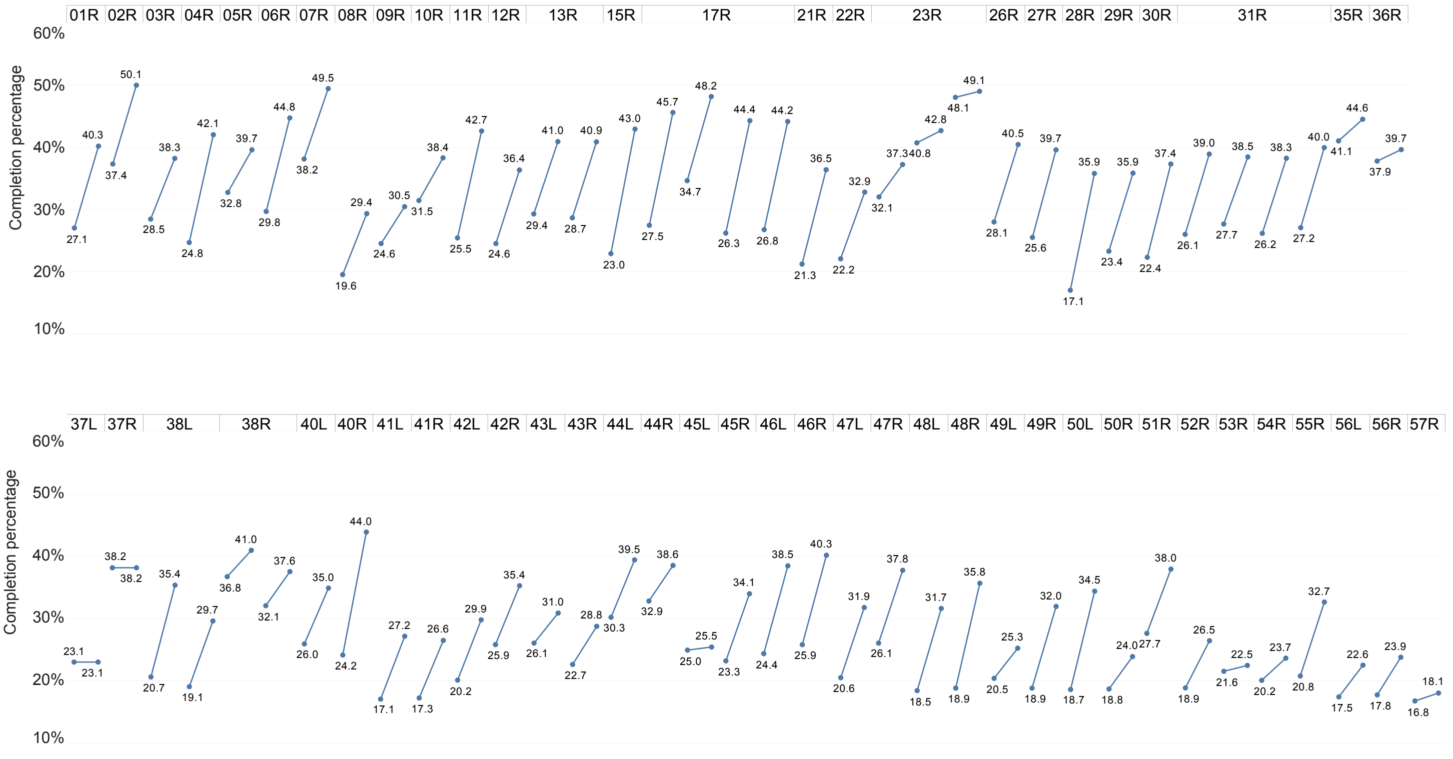

B Comparison of LPT-LPT central brain connectivity in FAFB vs. Hemibrain

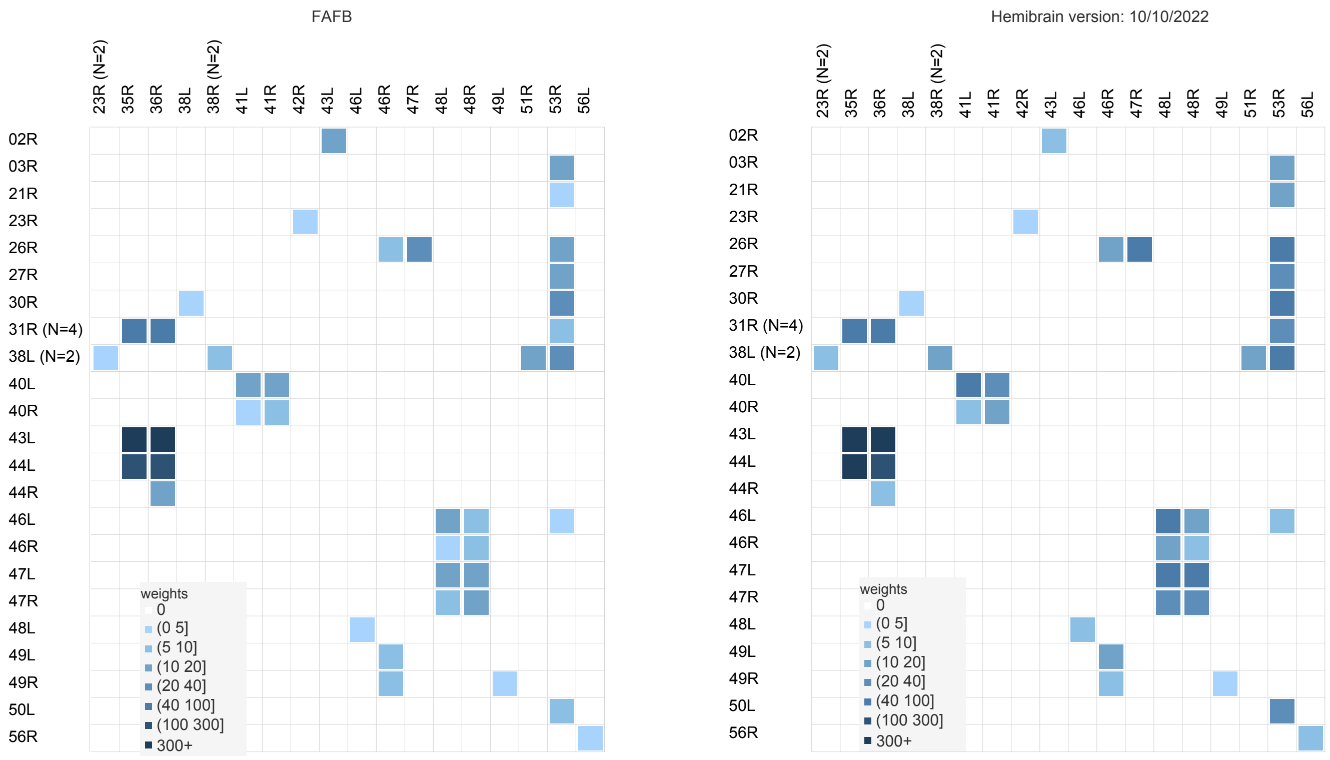

Figure 5 - figure supplement 1

bioRxiv preprint doi: https://doi.org/10.1101/2023.10.16.562634; this version posted October 17, 2023. The copyright holder for this preprint (which was not certified by peer review) is the author/funder, who has granted bioRxiv a license to display the preprint in perpetuity. It is made available under aCC-BY 4.0 International license.

Figure 5—figure supplement 1: LPT connectivity in the Hemibrain connectome

A. Improvement in connectivity completeness for the matched set of LPT neurons, after manual proofreading in Hemibrain. **B.** Comparison of central brain LPT-LPT connectivity in FAFB (left) and Hemibrain (right) data sets.

bioRxiv preprint doi: https://doi.org/10.1101/2023.10.16.562634; this version posted October 17, 2023. The copyright holder for this preprint (which was not certified by peer review) is the author/funder, who has granted bioRxiv a license to display the preprint in perpetuity. It is made available under aCC-BY 4.0 International license.

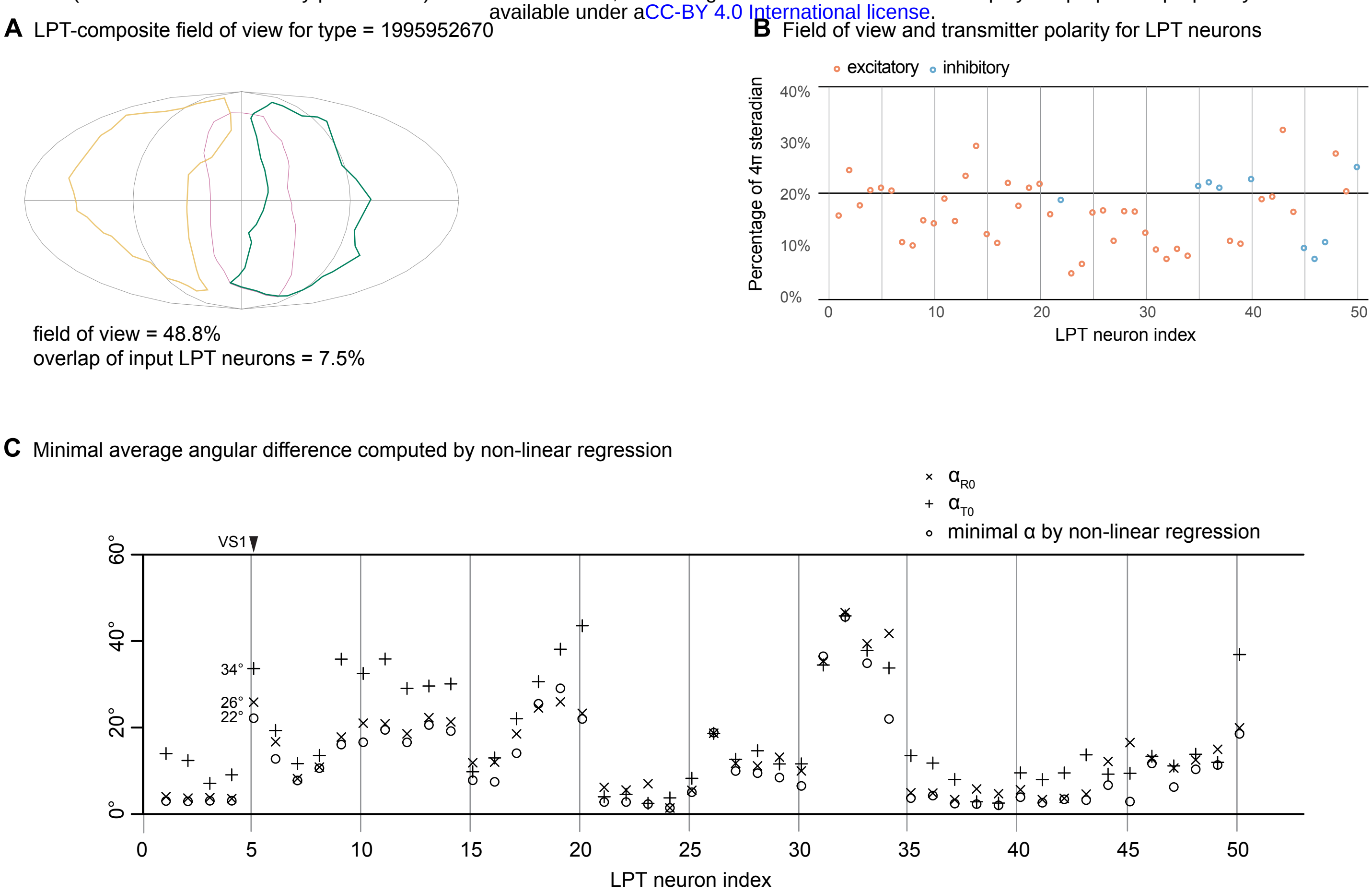

Figure 6 - figure supplement 1

bioRxiv preprint doi: https://doi.org/10.1101/2023.10.16.562634; this version posted October 17, 2023. The copyright holder for this preprint (which was not certified by peer review) is the author/funder, who has granted bioRxiv a license to display the preprint in perpetuity. It is made available under aCC-BY 4.0 International license.

Figure 6—figure supplement 1: supporting data for LPT integration analysis

A. Composite field of view for the example LPT target neuron in Figure 6A. **B**. Fields of view and predicted synapse polarity for the LPT neurons (the indexing used throughout indicates the corresponding LPT neuron). **C**. Comparing the minimal average angular differences computed via the brute force method for rotation and translation separately, and a non-linear regression method treating them jointly (see Methods).

bioRxiv preprint doi: https://doi.org/10.1101/2023.10.16.562634; this version posted October 17, 2023. The copyright holder for this preprint (which was not certified by peer review) is the author/funder, who has granted bioRxiv a license to display the preprint in perpetuity. It is made available under aCC-BY 4.0 International license.

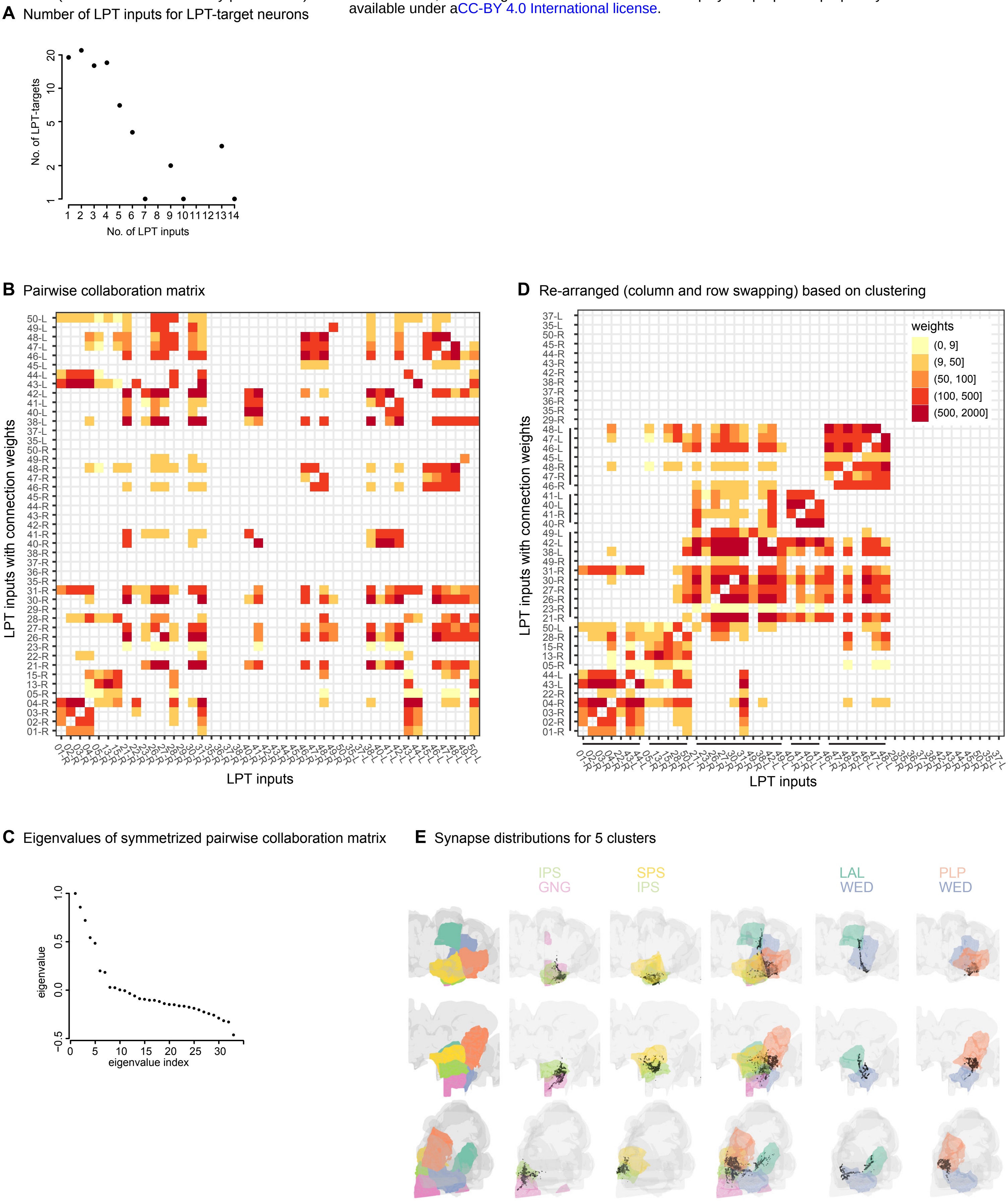

Figure 6 - figure supplement 2

bioRxiv preprint doi: https://doi.org/10.1101/2023.10.16.562634; this version posted October 17, 2023. The copyright holder for this preprint (which was not certified by peer review) is the author/funder, who has granted bioRxiv a license to display the preprint in perpetuity. It is made available under aCC-BY 4.0 International license.

Figure 6—figure supplement 2: Patterns of LPT output integration in the central brain

A. Number of target cells vs. number of their LPT inputs for the central neurons considered in this analysis (based on Hemibrain connectivity). **B**. Pairwise "collaboration matrix" where each entry represents all the LPT-target neurons that receive inputs from the LPT neuron indicated in the row and the LPT neuron in that column. The matrix is asymmetric since the value of each entry is the total connection weight from the LPT neuron in the row. **C.** The eigen spectrum of the Laplacian matrix constructed from the pairwise collaboration matrix, showing a gap after the 5th largest eigenvalue. This was used to select the number of clusters (=5) for the spectral clustering in C. **D.** The pairwise collaboration matrix re-ordered based on spectral clustering. There are 5 clusters within the connected graph. **E.** Synapse distributions and the relevant neuropils capturing the majority of synapses for each cluster in the central brain. The synapses shown are restricted to those between LPT neurons and their targets (i.e. those in the collaboration matrix).
